# Climate change impacts on marine biodiversity, fisheries and society in the Arabian Gulf

**DOI:** 10.1371/journal.pone.0194537

**Published:** 2018-05-02

**Authors:** Colette C. C. Wabnitz, Vicky W. Y. Lam, Gabriel Reygondeau, Lydia C. L. Teh, Dalal Al-Abdulrazzak, Myriam Khalfallah, Daniel Pauly, Maria L. Deng Palomares, Dirk Zeller, William W. L. Cheung

**Affiliations:** 1 Nippon Foundation-Nereus Program and Changing Ocean Research Unit, Institute for the Oceans and Fisheries, The University of British Columbia, Vancouver, BC, Canada; 2 *Sea Around Us*, Institute for the Oceans and Fisheries, The University of British Columbia, Vancouver, BC, Canada; 3 *Sea Around Us*–Indian Ocean, School of Biological Sciences, University of Western Australia, Crawley, WA, Australia; University of Sydney, AUSTRALIA

## Abstract

Climate change–reflected in significant environmental changes such as warming, sea level rise, shifts in salinity, oxygen and other ocean conditions–is expected to impact marine organisms and associated fisheries. This study provides an assessment of the potential impacts on, and the vulnerability of, marine biodiversity and fisheries catches in the Arabian Gulf under climate change. To this end, using three separate niche modelling approaches under a ‘business-as-usual’ climate change scenario, we projected the future habitat suitability of the Arabian Gulf (also known as the Persian Gulf) for 55 expert-identified priority species, including charismatic and non-fish species. Second, we conducted a vulnerability assessment of national economies to climate change impacts on fisheries. The modelling outputs suggested a high rate of local extinction (up to 35% of initial species richness) by 2090 relative to 2010. Spatially, projected local extinctions are highest in the southwestern part of the Gulf, off the coast of Saudi Arabia, Qatar and the United Arab Emirates (UAE). While the projected patterns provided useful indicators of potential climate change impacts on the region’s diversity, the magnitude of changes in habitat suitability are more uncertain. Fisheries-specific results suggested reduced future catch potential for several countries on the western side of the Gulf, with projections differing only slightly among models. Qatar and the UAE were particularly affected, with more than a 26% drop in future fish catch potential. Integrating changes in catch potential with socio-economic indicators suggested the fisheries of Bahrain and Iran may be most vulnerable to climate change. We discuss limitations of the indicators and the methods used, as well as the implications of our overall findings for conservation and fisheries management policies in the region.

## Introduction

Marine biodiversity, ecosystem health and fisheries are currently threatened by overfishing, but also by pollution and other anthropogenic impacts [1]. Climate change further challenges our ability to devise sustainable management and conservation plans to maintain ecosystem services, as it has begun to alter ocean conditions, particularly water temperature and various aspects of ocean biogeochemistry [2]. Marine biodiversity responds to shifting temperatures and other ocean conditions through changes in organismal physiology and phenology, as well as population dynamics and distributions [3–5]. These responses to ocean–atmospheric changes have been projected to lead to altered patterns of species richness [6, 7], changes in community structure [8] and ecosystem functions [9], and consequential changes in marine goods and services [10–12].

Given the unique characteristics of the Arabian Gulf (also known as the Persian Gulf, and referred to hereafter simply as the Gulf)—particularly its extreme environmental conditions, the array of human disturbances it is exposed to, and the high sensitivity of its biota to environmental fluctuations as species are close to their environmental limits [6, 13]—climate change should have substantial implications for the Gulf’s marine ecosystems and fisheries. Extreme seasonal temperatures and salinity fluctuations select for species with high tolerance or adaptability to such short-term changes (e.g., as exhibited by some corals, see [14]). Consequently, the Gulf is a region that is relatively species poor [15–18], at least in comparison with adjacent systems such as the open Indian Ocean [17]. However, as part of the Western Indian Ocean province of the Indo-West Pacific ecoregion [19], the Gulf is considered a biologically valuable region [20]. The region’s biodiversity and its associated goods and services are expected to be impacted by the synergistic effects of climate change (e.g., increases in temperature; declines in oxygen content; sea level rise) and those of human activities such as oil extraction, desalination of sea water, coastal development, and overfishing [21–25].

Although many marine organisms in the Gulf appear to have a high heat-tolerance relative to populations in other parts of the world [26–29], warming, with changes of +0.57°C recorded between 1950 and 2010 [30], has already impacted some of the more vulnerable marine species in the region [21]. For example, corals have been exposed to major disturbances [31], including water temperatures between 35° and 37°C at least five times since the late 1990s, causing extensive coral bleaching [32] associated with considerable loss of coral cover [33, 34]. Overall, about 70% of the Gulf’s reefs have essentially disappeared in a few decades [35] and this has been associated with a significant decline in fish species richness. While substantial declines in stress-sensitive species are expected with increasing temperatures, results from a number of long-term studies investigating benthic community structure across the region suggest that coral communities may persist within an increasingly disturbed future environment, albeit in a much more structurally simple configuration [27, 31, 36].

So far, a comprehensive assessment of climate change impacts on the Gulf’s marine biodiversity and fisheries has not been undertaken. By means of simulation modelling approaches, this study aims to assess the impacts and understand the vulnerability of some of the Gulf’s key marine species, its fisheries and national economies to climate change. We then discuss the implications of these impacts for conservation and fisheries management policies in the region.

## Materials and methods

### Study area

**Fig 1:**
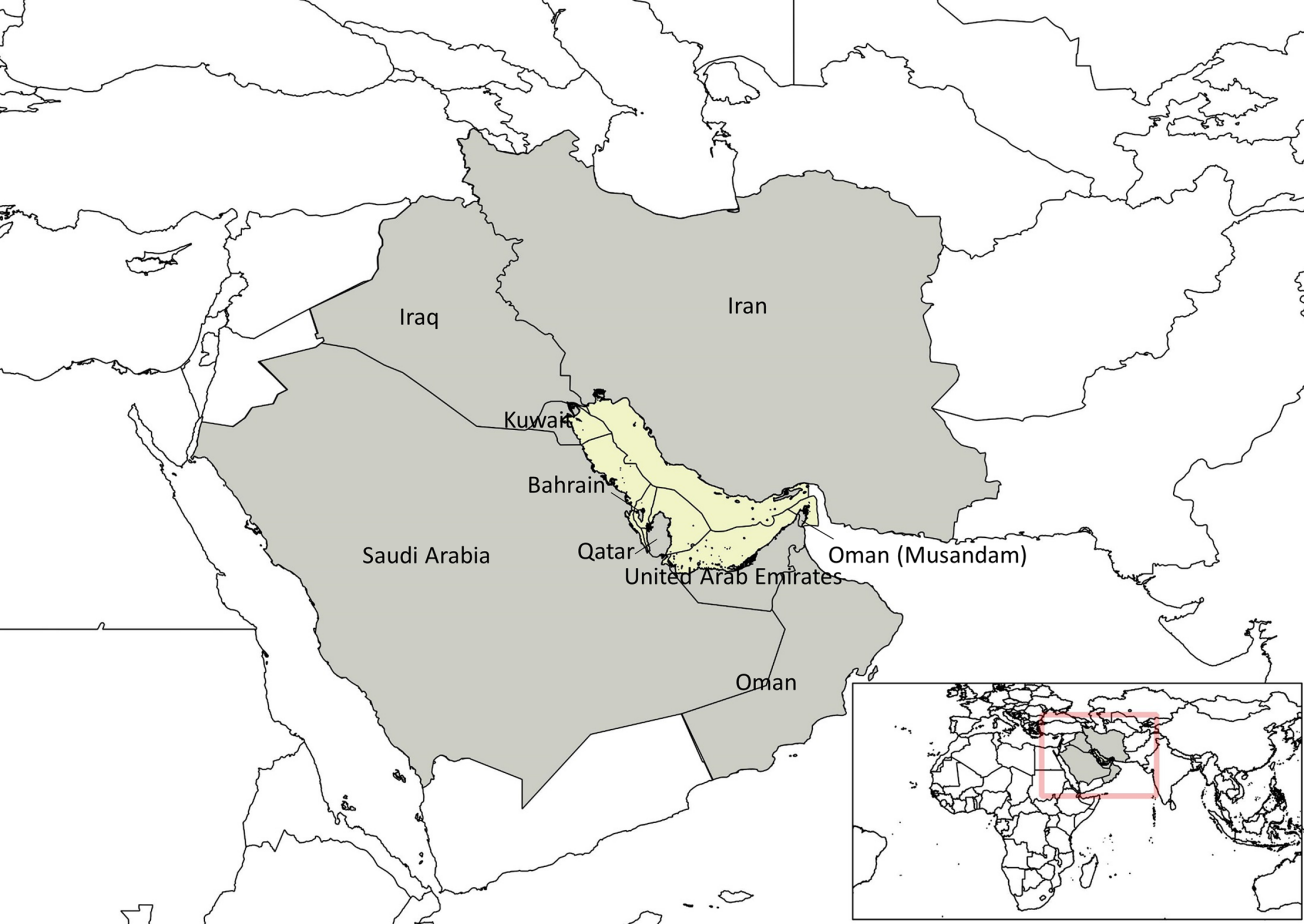
The Gulf as defined in this study. The map shows the approximate extent of actual and/or claimed Exclusive Economic Zones (EEZs) as used here, notably to allocate fisheries catches. Note that the maritime limits and boundaries shown on this map are not authoritative regarding the delimitation of international maritime boundaries. Source: Natural Earth version 4.0.0 - http://www.naturalearthdata.com/. Map created using QGIS 2.8.2 –Wien.

The Gulf is bordered by Bahrain, Iran, Iraq, Kuwait, Oman, Qatar, Saudi Arabia and the United Arab Emirates (UAE), all signatory members of the Regional Organization for the Protection of the Marine Environment (ROPME), created in 1978. It is bounded in the north, for the most part, by the coast of Iran with the Shatt al-Arab river delta at the western end, and in the south, mainly by the coasts of Saudi Arabia, with the eastern end being the north-western limit of the Gulf of Oman at the Strait of Hormuz (24^o^ to 30^o^30'N; 48^o^ to 56^o^25'E1; see Fig 1).

Ecologically, the Gulf is a relatively shallow semi-enclosed marginal sea with a depth range of 10 to 93 m, averaging 36 m, a length of 990 km, a width ranging between 56 km and 370 km, and a total surface area of 239,000 km^2^ [37]. It has a gently sloping terraced shelf punctuated by numerous islands that formed as part of an extensive sabkha (i.e., salt flat [38]). Water temperature ranges from 20^o^ C in winter to more than 30^o^ C in summer, with maximum salinities of 48 psu [14], averaging 40 psu [39], and exceeding 70 psu in lagoons (e.g., in Saudi Arabia) [15]. Freshwater influx into the Gulf originates from 200 underground water springs, 25 springs from the Zagros Mountain, and 8 major rivers, notably the Euphrates and Tigris, which merge into the Shatt al Arab before flowing into the Gulf. These physical and environmental conditions make the Gulf a sedimentary environment [40] that is conducive to the growth of mangroves, algae and seagrass, providing refuge and forage for a multitude of marine species, and also protecting the coastline from degradation [41].

Primary productivity is high at certain times of the year, with an increasing gradient in phytoplankton species richness and biomass from the Shatt Al-Arab area (low species diversity, high biomass and production) to Kuwait, the Gulf of Oman, and the Strait of Hormuz (high species diversity, low biomass and production [42]).

### Projecting climate change impacts on marine biodiversity

To assess the impact of climate change on the Gulf’s marine biodiversity, we updated available information on the ecology of 55 ‘priority species’, identified based on their contribution to catches with additional species selected by regional stakeholders (i.e., governments, researchers, NGOs) that were part of the Local, National, and Regional Climate Change (LNRCC) Programme of the Abu Dhabi Global Environmental Data Initiative (AGEDI). The programme is stakeholder-driven with over 100 members, and feedback was parsed through two programme coordinators, with a large number of members particularly concerned about sea turtles and marine mammals. The selected priority species included 47 of the most important fish and invertebrate species to fisheries in the Gulf (by weight), important biogenic features for marine biodiversity (three species of seagrasses), and charismatic non-fish species that are also vulnerable or endangered, such as the hawksbill (*Eretmochelys imbricata*) and green turtles (*Chelonia mydas*), the dugong (*Dugong dugon*), and two species of dolphins (*Sousa chinensis*, *Tursiops aduncus*) (S1 and S2 Tables).

The current and future distributions of the prioritized 55 marine species were here modelled using an environmental niche approach, *sensu* [43]. This method quantifies the environmental preferences (e.g., temperature, salinity, dissolved oxygen) of marine species and projects their potential distribution according to present and future conditions.

To model species’ environmental niches we collated global occurrence records and environmental data from a variety of sources. First, species presence/occurrence data were obtained from the Ocean Biogeographic System (OBIS, http://www.iobis.org, accessed in 2015) and the Global Biodiversity Information Facility (GBIF, http://www.gbif.org, accessed in 2015). All points that fell outside known environmental preferences and geographic limits, as defined in FishBase [44], SeaLifeBase [45] or obtained from OBIS-SEAMAP information (http://seamap.env.duke.edu/, accessed in 2015), were removed. Second, a set of environmental parameters known to influence marine species distributions were gathered at a global gridded scale. These included: sea surface temperature (SST) (1950–2013, [46]); sea bottom temperature (1950–2013, [46]); sea surface and bottom salinity (1950–2013, [46]); sea surface and bottom nutrient concentration (1950–2013, [46]); bathymetry (1950–2013, [46]); sea surface and bottom oxygen concentration (1950–2013, [46]); chlorophyll *a* concentration (2006–2015, [47]); particulate organic matter (2006–2015, [47]); and euphotic depth (2006–2015, [47]). The spatial data for each annual environmental climatology were re-gridded onto 0.25^o^ latitude x 0.25^o^ longitude resolution using a spline interpolation method [48].

The environmental niche of each species was quantified using three separate models: the Non-Parametric Probabilistic Ecological Niche (NPPEN) model [49]; the Bioclimate analysis and prediction (BIOCLIM) model [50], and the Ecological Niche Factor Analysis (ENFA) model [51].

First, for each of the 55 focal species, the models quantified individual species’ environmental envelope by estimating the best combination of environmental conditions, based on all of the parameters listed above, that describe its current global distribution. Sea surface and sea bottom environmental conditions were used for pelagic and demersal species, respectively. Second, we used these species-specific environmental envelopes to project the probability of occurrence of a given species in each spatial cell of the ocean according to environmental conditions associated with that cell. Third, using projected future sea surface temperature and salinity only, we projected ‘current’ (2000–2010), mid-21st century (2040–2050) and end of 21st century (2090–2100) species distributions, based on high-resolution modelled hydrological conditions (temperature and salinity) of the Gulf provided by the Regional Oceanographic Modelling group of the AGEDI LNRCC programme [52]. The oceanographic model projected changes under the Representative Concentration Pathway (RCP) 8.5, representing a high-greenhouse-gas-emissions, business-as-usual scenario [53]. For the current period, we calculated the spatial anomalies of the high resolution (0.0275^o^ latitude x 0.0275^o^ longitude) model outputs over a coarser resolution grid (0.25^o^ latitude x 0.25^o^ longitude). We attempted to use the best available information to correct for systematic biases between the coarser global-scale and the finer local-scale environmental data. Mesoscale patterns that are represented in the finer resolution dataset may be smoothed out when the data were aggregated to the coarser resolution. However, such mesoscale patterns are unlikely to dramatically alter the pattern of changes in projected habitat suitability averaged at the Exclusive Economic Zone (EEZ) levels in our vulnerability analysis. We then applied the spatial anomalies of both the current and future periods to the global environmental data described above to correct for the bias between modelled outputs and global data products from the synthesis of observational data. This procedure helped retain the high resolution spatial features of the model outputs. Next, we projected the spatial distribution of the 55 focal species using the three environmental niche models and the processed environmental model outputs (i.e., based on climatological annual averages of predicted changes in salinity and temperature). The projected current and future spatial distributions of each species were further limited to the known depth range of the species and their affinity to the coast.

Using results from projected changes in distributions, we estimated the impacts of climate change on the diversity of the 55 focal species using three indicators: rate of species invasion; rate of species local extinction; and sum of habitat suitability index (i.e., index of habitat biodiversity suitability (HBS)). Rate of species invasion was calculated as the number of species newly occurring in a cell by 2050 (average between 2040 and 2050) and 2090 (average between 2090 and 2100) relative to the number of species in that cell in 2010 (average between 2000 and 2010). Rate of species local extinction represents the number of species disappearing from a cell in 2050 and 2090 relative to the number of species in that cell in 2010. Note that both indicators evaluate “invasion” and “extinction” by comparing changes in temperature and salinity of a cell with species’ environmental envelopes. Whether a certain species actually invades a cell that falls within its climatology in the future may depend on factors outside of the scope of this study. Changes in HBS were estimated by subtracting the sum of the probability of occurrence for all species in 2050 and 2090 from that of 2010 for each cell. Note that from here on onwards, when referring to habitat changes, we imply changes in the combination of temperature and salinity in the future compared to present conditions. In this context, habitat does not denote biogenic features such as ‘reef’ or ‘seagrass’ for example, that hawksbill turtle or dugong, respectively, may typically associate with for refuge and/or forage. In other words, changes in habitat suitability for a species refers to the experienced combination of changes in salinity and temperature at a given point in time by that species, relative to its niche for those parameters, as defined by observed global occurrences. This is relevant for fish, which constitute the largest proportion of the priority species identified, as habitat tends to be determined by the water column for a significant portion of their life history, with two key environmental parameters of this habitat being temperature and salinity. For some species however, other factors may be more important in determining whether a given species’ realized niche will entirely fill its new possible range extent.

### Vulnerability of charismatic species

In this study we focused on the following charismatic species: dugong (*Dugong dugon*), Indo-Pacific humped-back dolphin (*Sousa chinensis*), Indo-Pacific bottlenose dolphin (*Tursiops aduncus*), green turtle (*Chelonia mydas*), and hawksbill turtle (*Eretmochelys imbricata*). Current and future habitat suitability of these species in the Gulf were projected using the modelling methods as described above. Thus, it may not include the full range of environmental and ecological factors affecting the distribution of marine turtles and marine mammals. Also, we did not predict habitat suitability for specific life stages (e.g., foraging or nesting populations), which may be more or less sensitive to environmental changes. These methodological limitations should be taken into account when interpreting our results, and the projected future distributions of charismatic species should be considered only as an indicator of their relative vulnerability to climate change. Results are discussed in S1 Appendix in the context of (a) habitat variables besides temperature and salinity that will be important in determining the populations of charismatic species’ future state under climate change; (b) where relevant, how different life history stages may be affected; (c) their migratory behavior; and (d) local stressors such as fisheries, shoreline development, dredging, and oil drilling, which are likely to represent more imminent and dangerous threats to these species’ survival than climate change.

### Vulnerability of national economies to impacts on fisheries

The Intergovernmental Panel on Climate Change (IPCC) defines ‘vulnerability’ as “the degree to which a system is susceptible to, and unable to cope with, adverse effects of climate change” [54]. Vulnerability assessments have been used in various disciplines to assess the susceptibility of natural or human systems to negative impacts as a result of human activities or natural pressures [55]. A vulnerability assessment of fisheries to climate change involves understanding the impacts of climate change on the biophysical and social components of marine ecosystems [56–59]. Here, we chose to assess the relative vulnerability of each country’s fisheries to climate change as a function of three dimensions: exposure, sensitivity and adaptive capacity [54, 56, 60–63]. Exposure is the nature and degree to which fisheries are exposed to climate change. Sensitivity usually refers to the degree to which national economies are dependent on fisheries and therefore sensitive to any changes in the sector. Adaptive capacity is the ability of a social system in the current context to anticipate, respond and adjust to changes from climate stresses, and to minimise, cope with, and recover from the consequences of climate change [64]. Adaptive capacity includes elements of social capital, human capital, and the appropriateness and effectiveness of governance structures [65].

We combined projections from ecological simulation models with indicators of the social-economic realm to examine the vulnerability of the Gulf’s national economies to the potential impacts of climate change on its marine fisheries. Note that for Saudi Arabia, Oman, and Iran, countries with fisheries in other seas beyond the Gulf, relevant variables in the vulnerability assessment were pro-rated to the proportion of total catches derived from the Gulf (S3 Table). Catches used in this analysis were “reconstructed catches” as estimated as part of the global, country-by-country research effort conducted by the *Sea Around Us* [66, 67] (see also S2 Appendix for a summary of key aspects of the methodology employed in this process as well as key findings for relevant countries).

For each of the three dimensions (Exposure [E], Sensitivity [S], and Adaptive Capacity [AC]), we selected a number of indicators, derived from separate sets of variables, to calculate the overall vulnerability index (Table 1). Most indicators were based on the criteria and assumptions listed in Allison *et al*. [56]. A comprehensive description of each indicator and its calculation is provided in S3 Appendix.

**Table 1:** Indicators and their composite variables for each dimension used to assess the vulnerability of national economies to climate change impacts on fisheries.

Indicators	Definition	Composite index	Variable^1^	Sources
**Exposure**				
Change in maximum catch potential	Projected change in maximum catch potential of each marine species exploited by each country, in the Gulf, under RCP 8.5 in 2090 relative to current status. The projected MCP of each species caught by each country was calculated by assuming the future MCP varies positively with the change in habitat suitability index.	Change in catch potential from current status under climate change	Percent change in maximum catch potential under climate change	Results from environmental niche model (ENM) and fisheries modelling
**Sensitivity**				
Employment	Importance of the marine fishery sector to local livelihoods	Number of fishers in the marine fisheries sector	Number of fishers	Teh and Sumaila [68]
		Number of fishers relative to other sectors	Proportion of economically active population (%) in the fishery sector	LABORSTA [69]
Nutritional dependence	Importance of fish as a source of nutrition and whether the nutrition provided by fisheries is sufficient to support the health of people in the country	Country’s dependence on fish as a source of protein	Fish protein as proportion (%) of all animal protein consumed	FAOSTAT [70]
		Child malnutrition	Proportion of children under five years old who are malnourished (underweight)	WHO [71]
Economic dependence	Dependence of a country’s economy on its fisheries sector	Country’s dependence on its fishery sector for revenue	Landed values as proportion (%) of total GDP	Sumaila et al. [72]; Swartz et al. [73]; The Worldbank Group [74]; Pauly and Zeller [75]
		Fisheries export value	Value of fisheries exports as proportion (%) of total exports	FAO FishStatJ [76]; UN Trade Statistics [77]; FAOSTAT [70]
		Total fisheries landings	Catch (tonnes)	Pauly and Zeller [75]
		Poverty rate	Number of people below national poverty lines (% of population)	CIA [78]; The WorldBank Group [74]; El-Khoury [79]; NationMaster [80]
Coastal protection	Importance of marine ecosystem services to minimise risks and threats from climate change	Country’s dependence on marine systems for coastal protection	Number of people living in coastal areas of elevation < 5 m (% of population)	The World Bank Group [74]
		Country’s dependence on marine systems for coastal protection	Proportion of land area of elevation <5 m	The World Bank Group [74]
**Adaptive capacity**				
Health	Average number of years that a person can expect to live	Life expectancy	Life expectancy at birth (years)	The World Bank Group [74]; UNDP [81]
Education	Education level	Adult literacy rates	Number of people over age 15 who can read and write, both sexes (% of population)	UNDP [81]
		School enrolment ratios	Number of tertiary aged people enrolled in tertiary education, both sexes (% of population)	UNDP [81]
Governance	Public institutions’ ability to conduct public affairs, manage public resources, effectively implement decisions, ensure the rule of law, improve accountability, and tackle corruption. These are generally seen as essential elements of a framework within which economies can prosper.	Political stability and absence of violence	Perceptions of the likelihood of political instability and/or politically-motivated violence (-2.5–2.5)	Kaufman *et al*. [82]; The World Bank Group [74]
		Government effectiveness	Perceptions of the quality of public services, the quality of the civil service and its independence from political pressures, the quality of policy formulation and implementation, and the credibility of the government's commitment to such policies (-2.5–2.5)	
		Regulatory quality	Perceptions of the ability of the government to formulate and implement sound policies that permit private sector development (-2.5–2.5)	
		Rule of law	Perceptions of the extent to which agents have confidence in and abide by the rules of society, the quality of contract enforcement, property rights, the police, and the courts (-2.5–2.5)	
		Voice and accountability	Extent to which a country's citizens are able to participate in selecting their government, as well as freedom of expression, freedom of association, and a free media (-2.5–2.5)	
		Control of corruption	Perceptions of the extent to which public power is exercised for private gain, including both petty and grand forms of corruption, as well as "capture" of the state by elites and private interests. (-2.5–2.5)	
Fisheries management	Resources allocated by a government to sustainably manage its fisheries	Marine Protected Areas (MPA)	Proportion of territorial sea protected (%)	IUCN and UN Environment-WCMC [83]
Size of the economy	Countries with a stronger economy may be able to divert more resources to respond and adapt to climate change	Gross Domestic Product (GDP)	Total GDP	The World Bank Group [74]
Employment alternatives	Knowledge base and skill set of the workforce	Economic complexity	The amount of knowledge embedded within an economy, as measured by the diversity and ubiquity of products in a country	MIT [84]

^1^ *For Iran*, *Oman and Saudi Arabia based on dependence from the Gulf only or pro-rated to proportion of catch derived from the Gulf only*.

The vulnerability of each country to impacts on its fisheries due to climate change was calculated by taking the unweighted average of the standardized indices for each dimension of vulnerability. We took the average, because no clear understanding of the interaction among these constituent components was available at the time analyses were undertaken. Components can be summed or multiplied, or a particular indicator within a dimension may be given more weight based on local evidence [61]. We made no *a priori* assumption about the importance of each dimension, or indicator within each dimension, in the overall sum to calculate the vulnerability of each country to climate change (i.e., V = f (E, S, AC). Thus, each of the indicators is viewed as having an equal contribution to a country’s overall vulnerability [85]. Previous studies have shown that vulnerability is robust to change in the weighting of its components and to different methods of calculations [56, 86]. A country with a high vulnerability score is assumed to have a combination of: (i) high exposure to climate change; (ii) high level of fisheries contributions to its national economy and food security; and (iii) low ability to respond and adapt to the risks posed by climate change.

## Results

### Vulnerability of marine biodiversity and fisheries to climate change

Meta-data for the 55 priority species, including habitat information, size, depth range and trophic level are presented in S2 Table. The occurrence records (global) are presented in S1 Fig. Occurrences for all of the species modelled have been recorded outside of the Gulf (i.e., the Gulf represents a subset of the overall habitat that these species inhabit). Therefore, in modelling their distribution, we used the global occurrence records to capture the full range of environmental preferences and tolerances of each species.

**Fig 2:**
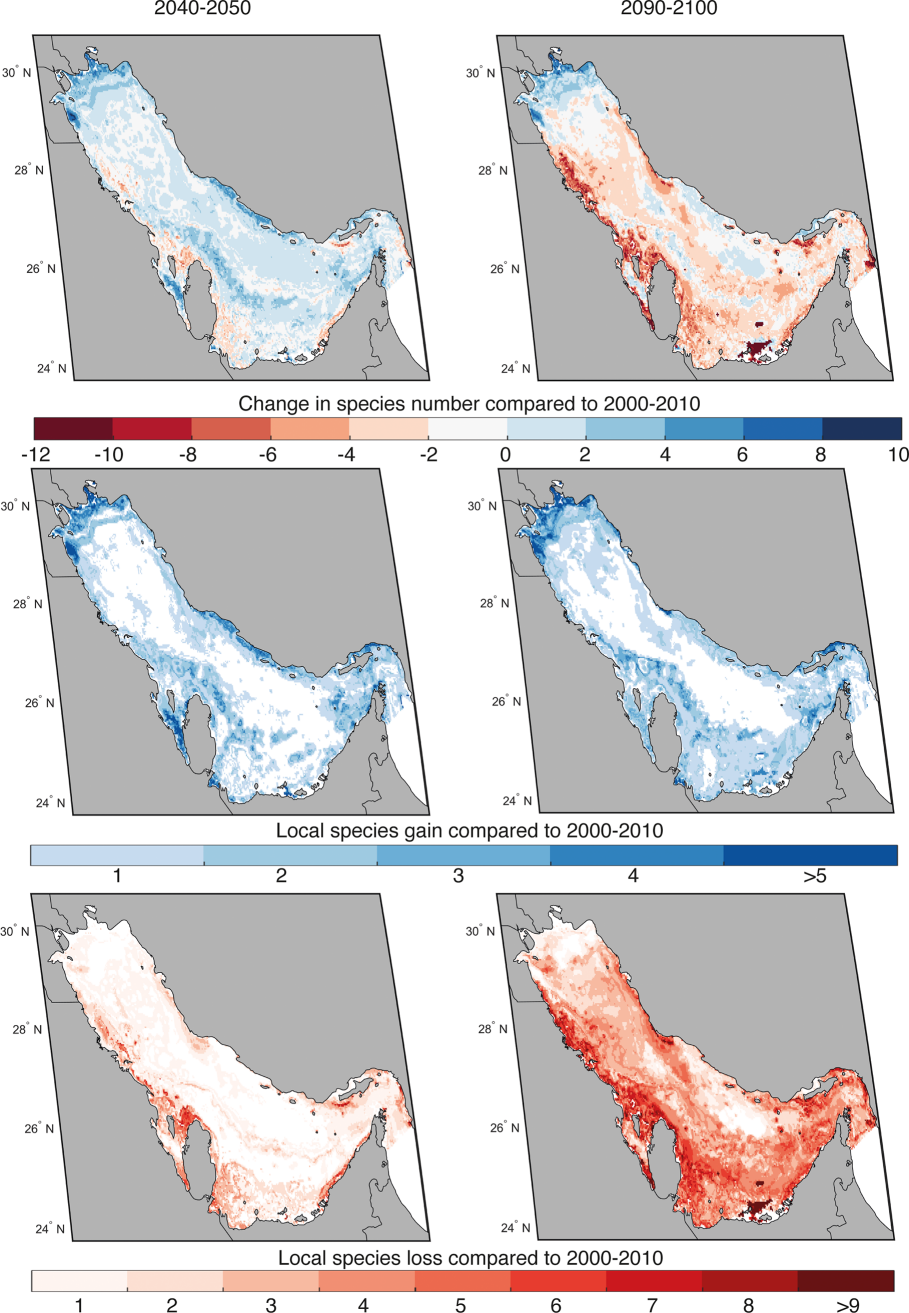
**Projected change in species number (top), species invasion (middle) and extinction (bottom) in the Gulf by 2050 (left) and 2090 (right) relative to 2010.** Results are presented for an average of the three niche models and for the RCP 8.5 scenario. The color bars represent number of species. Source: Natural Earth version 4.0.0 - http://www.naturalearthdata.com/. Figure created using MATLAB 2017b.

We predicted the current and future distributions of the 55 focal species for the period 2000–2010, 2040–2050 and 2090–2100. Projections of changes in marine species’ distributions suggest that temperature-driven climate change is expected to have severe impacts on marine biodiversity and fisheries in the Gulf. Noting that projections are possible changes in habitat suitability as estimated by the methods used herein rather than actual predicted changes in abundance, the models projected high rate of local extinction (up to 12% of initial species richness) by the end of the century relative to 2010 under the RCP 8.5 scenario (Fig 2). All results are presented as multi-model ensemble averages. Presenting the results from just one model would require scientists endorsing that specific model as possibly more valid than the others (i.e., it has fewer biases, lower variability, and therefore greater reliability). As the climate system is complex, current evidence indicates that it remains fundamentally impossible to describe all of the climate’s processes in a single model, no matter how complex the model is, with developers making choices with regards to what processes to include (and which to exclude) and how to parameterize them. As a consequence, an ensemble of several models is recommended to better account for structural and other uncertainties over time [87, 88].

**Fig 3:**
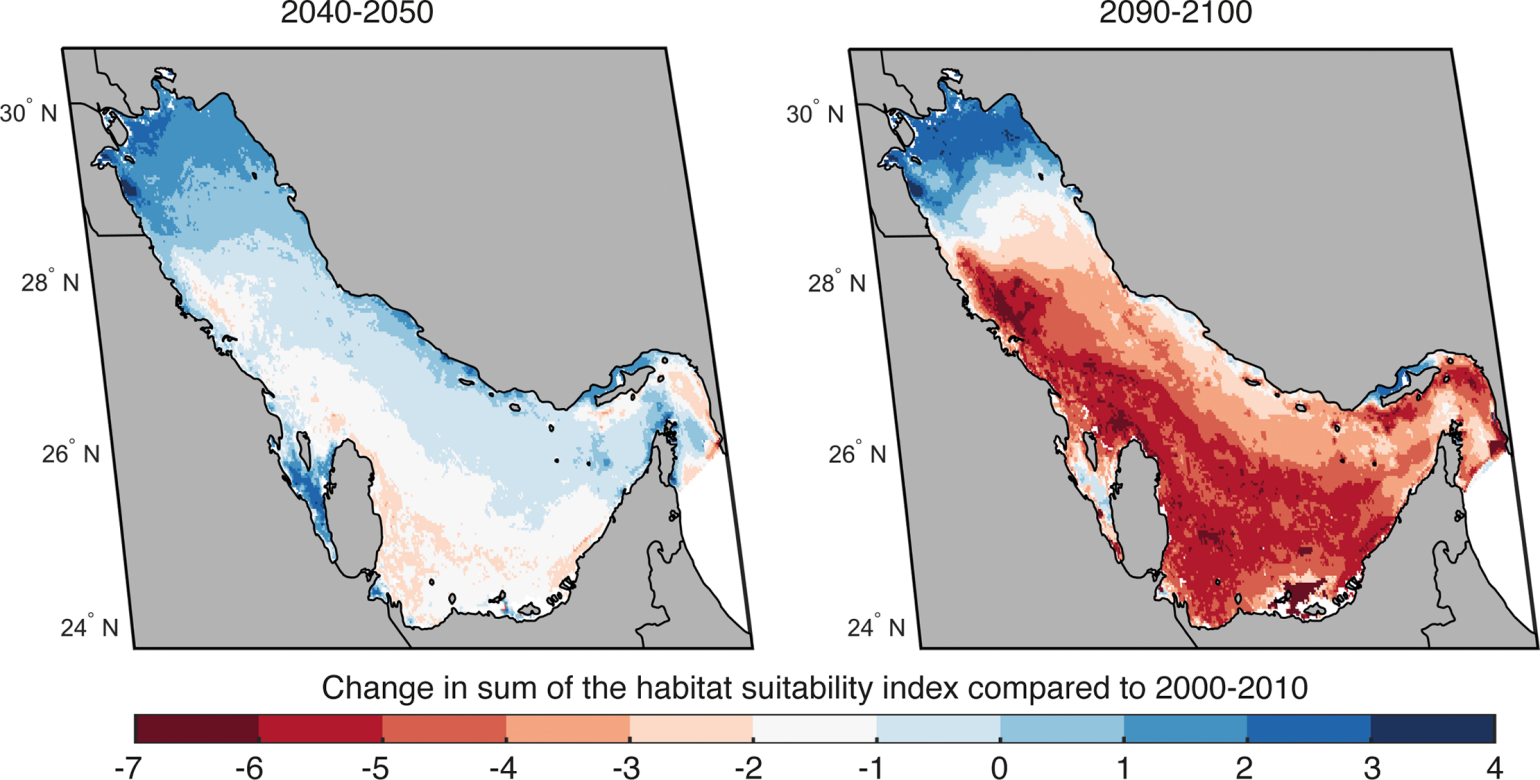
**Change in habitat suitability for focal species in the Gulf in 2050 (left) and 2090 (right) relative to 2010.** Results are presented for scenario RCP 8.5 and as averages across all three models. A decline in habitat suitability (in percentage) is shown in red, whereas increases in habitat suitability are represented in blue. Source: Natural Earth version 4.0.0 - http://www.naturalearthdata.com/. Figure created using MATLAB 2017b.

Species invasion is low (up to 5% of initial species richness). Spatially, local extinction is low to moderate in 2050, with highest species loss compared to 2010 projected along the northwest coast of Bahrain and the UAE. By 2090 species loss has risen to affect the majority of the Gulf, with highest numbers of species lost projected for the southwestern part of the Gulf, off the coast of Saudi Arabia, Bahrain, Qatar and the UAE. In contrast, species invasion by 2050 and 2090 is similar and limited to areas in the northern part of the Gulf, off the coast of Kuwait and northern Iran. This projected pattern appears to be robust, with overall congruence among all three models’ results. A drastic reduction in the total habitat biodiversity suitability (HBS) for all species by 2090 is shown in Fig 3. Climate-driven perturbations in local and regional environmental conditions will make most of the southern Gulf unsuitable for species that are presently occurring there. Projected HBS for currently recorded species in the Gulf increases in its northern part (Fig 3), potentially providing the only refuge for fauna. For example, for Spanish mackerel (*Scomberomorus commerson*), the ensemble mean habitat suitability is projected to decrease in the southern Gulf because of ocean warming. By the end of the 21st century, the only area with relatively higher habitat suitability for the species is projected to be concentrated mostly in the northern Gulf. Such a shift closely matches the gradient in sea water temperature for the Gulf predicted by Edson et al. [52].

The percent change in habitat suitability for all non-fish species in the Economic Exclusive Zones (EEZs) of Gulf countries in 2050 and 2090 under the RCP 8.5 scenario, as an average across all three models, is presented in S2 Fig.

### Vulnerability of charismatic species

**Fig 4:**
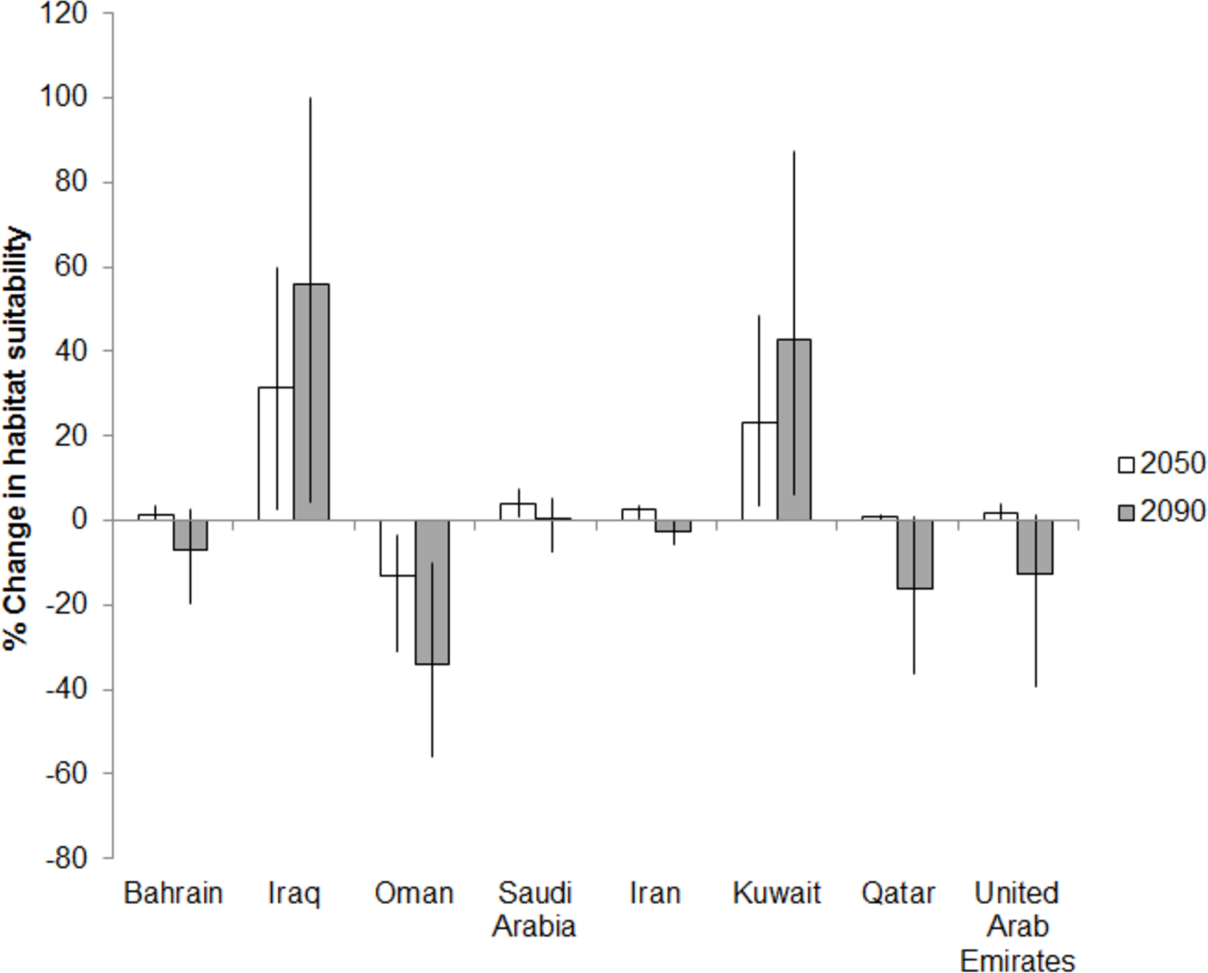
Change in habitat suitability for all charismatic species in the Economic Exclusive Zones (EEZs) of the Gulf in 2050 and 2090. Results are presented for scenario RCP 8.5 and as averages across all three models. The error bars represent the intermodal range.

Habitat suitability for all five charismatic species taken together was projected to decline most in the waters of countries on the western side of the Gulf. Areas off Oman, Bahrain and Qatar were projected to be particularly affected (Fig 4), with a 36% drop in future habitat suitability, followed by the UAE and Saudi Arabia. Waters of countries in the northern Gulf were projected to be less vulnerable. There is generally high agreement of results among the three environmental niche models.

**Fig 5:**
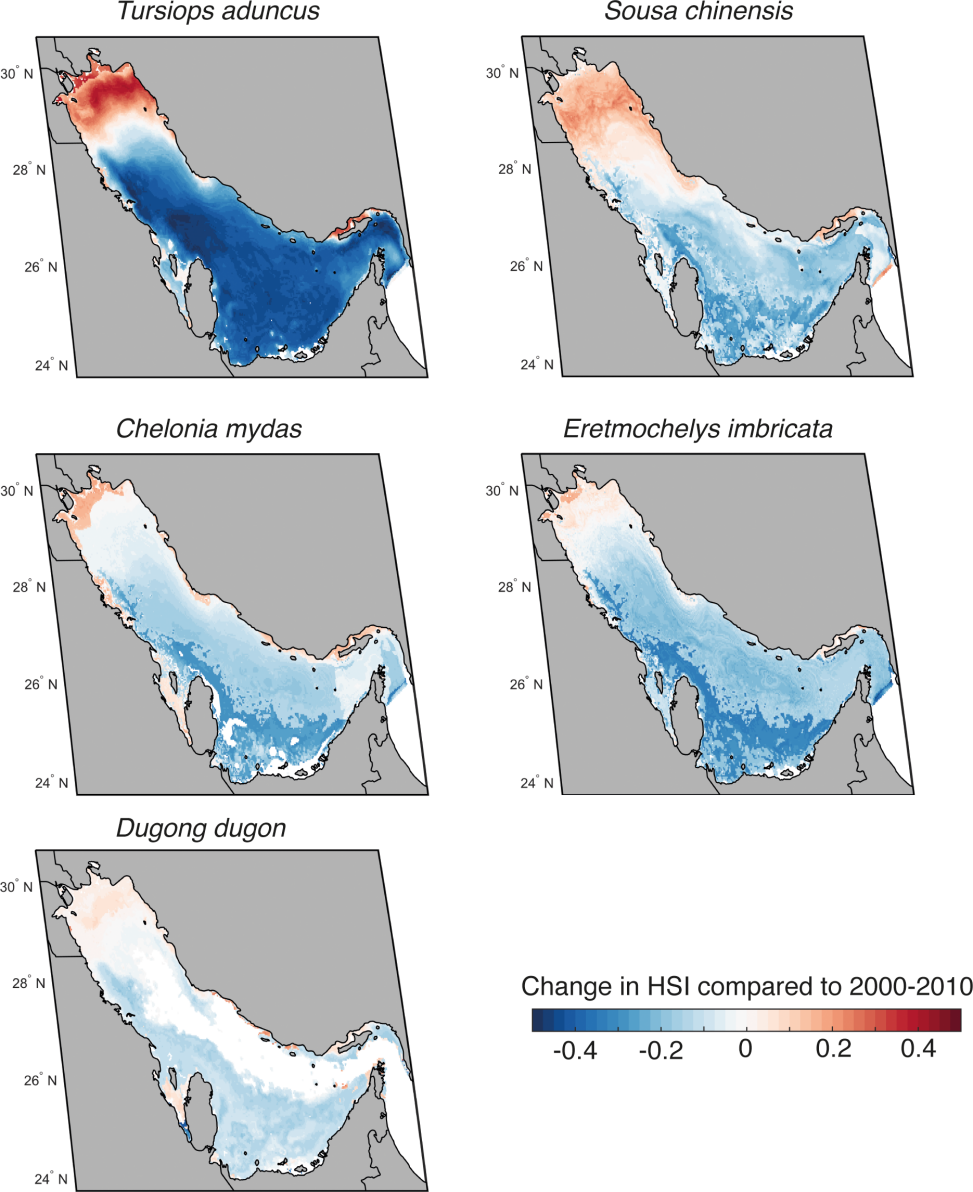
Change in habitat suitability for the 5 charismatic species in the Gulf in 2090 relative to 2010. Results are presented for scenario RCP 8.5 and as averages across all three models. The species considered include *Tursiops aduncus* (top left), *Sousa chinensis* (top right), *Chelonia mydas* (centre left), *Eretmochelys imbricata* (centre right), and *Dugong dugon* (bottom). The habitat suitability index is scaled from 0 to 1 and is the same for all species. Source: Natural Earth version 4.0.0 - http://www.naturalearthdata.com/. Figure created using MATLAB 2017b.

Our analyses also included looking at charismatic species individually (Fig 5). While models showed varying ranges of loss in habitat suitability for dugong, marine turtles, and Indo-Pacific dolphin, on the whole, future projections were largely inconclusive. For bottlenose dolphins, all three environmental niche models projected large declines in habitat suitability under climate change for most areas, with the exception of the northern region. S1 Appendix provides details on charismatic species-specific analyses and results, as well as a discussion of our findings.

Projection results for all species included in this study can also be found at https://www.ccr-group.org/copy-of-marine-inspector-resources and related links.

### Vulnerability of national economies to climate change impacts on fisheries

**Fig 6:**
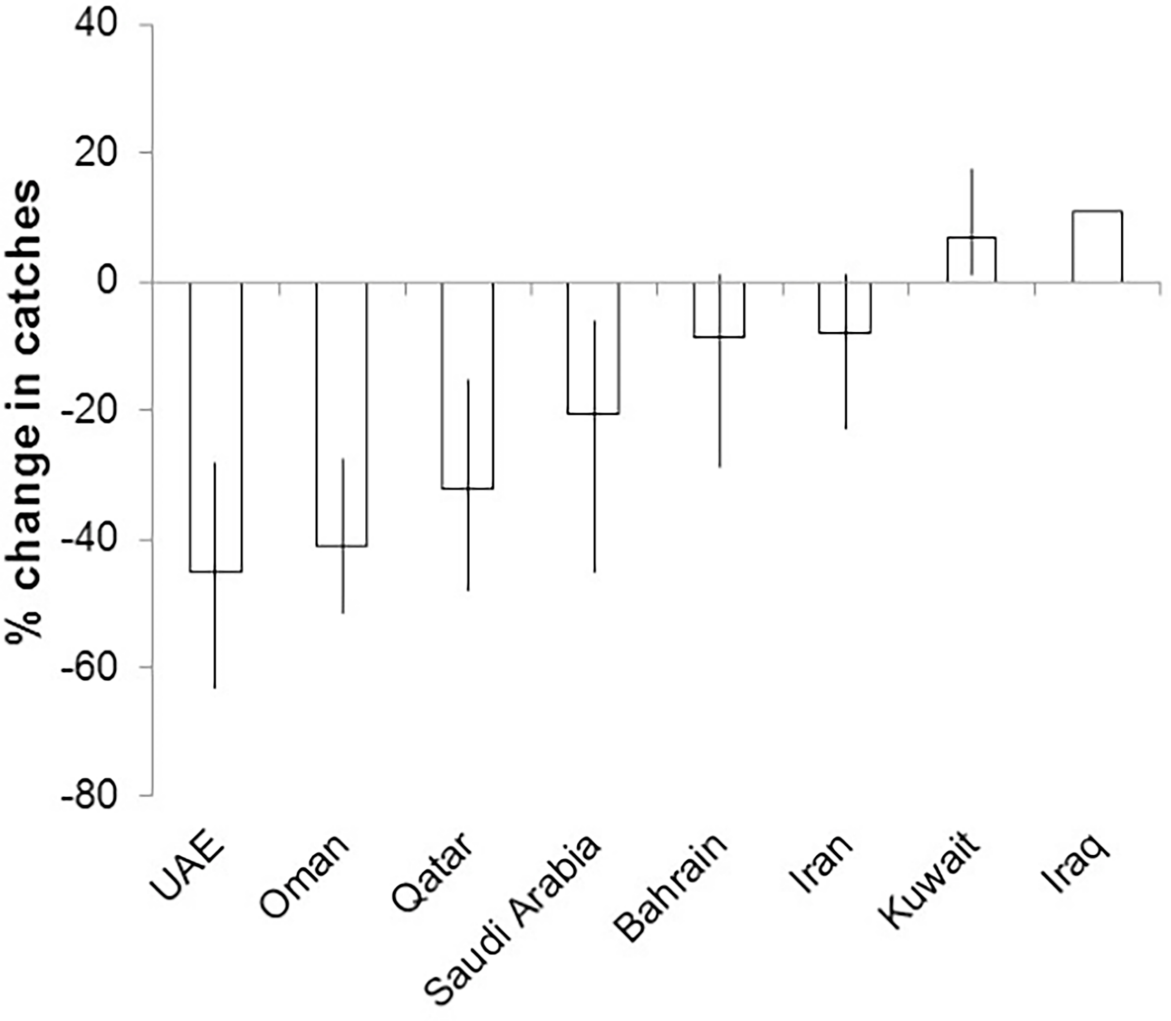
Change in catch potential in the Economic Exclusive Zones (EEZs) of the Gulf in 2090. Results are for scenario RCP 8.5 scenario as predicted by an average of the three niche models (BIOCLIM, NPPEN, and ENFA). The error bars represent inter-model range.

While projections were slightly different among models, overall catch potential declined in several countries on the western side of the Gulf (Fig 6). Qatar, Oman and the UAE were particularly affected, with a drop of more than 30% in future catch potential.

Results from the vulnerability assessment integrating changes in catch potential with socio-economic indicators showed Bahrain and Iran as most vulnerable to the impacts of climate change on fisheries (Table 2). Oman, the UAE and Iraq were labelled of “medium vulnerability”, while Kuwait and Saudi Arabia exhibited the lowest vulnerability.

**Table 2:** Relative vulnerabilities of national economies to climate change impacts on fisheries. Note that for Saudi Arabia, Oman, and Iran, countries with fisheries in other seas beyond the Gulf, relevant variables in the vulnerability assessment were pro-rated to the proportion of total catches derived from the Gulf only. Countries’ rankings are from most (1) to least vulnerable (8).

Country	Exposure	Sensitivity	Adaptive capacity*	Vulnerability Index	Rank
Bahrain^1^	0.40 (5)	0.73 (1)	0.43 (4)	**0.52**	**1**
Iran	0.39 (6)	0.48 (2)	0.68 (2)	**0.52**	**2**
Oman^2^	0.90 (2)	0.13 (7)	0.47 (3)	**0.50**	**3**
United Arab Emirates^3^	0.96 (1)	0.38 (3)	0.14 (1)	**0.50**	**4**
Iraq^4^	0.10 (8)	0.34 (4)	0.92 (8)	**0.45**	**5**
Qatar^5^	0.76 (3)	0.22 (5)	0.35 (7)	**0.44**	**6**
Saudi Arabia^6^	0.58 (4)	0.09 (8)	0.37 (6)	**0.35**	**7**
Kuwait^7^	0.16 (7)	0.18 (6)	0.47 (5)	**0.27**	**8**

^1^ Fish protein as proportion (%) of all animal protein and economic diversity values are missing for Bahrain.

^2^ Poverty rate values are missing for Oman.

^3^ Percentage of children under five who are underweight and school enrolment ratio indices are missing for the UAE.

^4^ Number of fishers in the fisheries sector; number of people involved in fisheries relative to other economic sectors and economic diversity indices are missing for Iraq.

^5^ Fish protein as proportion (%) of all animal protein and poverty rate indices are missing for Qatar.

^6^ Fish protein as proportion (%) of all animal protein and poverty rate indices are missing for Saudi Arabia.

^7^ Fisheries export value as proportion (%) of total export value and poverty rate indices are missing for Kuwait.

* The higher the value of the adaptive capacity component, the less the capacity of a country to adapt to climate change.

**Fig 7:**
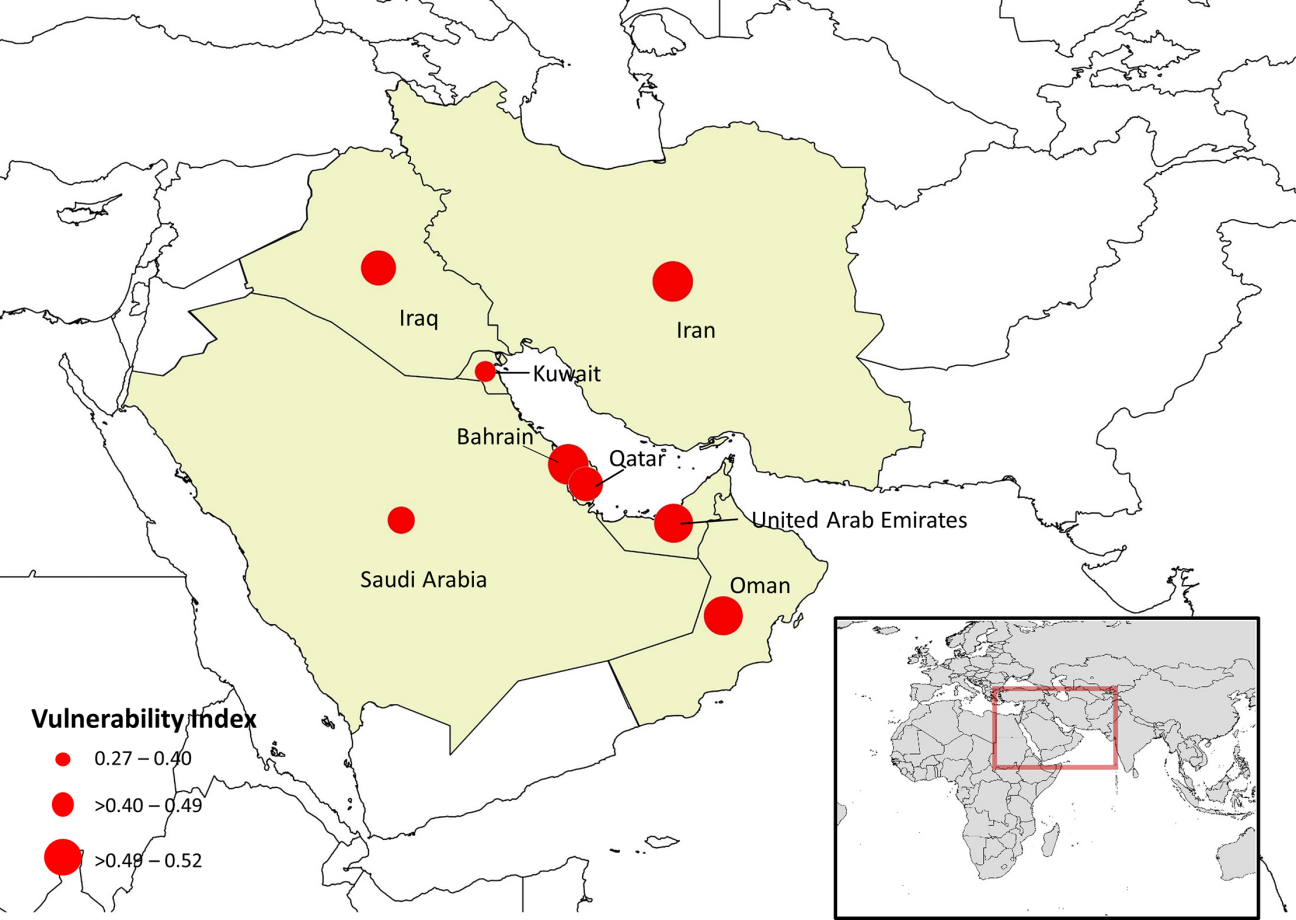
Relative vulnerability of national economies in 2090 to climate change impacts on fisheries in the Gulf. Note that for Saudi Arabia, Oman, and Iran, countries with fisheries in other seas beyond the Gulf, relevant variables in the vulnerability assessment were pro-rated to the proportion of total catches derived from the Gulf. Source: Natural Earth version 4.0.0 - http://www.naturalearthdata.com/. Map created using QGIS 2.8.2 –Wien.

For both Oman and the UAE, vulnerability is mostly tied to the country’s exposure to climate change impacts (i.e., reduced future fisheries). Although the UAE’s economy is only slightly dependent on fisheries (~0.08% of GDP), the country is highly exposed to climate change impacts, therefore yielding a relatively high overall vulnerability score (0.50). While Iraq has very low adaptive capacity, its exposure to climate change is very low, yielding a medium score for overall vulnerability (0.45). Bahrain and Iran’s fisheries ranked as the most vulnerable when combining changes in catch potential with the nation’s socio-economic framework (0.52). With a medium score for exposure and adaptive capacity, Bahrain is highly dependent on fisheries for its economy as it scored the highest for employment, as well as economic and coastal dependence on fisheries relative to other Gulf countries. While scoring relatively low for exposure, Iran ranked second highest for its sensitivity and second last for its adaptive capacity to climate change. This finding seems reasonable as Iran has the longest coastline in the Gulf, derives the highest catch, and has the least employment alternatives in the region. A map of the fisheries vulnerability index for each country is shown in Fig 7.

## Discussion

### Vulnerability of marine biodiversity and fisheries

Climate change is projected to have large impacts on marine biodiversity in the Gulf. Overall, habitat suitability for all 55 priority marine species included in this study–based on their importance to fisheries and selection by local stakeholders–is projected to undergo major declines. These findings imply that under climate change, as modelled through changes in salinity and temperature, local extinction rates are expected to increase considerably throughout the Gulf. Impacts are predicted to be particularly high along the south and southwestern coasts, where high rates of local extinction are projected by the end of the 21^st^ century.

At a global level, hydrological and biogeochemical conditions in the Gulf are considered highly specific. This area represents, for most of the environmental variables used to define the current environmental niche of species considered, the extreme end of the environmental gradient they inhabit [28, 29]. Consequently, most of the Gulf’s biodiversity can be classified into two distinct types: 1. migrating species with a high tolerance to environmental variations (i.e., euryecious) and; 2. endemic or locally-adapted species with a coarse environmental range, but highly adapted to the present environmental conditions in the Gulf (i.e., stenoecyous). The main hotspots of biodiversity are located along coasts, particularly in the south-eastern part of the Gulf and in specific regions where biogenic habitats such as coral reefs and seagrass are found (e.g., area around the Khark, Qehm or Bahrain islands and nearby protected areas such as in Heleh, Mond, Jubail or Haraye Khmair).

Although this study focused on 55 of the thousands of species occurring in the region, the general pattern of response to climate change we find is likely to be applicable to many fishes and invertebrates in the Gulf. Since most species are either highly adapted or at the edge of their environmental ranges, their sensitivity to any environmental or habitat perturbation is likely to be high. Thus, it is not surprising that projections of local species extinctions driven by temperature and salinity changes are high. Model results showed ranges of species currently occurring in the Gulf shifting poleward, from the eastern part of the Gulf to the coast of Iraq and Iran, by 2090. As species’ northern expansion/range is limited by land, the scope for these to adapt to warming through a poleward range shift is limited. Such a *cul-de-sac* effect would increase the overall rate of local extinctions in the Gulf and has been projected to occur in other semi-enclosed seas such as the Mediterranean [89]. While species may adapt by moving deeper [90], not all taxa will find suitable habitat in doing so, and the Gulf has a shallow depth limit. Biodiversity losses due to climate change are likely to be exacerbated by other direct human impacts such as pollution, eutrophication, and coastal reclamation [21, 24, 91]. Note, that our study focuses on diversity losses of species currently inhabiting the region, and does not consider possible invasion into the region by species from the Indian Ocean, that could lead to a net increase in overall biodiversity.

Results also showed that a decline in species habitat suitability translated directly into a projected decrease in maximum fisheries catch potential, particularly along the southwestern parts of the Gulf. We integrated these findings into a vulnerability assessment framework that included indicators for countries’ socio-economic sensitivity and adaptive capacity. Findings from this assessment showed that the countries that are most vulnerable to climate change impacts on fisheries were not confined to the southwestern coast, but also included Iran and Iraq. By integrating the ecological results of climate change impacts on marine biodiversity into a more comprehensive socio-economic framework, this study’s findings highlight the value of such an analysis (i) to assist national economies and societies to better anticipate, and prepare adaptive mechanisms to cope with climate change impacts so that efforts can be focused and prioritized; and (ii) to illustrate how social conditions can magnify or dampen climate change effects, in other words, climate change is best tackled by jointly addressing social and ecological issues.

### Robustness and uncertainty

#### Projecting climate change impacts on fishes and invertebrates

We evaluated the impacts of climate change based on modelled species-specific preferred ranges and drove projections using predicted shifts in temperature and salinity. For marine fishes and invertebrates, temperature is a primary climate stressor that affects their physiology, distribution and phenology [5]. However, other factors, such as oxygen concentration, acidification, and changes in ocean circulation can moderate a species response to temperature under climate change [2, 3, 90, 92, 93]. Thus, our projections are considered to be conservative because we did not account for the potential additional or synergistic effects of changes in other ocean variables. Moreover, the models used are based only on a species’ realised niche [43], with the projected distributions not taking into consideration other ecological dynamics and trophic interactions among species that are at play and that may alter our results.

The accuracy of projections is also contingent on the outputs from regional oceanographic models. We chose to use a regional oceanographic model because it provided us with high resolution projections of ocean conditions that are much more representative of the Gulf, compared to outputs from global scale Earth System Models. The environmental niche models applied in this study assume that species’ traits do not evolve as environmental conditions change, but species may well adapt to warming through genetic or transgenerational adaptations [29]. However, the extent of such adaptive responses may be limited, as suggested by the substantially lower species diversity in the Gulf relative to the adjacent Indian Ocean where conditions are not as extreme. The time frame over which they would have to evolve given the pace at which climate change is advancing may also be too short. In addition, these projections do not include how other human impacts such as changes in fishing effort may influence species’ presence and distribution as well as biodiversity patterns.

Overall, the projected patterns of change in habitat suitability for marine fishes and invertebrates should provide useful indicators of climate change impacts on their diversity and meaningfully inform the development of adaptation strategies. The magnitude of these changes however is less certain.

#### Projecting climate change impacts on charismatic species

The results of this study suggest an increase in vulnerability of charismatic species to climate change in the Gulf. For hawksbill turtles for example, habitat loss is projected to be most significant in south and southwestern parts of the Gulf. Post-nesting tracks of 90 turtles showed these areas to currently be the most important for this species [94]. Marine mammals generally have wider tolerance windows for variations in sea temperature and salinity. Therefore, projected declines in habitat suitability for dugong and dolphins may be overestimated. Overall, confidence in the projections of habitat suitability loss for charismatic species, as a result of future climate-mediated changes in temperature and salinity, is much lower than for other groups. For all charismatic species considered, changes in salinity and temperature may present stresses of relatively low concern, particularly when compared to other threats these animals face (e.g., fishing bycatch, loss of critical habitat due to pollution, eutrophication and coastal development, boat traffic, oil and gas exploration, military exercises, and biotoxins associated with red tide events). A more detailed discussion of findings is included in S1 Appendix.

#### Assessing socio-economic vulnerability to climate change impacts

Although the framework used for assessing the vulnerability of national economies to climate change impacts on fisheries is relatively comprehensive, some caveats and shortcomings in the approach remain (see S3 Appendix for details by indicator and for select variables). For example, the exposure dimension consists of one indicator (i.e., change in fisheries catch potential under climate change), while the other two dimensions are made up of several indicators. Therefore, the change in catch potential may be overrepresented in the overall vulnerability index. Moreover, because previous studies have shown results between different measures of vulnerability to be strongly correlated [86], we chose to give each indicator within a given dimension and each dimension within the overall vulnerability index equal weighting. Based on local settings, stakeholders may wish to give individual variables and/or indicators different weightings.

Projected changes in fish catches will impact the supply of fish available for local consumption (i.e., food security) and exports (i.e., income generation). The magnitude of this impact will require a detailed analysis of overlap between affected fish species and exported fish, as well as countries’ reliance on imported fish to meet local demand. While detailed considerations fall outside of the purview of this study, we suggest that national-level economic impacts are likely to be relatively minor, given that fisheries exports constitute less than 0.5% of total exports for all Gulf States. However, socio-economic impacts are likely to be comparatively greater at localized scales where there is direct and heavy reliance on fishing activities to support household incomes and where catch declines may therefore reduce the purchasing power of people to buy more nutritious food. Future studies may wish to be devoted to more comprehensive economic analyses of food supply/demand and trade, specifically addressing: the direct impact of a reduction in catches on food security (and the local socio-economy), and the indirect impacts on food security and local economies of a reduction in catches. Impacts are likely to be most severe for those economies that may need to increase imports even more because their own fisheries are suffering from climate change.

Generally, our results align well with a recent global vulnerability assessments of fisheries to climate change [59]. Beyond some differences in methodology that may explain some differences, it is important to also note that our analyses focused on the Gulf region only. Some countries with high vulnerability scores to climate change impacts (e.g., Oman, Saudi Arabia) may in reality be less exposed to climate change than results suggest based on catches obtained from, and climate change impacts on, another sea. Alternatively, given that previous studies project high impacts of climate change on fisheries in the Indian Ocean [95, 96], the vulnerability scores estimated for the Gulf may be conservative.

While it would not be practical to make generalised statements on policy and adaptation recommendations for all countries, this study shows that certain countries have comparatively higher capacity to mitigate climate change impacts on fisheries than others. For instance, the UAE appear to have reliable fisheries management, economic complexity, and a governance structure that encourages transparency, political stability, and accountability relative to other Gulf countries. These factors are all essential requirements for the design, implementation, and long-term sustainability of climate change adaptation. Policies will have to tackle the impacts of anticipated fisheries decline, to which the UAE are highly exposed to, such as reduced fish supply, unemployment in the fishing and related sectors, and the downstream effects on other sectors of the economy. Another approach is to address areas that contribute to a country’s high sensitivity ranking. For example, the physical well-being of coastal communities in Bahrain is most predisposed to the negative effects of future sea level rise. This suggests that precautionary actions should be taken to build infrastructure to make communities safe. Relevant agencies should also prepare fishing dependent households to deal with potential economic decline, through socio-economic development programmes such as financial planning education, and skills diversification.

Overall, characterization of the level of vulnerability to climate change of a fisheries-based social-ecological system is an important first step, and our assessment provides a good general indication of the potential vulnerability at the national level. Vulnerability assessments for coastal communities to climate change impacts on fisheries would require more detailed, community-specific studies. For example, participatory-based assessments could factor in the more subjective dimension of vulnerability of communities to climate stresses, helping to ensure that results can be more closely linked to effective adaptation processes on the ground [97]. This complementary methodology is also likely to have greater uptake and implementation potential. Ultimately, developing and strengthening a capacity to anticipate and act on change is fundamental [56].

### Adaptation to climate change impacts on biodiversity

Marine biodiversity was projected to be particularly vulnerable to climate change impacts along the south and southwestern coasts of the Gulf, and efforts should probably prioritise these areas. Multiple human stressors, such as habitat destruction and overfishing, are likely to exacerbate this vulnerability. Indeed, the region’s ecosystems are under the more immediate and ever-increasing pressures associated with the rapid development of economic, social and industrial activities, making the Gulf one of the highest anthropogenically impacted regions in the world [21, 22, 98]. Impacts of climate change on marine biodiversity may further affect the integrity of local ecosystems and can be moderated by reducing stresses from overfishing and destructive fishing practices; habitat degradation; pollution, including brine waste waters, domestic sewage and runoff; oil and gas exploration; land-use transformation, land reclamation, dredging activities and sedimentation. Therefore, effective implementation of ecosystem-based management that considers a much wider range of environmental and human stressors is fundamental to increasing the adaptive capacity of marine social-ecological systems to climate change. This includes strengthening the implementation and enforcement of current regulations and agreements to protect marine resources in the Gulf.

Adaptive marine conservation and management are important in uncertain future ocean ecosystems [99]. The reduced predictability of marine ecosystems due to climate change will make it more difficult to provide accurate assessments of the current and future status of marine biodiversity. Also, changing baseline oceanographic and ecological conditions may affect the effectiveness of existing conservation and management measures such as marine protected areas (MPAs). For example, by assessing the degree of future environmental change within proposed protected areas, conservation planning may be used to protect against biodiversity loss [100]. Additional MPAs to develop national and regional networks of MPAs may also increase the likelihood of effectively conserving species following climate change-induced range shifts [101, 102]. Monitoring programmes that are designed for a changing ocean and that incorporate collected data as well as adapt to analyses’ findings would allow future studies to validate (and refine) modelling projections and are thus critical to adaptive systems. Structured, integrative monitoring programmes, should include data for indicators at the pressure, state, and response levels, to allow finer-scale differentiation between climate change impacts and localised disturbances. Such programmes should be designed together with and include relevant stakeholders and be conducted at relevant spatial and temporal scales, thus allowing for appropriate management decisions to be taken and rapidly implemented. The potential for mal-adaptation and trade-offs from multiple adaptation actions should be evaluated. For example, it is expected that the expansion of desalination facilities would significantly increase average and maximum surface and bottom temperatures as well as average and maximum salinity throughout the Gulf, further exacerbating the impacts of climate change on marine species [103].

The sooner precautionary measures directly targeting fisheries effort (particularly in countries most affected by changes in catch potential) that also take into consideration future changes are adopted, the smoother the transition will be. Such considerations should involve wide-scale local stakeholder involvement at all levels to raise awareness and empower communities to aid in proposing solutions to tackle the required changes [104, 105]. Reducing compounding stresses will also help further ensure the sustainable flow of ecosystem services into the future.

## Supporting information

S1 Fig Map of occurrence records for the 55 species that were modelled in the world’s oceans, including the Gulf. Source: Natural Earth version 4.0.0 - http://www.naturalearthdata.com/. Figure created using MATLAB.
(TIF)

S2 Fig Percent change in habitat suitability forall non-fish species in the Economic Exclusive Zones (EEZs) of the Gulf in 2090. Results are presented for the RCP 8.5 scenario and as average of the three niche models (BIOCLIM, NPPEN and ENFA). The error bars represent inter-model range.
(TIF)

S1 Table Top 47 species important to fisheries in the region. Species are ordered by average catch size (tonnes).
(DOCX)

S2 Table Characteristics of all the priority marine species in the Gulf (ordered alphabetically) as obtained from FishBase [44], SeaLifeBase [45] and IUCN red list of threatened species [106]. TL–Trophic level. CR–Critically Endangered, EN–Endangered, VU–Vulnerable, NT–Near Threatened, LC–Least Concern, Data Deficient, NE–Not Evaluated.
(DOCX)

S3 Table Average annual total catch (in tonnes), Gulf catch (in tonnes) and proportion the latter represents overall by country. (DOCX)

S1 Appendix Vulnerability of charismatic species to climate change impacts. (DOCX)

S2 Appendix Fishery catch reconstruction for the Gulf. (DOCX)

S3 Appendix Vulnerability indicators: Exposure, sensitivity and adaptive capacity. (DOCX)
