# Supplementary figures and images for: Climate change impacts on marine biodiversity, fisheries and society in the Arabian Gulf
Source: PLoS One. 2018 May 2;13(5):e0194537. doi: 10.1371/journal.pone.0194537 (PMC5931652; doi:10.1371/journal.pone.0194537)

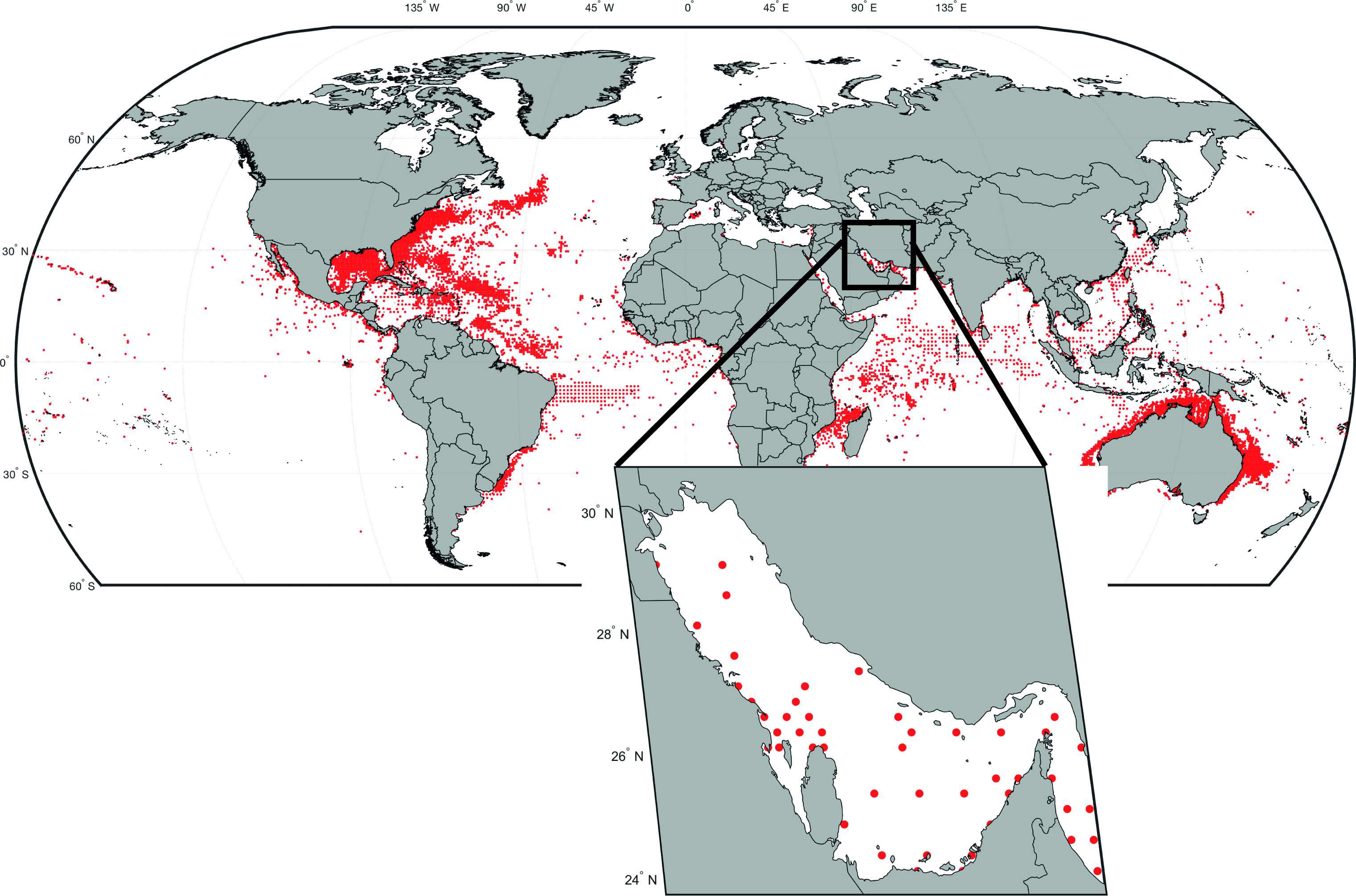

Supplement: S1 Fig — Source: Natural Earth version 4.0.0 - http://www.naturalearthdata.com/. Figure created using MATLAB. (TIF) [file pone.0194537.s001.tif]

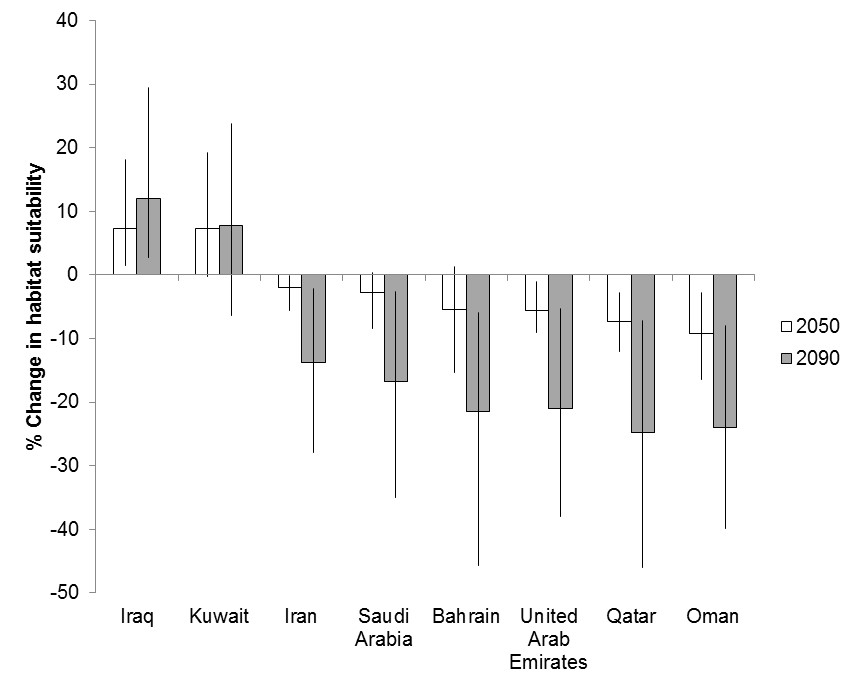

Supplement: S2 Fig — Results are presented for the RCP 8.5 scenario and as average of the three niche models (BIOCLIM, NPPEN and ENFA). The error bars represent inter-model range. (TIF) [file pone.0194537.s002.tif]
