# Supplementary material for: Climate change impacts on marine biodiversity, fisheries and society in the Arabian Gulf
Source: PLoS One. 2018 May 2;13(5):e0194537. doi: 10.1371/journal.pone.0194537 (PMC5931652; doi:10.1371/journal.pone.0194537)
Supplement: S1 Table — Species are ordered by average catch size (tonnes). (DOCX) [file pone.0194537.s003.docx]

Table S1. Top 47 species important to fisheries in the Gulf region. Species are ordered by average catch size (tonnes).

| **Family** | **Common name** | **Species** | **Average catch^1^**  **(tonnes; 2001-2010)** |
| --- | --- | --- | --- |
| Scombridae | Narrow-barred Spanish mackerel | *Scomberomorus commerson* | 30,025 |
| Clupeidae | Indian oil Sardine | *Sardinella longiceps* | 19,135 |
| Ariidae | Giant catfish | *Netuma thalassina* | 14,792 |
| Portunidae | Blue swimming crab | *Portunus segnis* | 14,575 |
| Penaeidae | Green tiger prawn | *Penaeus semisulcatus* | 14,278 |
| Carcharhinidae | Spot-tail shark | *Carcharhinus sorrah* | 11,173 |
| Carangidae | Giant trevally | *Caranx ignobilis* | 8,362 |
| Sciaenidae | Tigertooth croaker | *Otolithes ruber* | 8,041 |
| Clupeidae | Hilsa shad | *Tenualosa ilisha* | 7,066 |
| Nemipteridae | Japanese threadfin bream | *Nemipterus japonicas* | 6,984 |
| Hemiscylliidae | Grey bambooshark | *Chiloscyllium griseum* | 6,816 |
| Chirocentridae | Whitefin wolf-herring | *Chirocentrus nudus* | 5,964 |
| Mugilidae | Klunzinger's mullet | *Liza klunzingeri* | 5,609 |
| Sparidae | King solider bream | *Argyrops spinifer* | 5,533 |
| Sphyraenidae | Great barracuda | *Sphyraena barracuda* | 5,431 |
| Trichiuridae | Largehead hairtail | *Trichiurus lepturus* | 5,203 |
| Scombridae | Indo-Pacific king mackerel | *Scomberomorus guttatus* | 4,993 |
| Haemulidae | Javelin grunter | *Pomadasys kaakan* | 4,716 |
| Carangidae | Black pomfret | *Parastromateus niger* | 4,270 |
| Psettodidae | Indian halibut | *Psettodes erumei* | 4,103 |
| Haemulidae | Striped piggy | *Pomadasys stridens* | 3,815 |
| Serranidae | Orange-spotted grouper | *Epinephelus coioides* | 3,281 |
| Sparidae | Twobar seabream | *Acanthopagrus bifasciatus* | 3,253 |
| Scombridae | Bullet tuna | *Auxis rochei* | 3,166 |
| Scombridae | Indian mackerel | *Rastrelliger kanagurta* | 2,989 |
| Sciaenidae | Donkey croaker | *Pennahia anea* | 2,945 |
| Stromateidae | Silver pomfret | *Pampus argenteus* | 2,733 |
| Platycephalidae | Bartail flathead | *Platycephalus indicus* | 2,567 |
| Leiognathidae | Common ponyfish | *Leiognathus equulus* | 2,562 |
| Lethrinidae | Spangled emperor | *Lethrinus nebulosus* | 2,328 |
| Coryphaenidae | Common dolphinfish | *Coryphaena hippurus* | 2,304 |
| Lethrinidae | Pink ear emperor | *Lethrinus lentjan* | 2,164 |
| Nemipteridae | Black-streaked monocle bream | *Scolopsis taeniata* | 2,064 |
| Lutjanidae | John’s snapper | *Lutjanus johnii* | 2,033 |
| Rachycentridae | Cobia | *Rachycentron canadum* | 1,900 |
| Chanidae | Milkfish | *Chanos chanos* | 1,859 |
| Mugilidae | Bluespot mullet | *Moolgarda seheli* | 1,841 |
| Scombridae | Streaked seerfish | *Scomberomorus lineolatus* | 1,722 |
| Mugilidae | Sulphur goatfish | *Upeneus sulphureus* | 1,631 |
| Carangidae | Yellowstripe scad | *Selaroides leptolepis* | 1,628 |
| Terapontidae | Fourlined terapon | *Pelates quadrilineatus* | 1,481 |
| Gerreidae | Common silver-biddy | *Gerres oyena* | 1,457 |
| Carangidae | Bigeye scad | *Selar crumenophthalmus* | 1,457 |
| Sparidae | Haffara seabream | *Rhabdosargus haffara* | 1,425 |
| Carangidae | Golden trevally | *Gnathanodon speciosus* | 1,417 |
| Sparidae | Yellowfin seabream | *Acanthopagrus latus* | 1,400 |
| Penaeidae | Speckled shrimp | *Metapenaeus monoceros* | 1,049 |

^1^ The weighted confidence interval around the estimated total reconstructed catch, for all sectors combined (industrial, artisanal, subsistence, recreational, discards), was estimated at 22.92%
