# Supplementary material for: Climate change impacts on marine biodiversity, fisheries and society in the Arabian Gulf
Source: PLoS One. 2018 May 2;13(5):e0194537. doi: 10.1371/journal.pone.0194537 (PMC5931652; doi:10.1371/journal.pone.0194537)
Supplement: S2 Table — TL–Trophic level. CR–Critically Endangered, EN–Endangered, VU–Vulnerable, NT–Near Threatened, LC–Least Concern, Data Deficient, NE–Not Evaluated. (DOCX) [file pone.0194537.s004.docx]

**Table S2. Characteristics of all the priority marine species in the Gulf (ordered alphabetically) as obtained from FishBase [1], SeaLifeBase [2] and IUCN Red List of Threatened Species [3].** TL – Trophic level. CR – Critically Endangered, EN – Endangered, VU – Vulnerable, NT – Near Threatened, LC – Least Concern, Data Deficient, NE – Not Evaluated.

| **Name** | **Habitat** | **Habitat detail** | **Size max (mm)** | **Depth min (m)** | **Depth max (m)** | **TL** | **Information** |
| --- | --- | --- | --- | --- | --- | --- | --- |
| *Acanthopagrus bifasciatus* | demersal | benthic | 400 | 2 | 20 | 3.4 | LC |
| *Acanthopagrus latus* | demersal | benthic | 352 | 1 | 50 | 3.4 | DD |
| *Argyrops spinifer* | demersal | benthic | 700 | 1 | 450 | 4.5 | LC |
| *Auxis rochei* | pelagic | epipelagic | 350 | 1 | 1000 | 4.3 | LC |
| *Caranx ignobilis* | demersal | benthic | 1,700 | 10 | 188 | 4.2 | LC |
| *Carcharhinus sorrah* | demersal | benthic | 1,600 | 1 | 140 | 4.2 | NT |
| *Chanos chanos* | demersal | benthic | 700 | 0 | 30 | 2.4 | NE |
| *Chelonia mydas* | pelagic | epipelagic | 1,050 | 0 | 200 | 3.8 | EN |
| *Chiloscyllium griseum* | demersal | benthic | 450 | 5 | 80 | 3.7 | NT |
| *Chirocentrus nudus* | pelagic | epipelagic | 1,000 | 1 | 150 | 4.2 | NE |
| *Coryphaena hippurus* | pelagic | epipelagic | 2,100 | 1 | 85 | 4.4 | LC |
| *Crenimugil seheli* | demersal | benthic | 600 | 1 | 30 | 2.3 | NE |
| *Dugong dugon* | demersal | demersal | 1,800 | 0 | 50 | 2 | VU |
| *Epinephelus coioides* | demersal | benthic | 1,500 | 1 | 100 | 4 | NT |
| *Eretmochelys imbricata* | pelagic | epipelagic | 900 | 0 | 300 | 3 | CR |
| *Gerres oyena* | demersal | benthic | 220 | 0 | 20 | 2.7 | LC |
| *Gnathanodon speciosus* | demersal | benthic | 500 | 0 | 80 | 3.8 | LC |
| *Halodule uninervis* | benthic | benthic | - | 0 | 50 | 1 | LC |
| *Halophila ovalis* | benthic | benthic | - | 0 | 50 | 1 | LC |
| *Halophila stipulacea* | benthic | benthic | - | 0 | 50 | 1 | LC |
| *Leiognathus equulus* | demersal | benthic | 280 | 10 | 110 | 3 | LC |
| *Lethrinus lentjan* | demersal | benthic | 520 | 20 | 90 | 3.9 | LC |
| *Lethrinus nebulosus* | demersal | benthic | 870 | 10 | 75 | 3.8 | LC |
| *Liza klunzingeri* | demersal | benthic | 180 | 0 | 100 | 2.7 | NE |
| *Lutjanus johnii* | demersal | benthic | 970 | 1 | 80 | 4.2 | LC |
| *Metapenaeus monoceros* | demersal | benthic | 150 | 1 | 170 | 3 | NE |
| *Nemipterus japonicus* | demersal | benthic | 380 | 5 | 80 | 3.8 | NE |
| *Netuma thalassina* | demersal | benthic | 1,800 | 10 | 195 | 3.5 | NE |
| *Otolithes ruber* | demersal | benthic | 900 | 10 | 40 | 3.6 | NE |
| *Pampus argenteus* | demersal | benthic | 600 | 5 | 110 | 3.3 | NE |
| *Parastromateus niger* | demersal | benthic | 750 | 15 | 105 | 2.9 | NE |
| *Pelates quadrilineatus* | demersal | benthic | 300 | 1 | 21 | 3.5 | NE |
| *Penaeus semisulcatus* | demersal | benthic | 180 | 2 | 300 | 3 | NE |
| *Pennahia anea* | demersal | benthic | 300 | 1 | 60 | 4 | NE |
| *Platycephalus indicus* | demersal | benthic | 1,000 | 20 | 200 | 3.6 | DD |
| *Pomadasys kaakan* | demersal | benthic | 800 | 1 | 75 | 3.5 | NE |
| *Pomadasys stridens* | demersal | benthic | 200 | 65 | 68 | 4 | NE |
| *Portunus segnis* | demersal | benthic | 200 | 1 | 50 | 3.1 | NE |
| *Psettodes erumei* | demersal | benthic | 640 | 1 | 100 | 4.4 | NE |
| *Rachycentron canadum* | pelagic | epipelagic | 2,000 | 0 | 1200 | 4 | LC |
| *Rastrelliger kanagurta* | pelagic | epipelagic | 380 | 20 | 90 | 3.2 | DD |
| *Rhabdosargus haffara* | demersal | benthic | 350 | 10 | 100 | 3.5 | LC |
| *Sardinella longiceps* | pelagic | epipelagic | 200 | 20 | 200 | 2.5 | LC |
| *Scolopsis taeniata* | demersal | benthic | 380 | 20 | 50 | 3.6 | NE |
| *Scomberomorus commerson* | pelagic | epipelagic | 850 | 10 | 70 | 4.5 | NT |
| *Scomberomorus guttatus* | pelagic | epipelagic | 760 | 15 | 200 | 4.3 | DD |
| *Scomberomorus lineolatus* | pelagic | epipelagic | 980 | 1 | 9999 | 4.5 | LC |
| *Selar crumenophthalmus* | pelagic | epipelagic | 700 | 1 | 170 | 3.8 | LC |
| *Selaroides leptolepis* | pelagic | epipelagic | 150 | 1 | 25 | 3.8 | LC |
| *Sousa chinensis* | pelagic | epipelagic | 3,200 | 0 | 500 | 4 | NT |
| *Sphyraena barracuda* | demersal | benthic | 2,000 | 1 | 100 | 4.5 | LC |
| *Tenualosa ilisha* | pelagic | epipelagic | 600 | 10 | 200 | 2.9 | LC |
| *Trichiurus lepturus* | demersal | benthic | 2,340 | 1 | 589 | 4.4 | LC |
| *Tursiops aduncus* | pelagic | epipelagic | 2,000 | 0 | 2000 | 4 | DD |
| *Upeneus sulphureus* | demersal | benthic | 103 | 10 | 90 | 3.1 | LC |

**References**

1. Froese R, Pauly D. Fishbase [Internet]. 2016 [cited 20 March 2016]. Available from: [www.fishbase.org](http://www.fishbase.org).

2. Palomares MLD, Pauly D. SeaLifeBase [Internet]. 2016 [cited 20 March 2016]. Available from: [www.sealifebase.org](http://www.sealifebase.org).

3. The IUCN Red List of Threatened Species. Version 2017-1 2017 [cited Accessed on 1 September 2017]. Available from: [www.iucnredlist.org](http://www.iucnredlist.org).
