# Supplementary material for: Climate change impacts on marine biodiversity, fisheries and society in the Arabian Gulf
Source: PLoS One. 2018 May 2;13(5):e0194537. doi: 10.1371/journal.pone.0194537 (PMC5931652; doi:10.1371/journal.pone.0194537)
Supplement: S3 Table — (DOCX) [file pone.0194537.s005.docx]

Table S3. Average annual total catch (in tonnes), Gulf catch (in tonnes) and proportion the latter represents overall by country.

| **Country** | **Average annual total catch (tonnes)** | **Gulf Catch (tonnes)** | **Proportion in  Gulf** |
| --- | --- | --- | --- |
| Bahrain | 38,599.71 | 38,599.71 | 1 |
| Iran | 162,120.10 | 121,802.61 | 0.75 |
| Iraq | 12,868.92 | 12,868.92 | 1 |
| Kuwait | 38,692.13 | 38,692.13 | 1 |
| Oman | 90,088.77 | 3,688.36 | 0.04 |
| Qatar | 12,072.33 | 12,072.33 | 1 |
| Saudi Arabia | 39,501.83 | 19,872.86 | 0.50 |
| United Arab Emirates | 39,508.20 | 39,508.20 | 1 |
