# Supplementary material for: Climate change impacts on marine biodiversity, fisheries and society in the Arabian Gulf
Source: PLoS One. 2018 May 2;13(5):e0194537. doi: 10.1371/journal.pone.0194537 (PMC5931652; doi:10.1371/journal.pone.0194537)
Supplement: S1 Appendix — (DOCX) [file pone.0194537.s006.docx]

### S1 Appendix

#### **Vulnerability of charismatic species**

### Dugong (*Dugong dugon*)

The Gulf is currently the major remaining habitat for dugong [1], after Northern Australia [2]. Projections from BIOCLIM and NPPEN showed that the Gulf would become less hospitable to dugongs, particularly around the southwestern region such as the waters around Bahrain. However, habitat suitability predicted by the ENFA model, the least conservative among the three models, showed essentially no loss of habitat suitability for dugongs under climate change. The projection results as an average across all three models used for dugong in the EEZs of the Arabian Gulf are displayed in Fig 1. The percent change in habitat suitability as averaged across all three models in 2050 and 2090 under the RCP 8.5 scenario is presented in Fig 2.

| 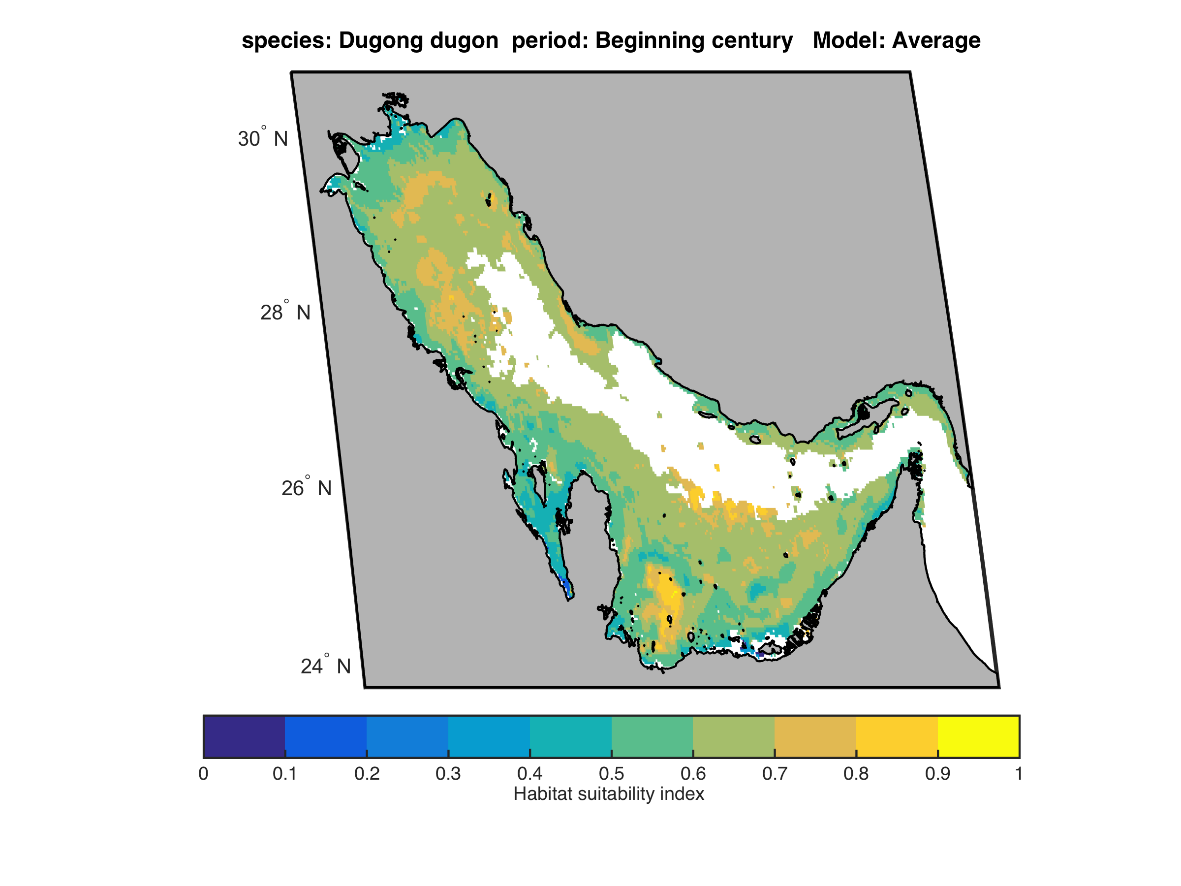 |
| --- |
| 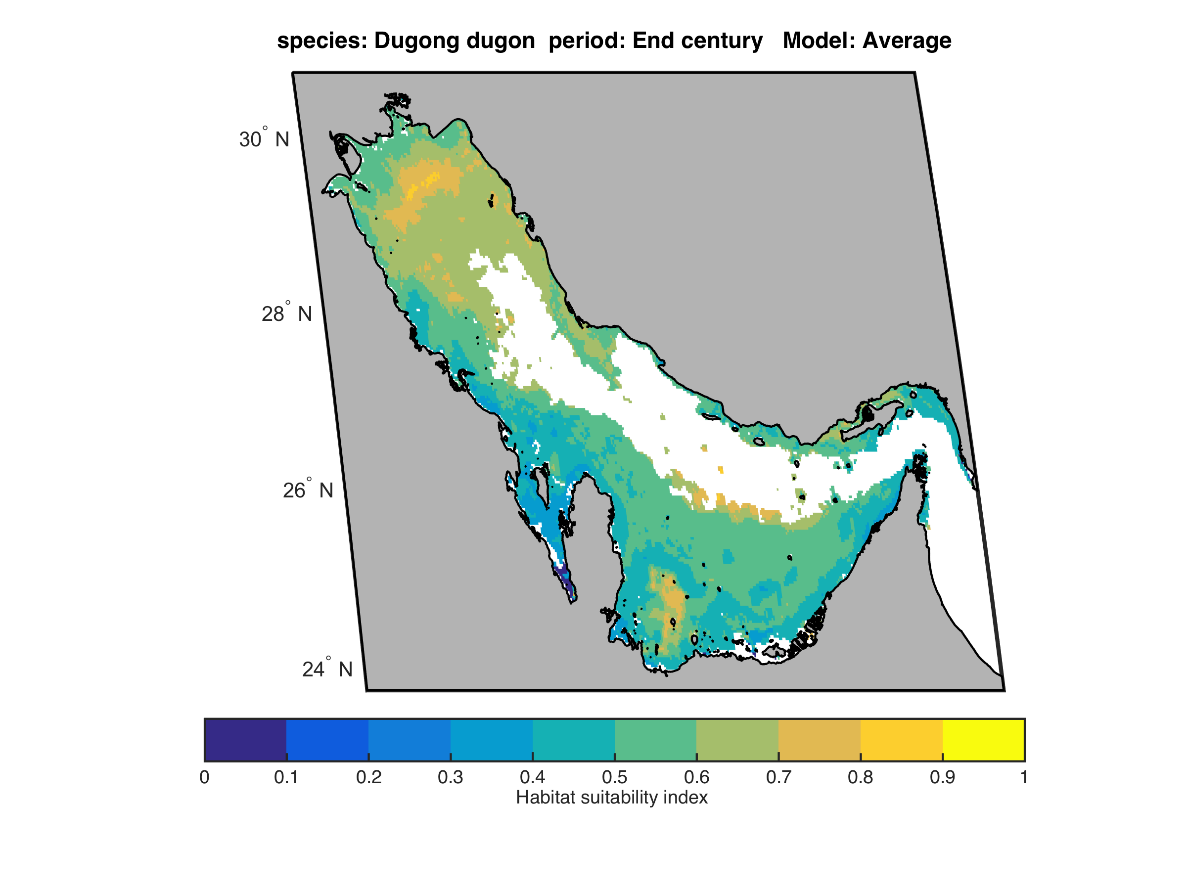 |

Fig 1. Projected 2010 (average of 2000-2010; top panel) and 2090 (average of 2090-2100; bottom panel) distributions and habitat suitability of dugong in the Gulf. Results are presented for RCP 8.5 as an average of the three niche models used in this study (NPPEN, ENFA and BIOCLIM). Habitat suitability is scaled from 0 to 1, with 0 being not suitable and 1 most suitable. White areas indicate a probability of occurrence for the species equal to zero, therefore it is equivalent to loss of habitat.


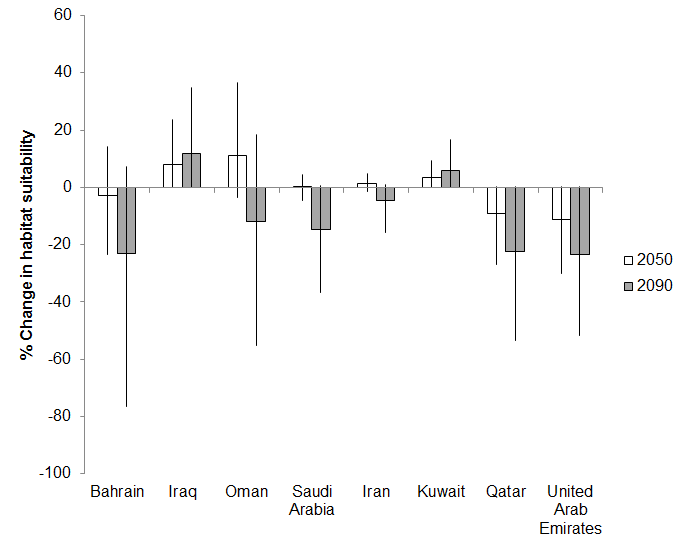


Fig 2. Percent change in habitat suitability for dugongs in the Economic Exclusive Zones (EEZs) of the Gulf in 2050 and 2090. Results are for scenario RCP 8.5 and represent an average across all three models. The error bars denote inter-model range.

Note that all results show future modelled habitat suitability in the region according to projected changes in temperature and salinity relative to the preferred environmental niche of the marine mammal itself. Consequently, these projections do not take into account the fact that dugongs rely on seagrass for almost their entire diet, and the likely resultant changes in dugongs’ habitat suitability based on projected changes to seagrass distribution. It is expected that in the future dugongs will be more at risk due to threats such as rapid coastal development, incidental bycatch in gillnet and driftnet fisheries, and oil spills [1] than climate change.

### Hawksbill (*Eretmochelys imbricata*) and green turtles (*Chelonia mydas*)

Projections from BIOCLIM and NPPEN, based on estimates of future temperature and salinity relative to hawksbill and green turtles’ environmental niches, showed a loss of habitat suitability for both species around the southwestern parts of the Gulf and near the Strait of Hormuz, with the latter model also showing loss of habitat in the northern parts of the Gulf. Findings from the ENFA projections agree with these results, but the loss of suitable habitat in the south and southwestern Gulf were more severe. The projected patterns of changes in habitat suitability are similar between green and hawksbill turtles, except that NPPEN projects a more substantial habitat loss for green turtles along the Gulf coast. The projection results as an average across all three models used for hawksbill and green turtles in the EEZs of the Gulf are displayed in Fig 3 and Fig 4 respectively. A graph showing an average across all three models of percent change in habitat suitability for green turtles in the EEZs of the Gulf in 2050 and 2090 under the RCP 8.5 scenario is included in Fig 5.

| 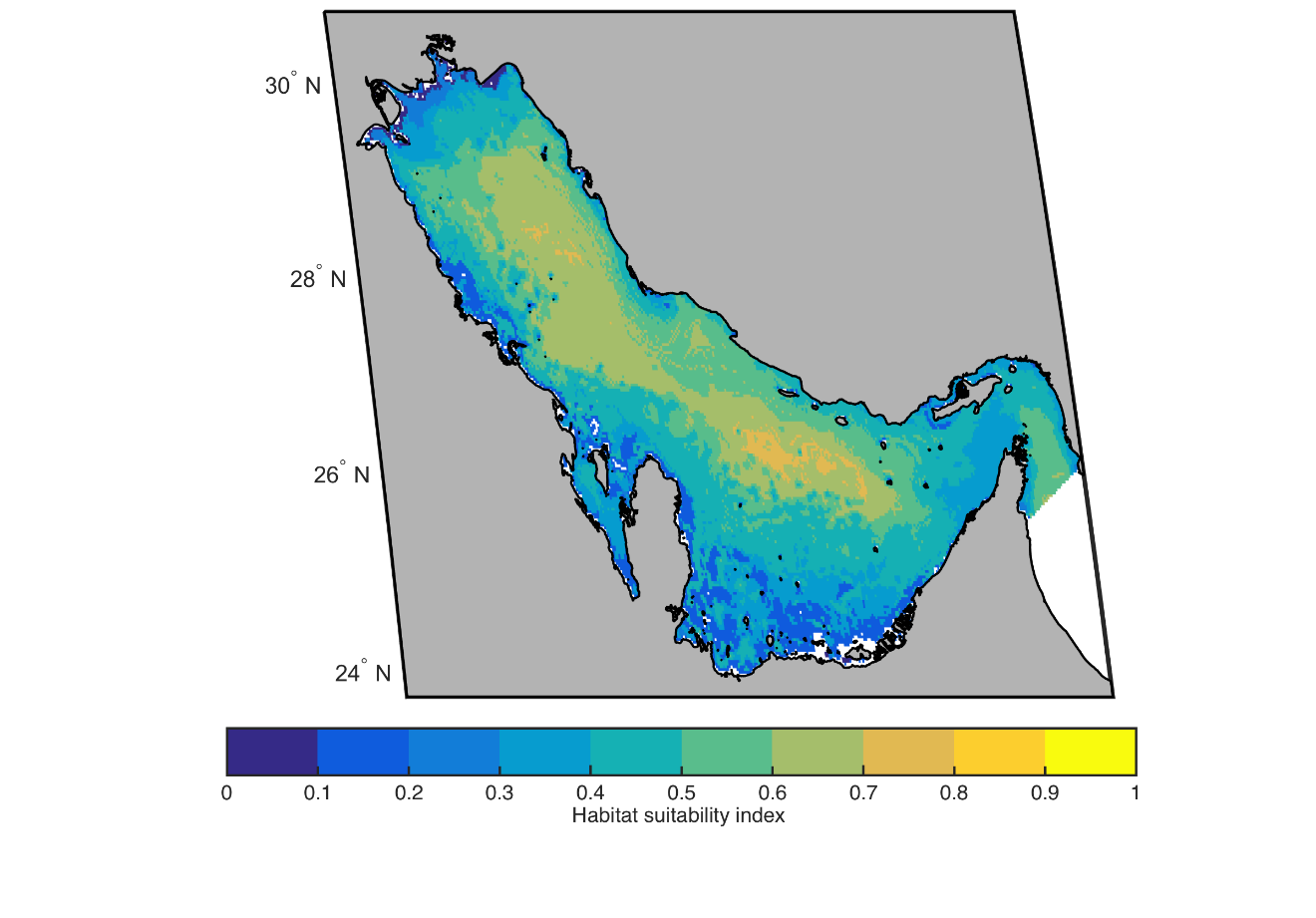 |
| --- |
| 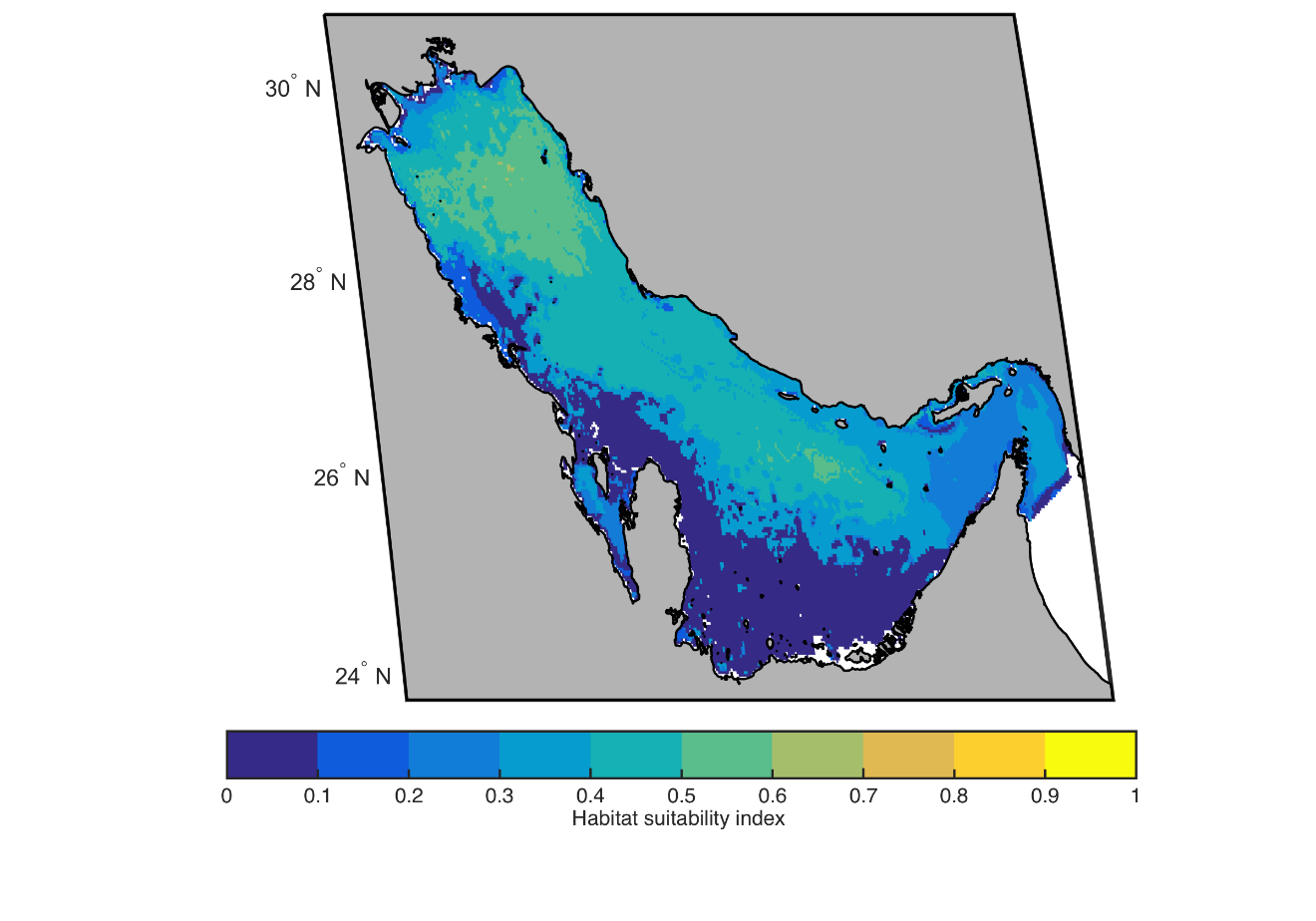 |

Fig 3. Projected 2010 (top panel) and 2090 (bottom panel) distributions and habitat suitability for hawksbill turtles in the Gulf. Results are presented for RCP 8.5 as an average of the three niche models used in this study, NPPEN, ENFA and BIOCLIM. Habitat suitability is scaled from 0 to 1, with 0 being not suitable and 1 most suitable. White areas indicate a probability of occurrence for the species equal to zero, therefore it is equivalent to loss of habitat.

| 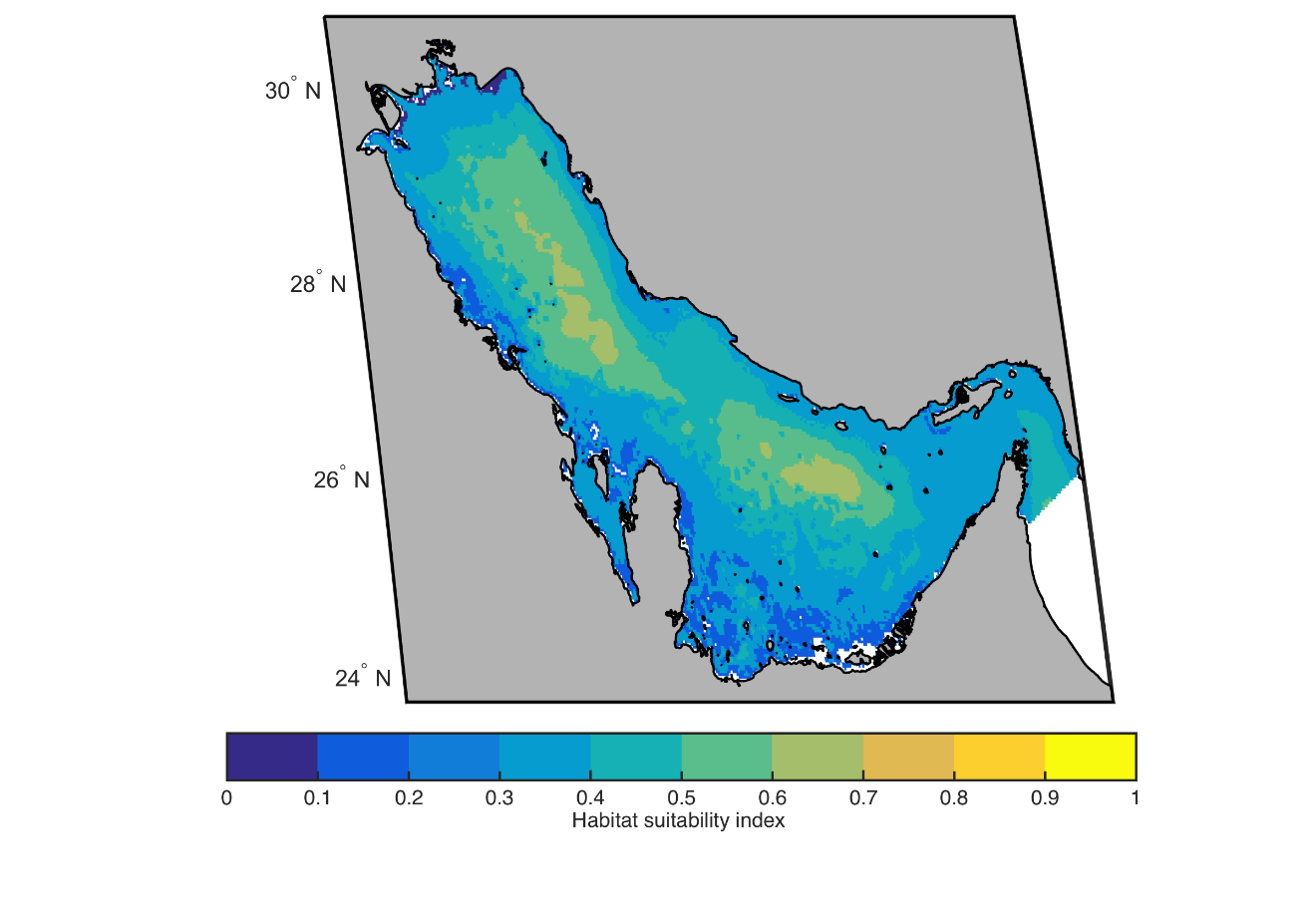 |
| --- |
| 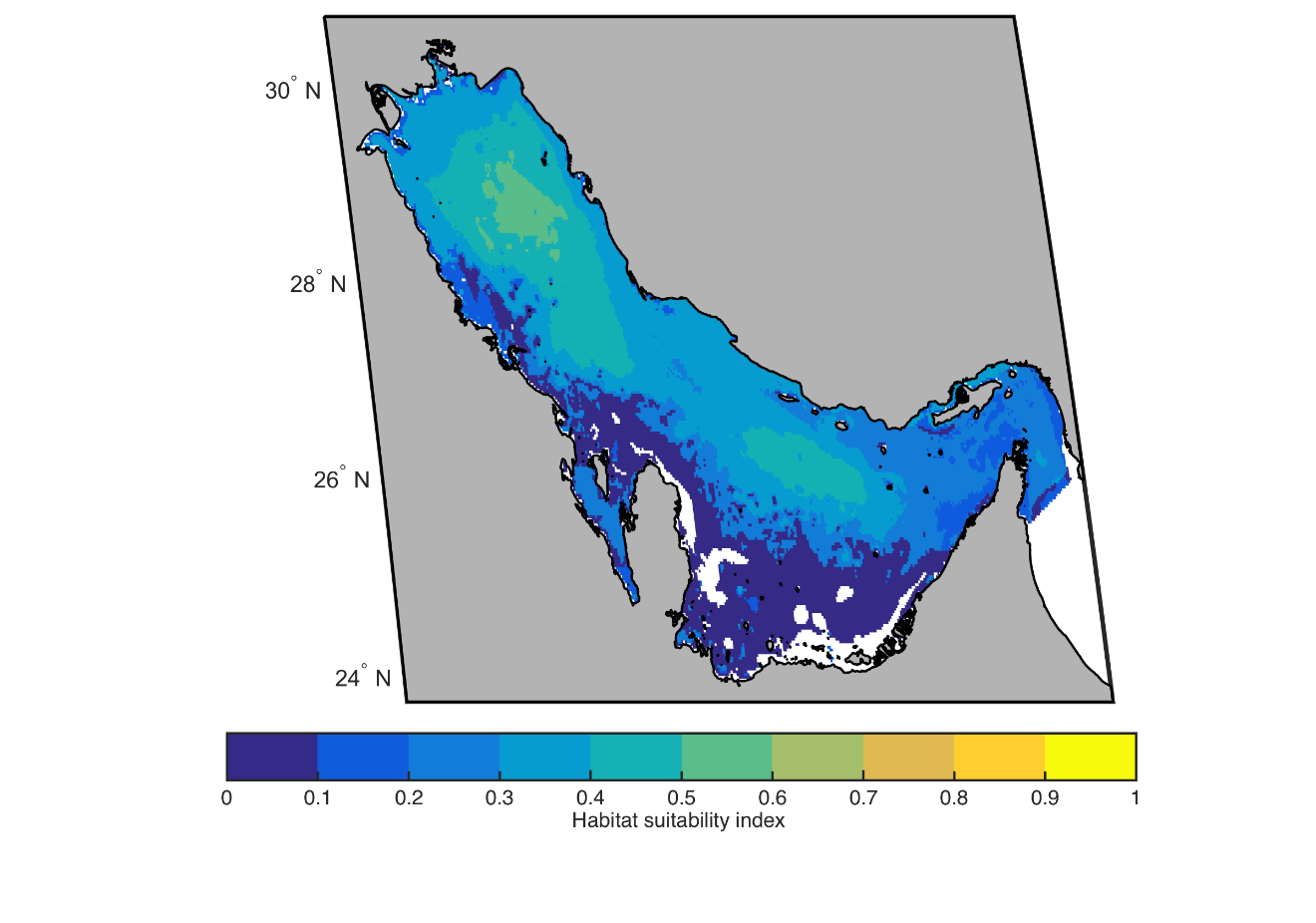 |

Fig 4. Projected 2010 (top panel) and 2090 (bottom panel) distributions and habitat suitability for green turtles in the Gulf. Results are presented for RCP 8.5 as an average of the three niche models used in this study, NPPEN, ENFA and BIOCLIM. Habitat suitability is scaled from 0 to 1, with 0 being not suitable and 1 most suitable. White areas indicate a probability of occurrence for the species equal to zero, therefore it is equivalent to loss of habitat.


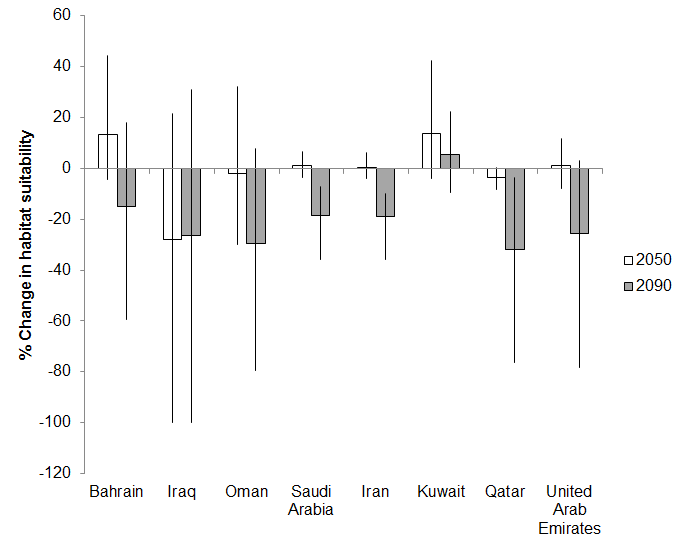


Fig 5. Percent change in habitat suitability for green turtles in the Economic Exclusive Zones (EEZs) of the Gulf in 2050 and 2090 Results are for scenario RCP 8.5 and represent an average across all three models. The error bars denote inter-model range.

### Indo Pacific humpback dolphin (*Sousa chinensis*)

The BIOCLIM model projections showed loss of habitat suitability for Indo Pacific humpback dolphins particularly around the southwestern parts of the Gulf. Projections based on NPPEN were similar, expanding to Bahrain and Qatar. ENFA model runs demonstrated uniform loss of habitat suitability throughout the lower three quarters of the Gulf. The projection results as an average across all three models used for Indo Pacific dolphins in the EEZs of the Gulf are displayed in Fig 6. A graph showing an average across all three models of percent change in habitat suitability in 2050, 2090 under the RCP 8.5 scenario is included in Fig 7.

| 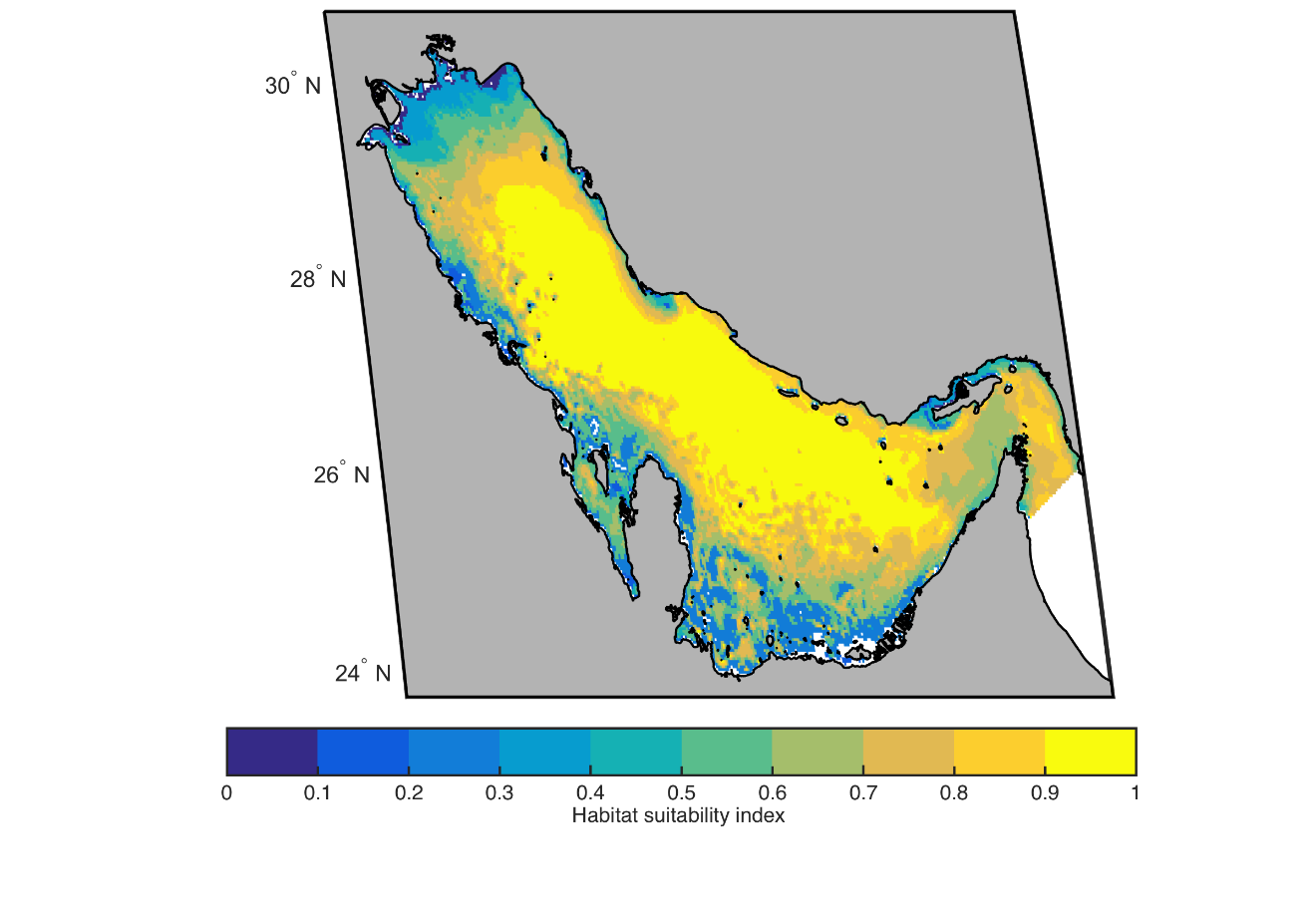 |
| --- |
| 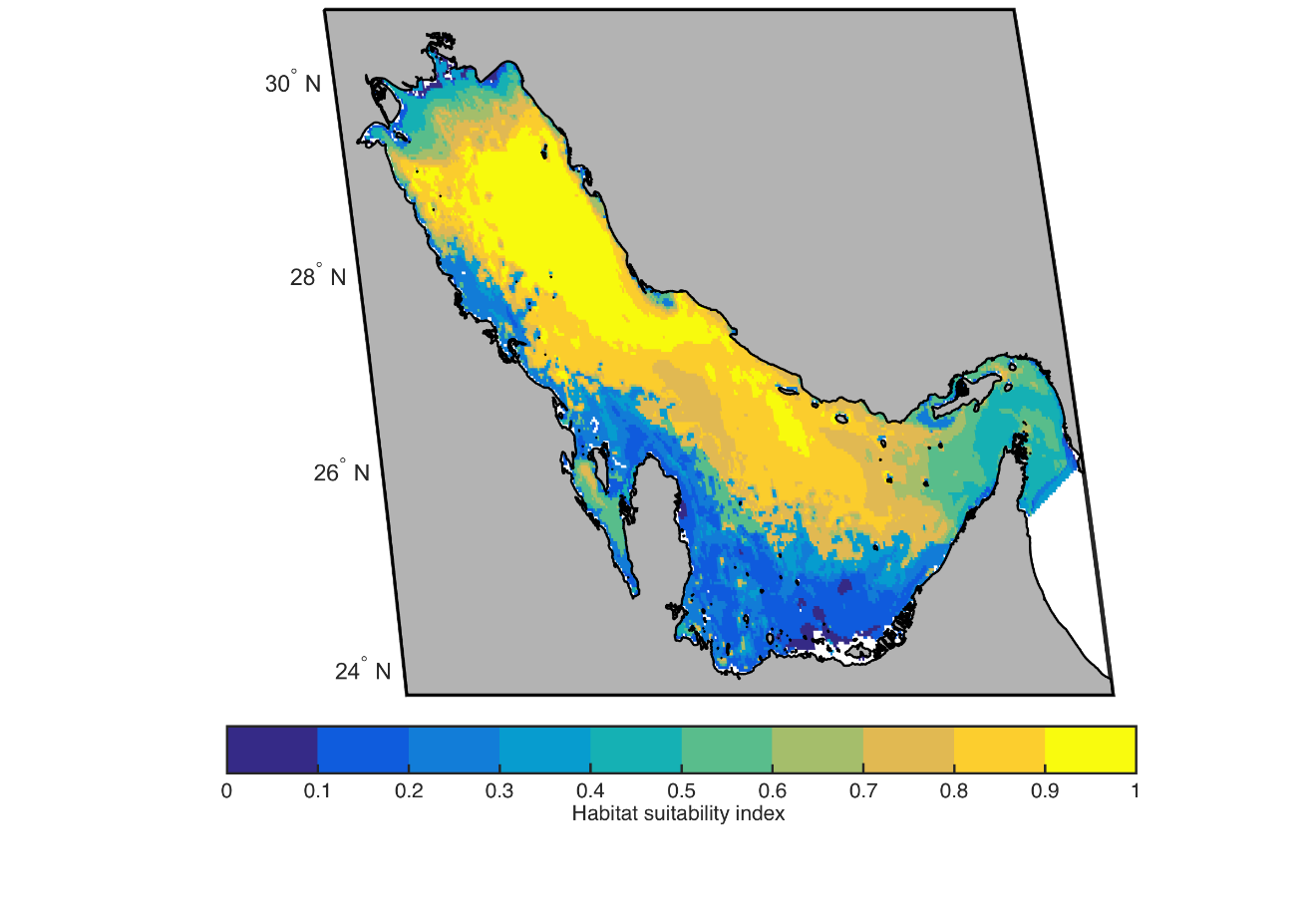 |

Fig 6. Projected 2010 (top panel) and 2090 (bottom panel) distributions and habitat suitability for Indo Pacific dolphins in the Gulf. Results are presented for RCP 8.5 as an average of the three niche models used in this study, NPPEN, ENFA and BIOCLIM. Habitat suitability is scaled from 0 to 1, with 0 being not suitable and 1 most suitable. White areas indicate a probability of occurrence for the species equal to zero, therefore it is equivalent to loss of habitat.


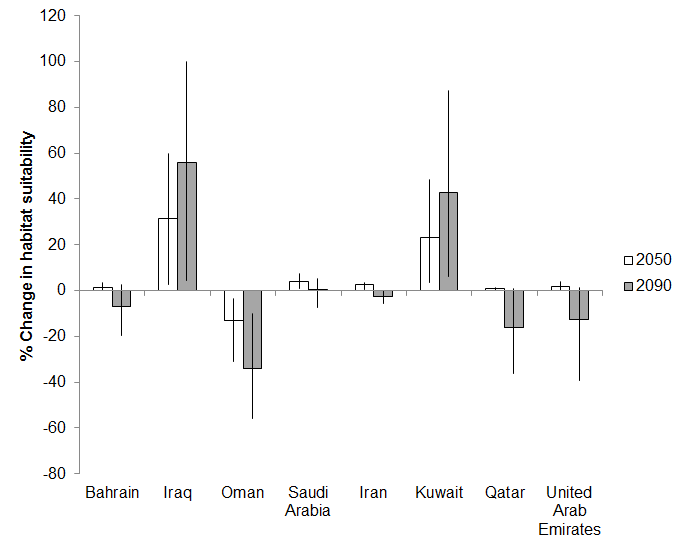


Fig 7. Percent change in habitat suitability for Indo Pacific dolphins in the Economic Exclusive Zones (EEZs) of the Gulf in 2050 and 2090. Results are for scenario RCP 8.5 and represent an average across all three models. The error bars denote inter-model range.

### Bottlenose dolphin (*Tursiops aduncus*)

All three environmental niche models projected large declines in habitat suitability of bottlenose dolphin for most areas in the Gulf, with the exception of the northern region, under climate change (Fig 8). The pattern of changes is largely consistent among results from the three models. However, projected changes in habitat suitability from BIOCLIM by 2090 relative to 2010 under the RCP 8.5 scenario are more conservative, with a smaller decline in habitat suitability relative to projections from the other two models.

| 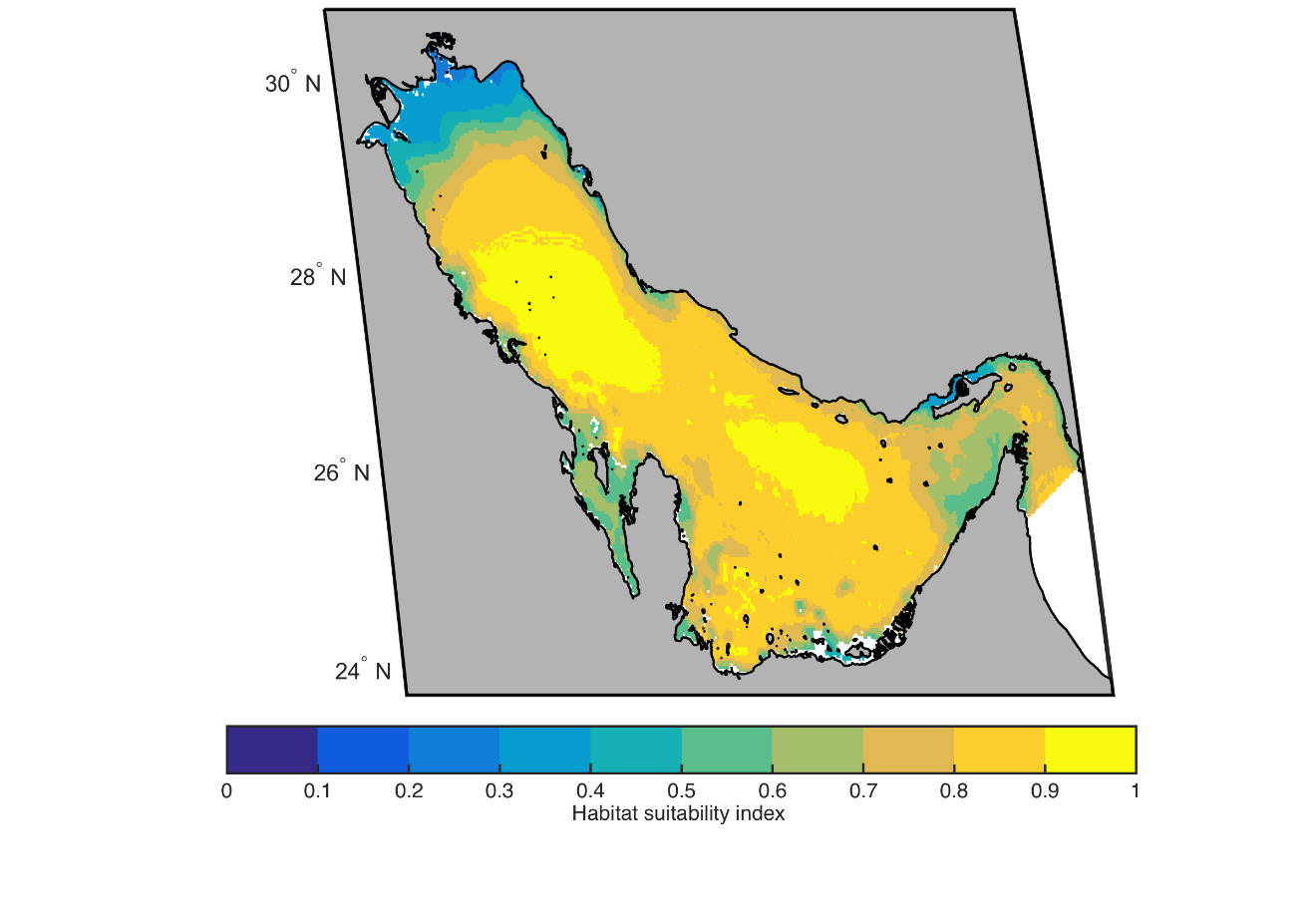 |
| --- |
| 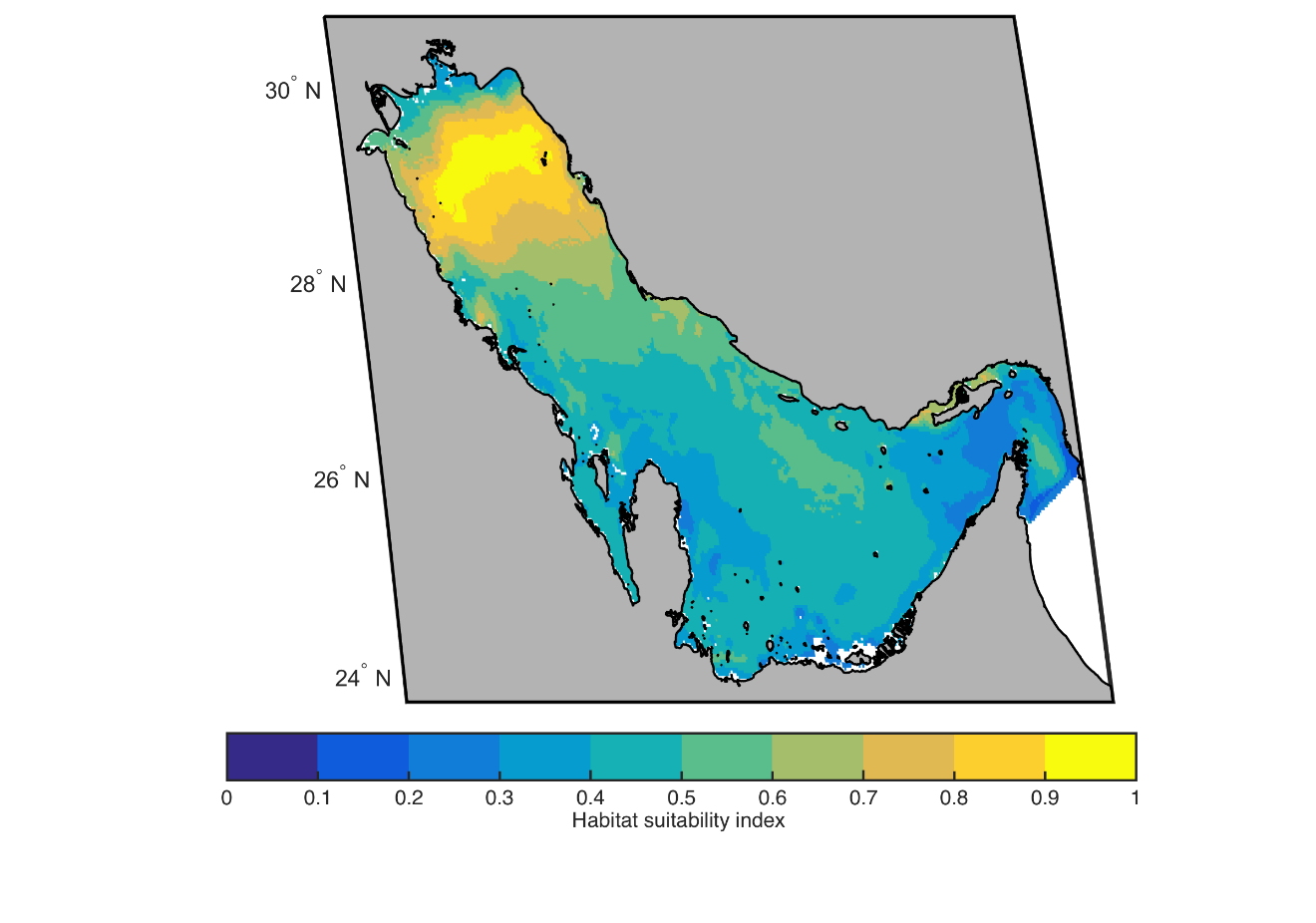 |

Fig 8. Projected 2010 (top panel) and 2090 (bottom panel) distributions and habitat suitability for bottlenose dolphins in the Gulf. Results are presented for RCP 8.5 as an average of the three niche models used in this study, NPPEN, ENFA and BIOCLIM. Habitat suitability is scaled from 0 to 1, with 0 being not suitable and 1 most suitable. White areas indicate a probability of occurrence for the species equal to zero, therefore it is equivalent to loss of habitat.

### Projecting climate change impacts on charismatic species

The results of this study suggest an increase in vulnerability of charismatic species to climate change in the Gulf. For hawksbill turtles for example, model projections based on changes in temperature and salinity relative to the species’ environmental niche predict that habitat loss would be most significant in south and southwestern parts of the Gulf. Post-nesting tracks of 90 turtles showed these areas to currently be the most important for this species in the Arabian region [3]. Marine mammals generally have wider tolerance windows for variations in sea temperature and salinity. Therefore, projected declines in habitat suitability for dugongs and dolphins may be overestimated. Overall, confidence in the projections of habitat suitability loss for charismatic species, as a result of future climate-mediated changes in temperature and salinity, is much lower than for other groups.

The approach utilized here did not include behavioural or other species characteristics that may make these species vulnerable to climate change stresses. For instance, sea turtles have a complex life history. Females lay eggs on their natal beaches, and hatchlings enter the ocean where they grow into juveniles on the high seas before recruiting to neritic habitats several years later. As adults, sea turtles are highly migratory, travelling between foraging and nesting grounds that can sometimes be oceans apart (>3000 km). Some of these life stages may be more vulnerable to increasing temperatures than others, with warming for example found to result in a shift in sex ratios towards females at many rookeries [4][4 but see] (but see Pilcher et al. [5]) and a decline in the fitness of hatchlings.

In the case of sea turtles in the Gulf, overall, our modelling results show that changes in salinity and temperature may present stresses of relatively low concern to the sea turtles themselves [see also 5], particularly when compared to other threats they face in the region [6]. Nonetheless, a recent study in the Gulf showed that a number of hawksbills travelling between nesting and foraging grounds when water temperatures are elevated undertook summer migration loops generally moving in a northeasterly direction toward deeper water, swam at greater speeds, and had trajectories that were significantly inversely correlated with temperature [7]. The authors conclude that Gulf hawksbills spend about 20% of their time undertaking these summer migration loops, a thermoregulatory response to avoid elevated sea surface temperatures and potentially physiology-threatening conditions. Continued increases in temperature may force turtles to extend such migrations and spend more time in deeper cooler waters, increasing their overall energy demand.

Other important factors that were not considered in the methodology described here and that may significantly affect marine turtle populations are [8-11]:

- climate change impacts to nesting beaches [12, 13] in the Gulf, for those individuals that use the region’s coastline for reproduction. Rising sea levels and increased storm intensity will negatively impact available sea turtle nesting grounds. Around 1000 green female turtles nest annually on Karan and Jana Islands in Saudi Arabia [14, 15], and around 500 hawksbill female turtles nest annually on Jana [14]. Hawksbills also nest at several key sites in Iran (ca. 1000s of females/year) [16, 17], at numerous small islands in the United Arab Emirates [3], and at Fuwairit, Ras Laffan, and Halul in Qatar [18]. Green turtles nest in small numbers in the United Arab Emirates [19]; and both species also nest in small numbers on islands off Kuwait [20, 21]. This segment of the population may also suffer from climate change impacts to its foraging grounds that may be within or outside of the Gulf. Green turtles nesting at Karan and Jana for example have been found foraging off the UAE and the bulk of that stock is likely to reside entirely within the Gulf [5]; and
- climate change impacts to feeding grounds, specifically seagrass beds [22, 23] for green sea turtles and coral reefs [e.g., 24, 25] for hawksbill turtles, for those individuals that depend on the Gulf to forage. Model projections show *casi* no changes in the habitat suitability of *Halodule univervis*, major losses for *Halophila ovalis* around the UAE and the eastern coast of Qatar, and the total disappearance of *H. stipulacea*. Green turtles are known to predominantly forage on the former two, and based on projections, may in the future depend more heavily on *H. uninervis,* currently considered the most common species in the Gulf [26]. Key foraging areas to date include seagrass beds in the UAE, Abu Dhabi in particular, as well as smaller areas in Qatar and Saudi Arabia [5]. Future studies should endeavour to obtain more information on green turtle diets from the region to discern to what extent they depend on different seagrass species and/or algae as well as small benthic invertebrates for forage to help inform how predicted changes may impact turtle fitness in the future. This segment of the population may also suffer from climate change impacts to its nesting grounds that may or may not be in the Gulf.

Overall, the highly migratory nature of marine turtles, and their ability to move considerable distances over short periods of time, should increase their resilience to climate change. In the context of the Gulf, this may mean that turtles may come to spend less time in the region. However, any such resilience is likely to be severely compromised by other anthropogenic influences (e.g., fishing bycatch, loss of nesting and foraging habitat due to pollution, eutrophication and coastal development). This would also hold true for other charismatic species. For example, mapping of historical dugong sightings indicates that the population may have experienced a range contraction of up to 26%, with records found in Kuwait and Iran previously thought not to have dugongs [27].

While dolphins and dugongs have less complicated life cycles than marine turtles, changes in their environment other than sea temperature, such as key prey species, are likely to be more significant in determining their vulnerability to climate change. For dugongs for example, we would expect impacts to their main source of forage, seagrass to be critical. Projections for all three species of seagrass indicate that dugongs may in the future focus their grazing activity on the rhizomes of the two main species, *H. ovalis* and *H. univervis*, and that their distribution may decline around the UAE and Qatar in response to declines in *H. ovalis*. Not surprisingly, the most important habitats for dugongs, occur around Murawah Island (UAE); between Qatar and Bahrain; and between Qatar and the UAE [2]. In turn, the vulnerability of seagrass to climate change is likely to depend on species’ tolerance to changes in temperature and salinity, but it is also arguably most dependent on changes in turbidity, sea level and UV-radiation for example. Light is a key environmental resource imperative for the growth and survival of seagrass. Dredging, infilling and industrial developments, in addition to directly removing large areas of shallow productive benthic habitat, significantly affect turbidity and sedimentation and are considered the greatest threats to this important habitat [26, 28]. In the case of dolphins, their distribution may shift in accordance with changes in the habitat suitability of their key prey. Very little is known about the Indo-Pacific humpback dolphin, with reports of population sizes reaching 1,200 [2], but no absolute measure of abundance for anywhere in the region, and the status of the species currently listed as unknown [28]. To better assess the likely impacts of climate change on Indo-Pacific humpback dolphin beyond projected temperature and salinity as undertaken here, future studies should aim to gain greater ecological understanding of this species in the Gulf and the risks to the species from different threats it is currently exposed to. It is likely that incidental capture in fishing nets, coastal and offshore development, pollution, boat traffic, oil and gas exploration, military exercises, and biotoxins associated with red tide events [29] may cause greater harm to both species of dolphins, and thus be more important to mitigate, in the short and long term, than climate change.

For species such as sea turtles, dugong, or dolphins, the modelling approach presented here presents interesting insights, but will need to be expanded upon to fully assess their vulnerability to climate change. Future analyses should consider including changes in future primary production and combine these with changes in other ecological components important to their distribution (e.g., availability and quality of nesting and foraging grounds in the case of sea turtles). Overall, it is important to note that vulnerability of large megafauna to climate change outcomes are likely to be quite variable and predictions are therefore complex. Current efforts towards long-term monitoring; mix stock studies with in-water surveys; greater understanding of the importance of different areas/habitats for sea turtles and other marine fauna; cross-sector and cross-boundary collaboration between governments, universities and industries together with wide-scale stakeholder engagement; as well as the development of conservation strategies that combine protected areas and the regulation of fishing and shipping activities, are to be improved upon, supported and encouraged. The latter is particularly important given that charismatic species, particularly sea turtles and cetaceans, have suffered from significant anthropogenic impacts other than climate change (71% decline in cetacean abundance in the UAE between 1986 and 1999 [2]) and their curtailment is of key importance to the conservation of species in the region.

### References

1. Al-Abdulrazzak D, Pauly D. Reconstructing historical baselines for the Persian/Arabian Gulf Dugong, Dugong dugon (Mammalia: Sirena). Zoology in the Middle East. 2017;63(2):95-102. doi: 10.1080/09397140.2017.1315853.

2. Preen A. Distribution, abundance and conservation status of dugongs and dolphins in the southern and western Arabian Gulf. Biol Conserv. 2004;118(2):205-18. doi: <http://dx.doi.org/10.1016/j.biocon.2003.08.014>.

3. Pilcher NJ, Antonopoulou M, Perry L, Abdel-Moati MA, Al Abdessalaam TZ, Albeldawi M, et al. Identification of Important Sea Turtle Areas (ITAs) for hawksbill turtles in the Arabian Region. J Exp Mar Bio Ecol. 2014;460:89-99. doi: <http://dx.doi.org/10.1016/j.jembe.2014.06.009>.

4. Jensen MP, Allen CD, Eguchi T, Bell IP, LaCasella EL, Hilton WA, et al. Environmental Warming and Feminization of One of the Largest Sea Turtle Populations in the World. Curr Biol. 28(1):154-9.e4. doi: 10.1016/j.cub.2017.11.057.

5. Pilcher NJ, Al-Maslamani I, Williams J, Gasang R, Chikhi A. Population structure of marine turtles in coastal waters of Qatar. Endangered Species Research. 2015;28(2):163.

6. Sale PF, Feary DA, Burt JA, Bauman AG, Cavalcante GH, Drouillard KG, et al. The Growing Need for Sustainable Ecological Management of Marine Communities of the Persian Gulf. Ambio. 2011;40(1):4-17. doi: 10.1007/s13280-010-0092-6. PubMed PMID: WOS:000286942800002.

7. Pilcher NJ, Perry L, Antonopoulou M, Abdel-Moati MA, Al Abdessalaam TZ, Albeldawi M, et al. Short-term behavioural responses to thermal stress by hawksbill turtles in the Arabian region. J Exp Mar Bio Ecol. 2014;457:190-8. doi: <http://dx.doi.org/10.1016/j.jembe.2014.04.002>.

8. Poloczanska ES, Brown CJ, Sydeman WJ, Kiessling W, Schoeman DS, Moore PJ, et al. Global imprint of climate change on marine life. Nat Clim Change. 2013;3(10):919-25. doi: 10.1038/Nclimate1958. PubMed PMID: WOS:000326818800020.

9. Hawkes LA, Broderick AC, Godfrey MH, Godley BJ. Climate change and marine turtles. Endangered Species Research. 2009;7:137-54.

10. Fuentes MMPB, Hamann M, Limpus CJ. Past, current and future thermal profiles of green turtle nesting grounds: Implications from climate change. J Exp Mar Bio Ecol. 2010;383(1):56-64. doi: 10.1016/j.jembe.2009.11.003. PubMed PMID: WOS:000274071900008.

11. Witt MJ, Hawkes LA, Godfrey MH, Godley BJ, Broderick AC. Predicting the impacts of climate change on a globally distributed species: the case of the loggerhead turtle. J Exp Biol. 2010;213(6):901-11. doi: 10.1242/jeb.038133. PubMed PMID: WOS:000275002600012.

12. Fish MR, Côté IM, Gill JA, Jones AP, Renshoff S, Watkinson AR. Predicting the Impact of Sea-Level Rise on Caribbean Sea Turtle Nesting Habitat. Conserv Biol. 2005;19(2):482-91. doi: 10.1111/j.1523-1739.2005.00146.x.

13. Fuentes MMPB, Limpus CJ, Hamann M. Vulnerability of sea turtle nesting grounds to climate change. Glob Chang Biol. 2011;17(1):140-53. doi: 10.1111/j.1365-2486.2010.02192.x. PubMed PMID: WOS:000284851500012.

14. Pilcher NJ. The hawksbill turtle, Eretmochelys imbricata, in the Arabian Gulf. Chelonian Conserv Biol. 1999;3(2):312-7.

15. Al-Merghani M, Miller JD, Al-Mansi A, Pilcher N. The green and hawksbill turtles in the Kingdom of Saudi Arabia: Synopsis of nesting studies 1986-1997. Fauna of Arabia. 2000;18:369-84.

16. Mobaraki A. Marine turtles in Iran: results from 2002. Marine Turtle Newsletter. 2004;104(13).

17. Mobaraki A, Elmi AM. First sea turtle tagging programme in Iran. Marine Turtle Newsletter. 2005;110:6-7.

18. Tayab MR, Quiton P. Marine turtle conservation initiatives at Ras Laffan Industrial City, Qatar (Arabian Gulf). Marine Turtle Newsletter. 2003;99(14-15).

19. Al-Suweidi AS, Wilson KDP, Healy T, Vanneyre L. First contemporary record of green turtle (Chelonia mydas) nesting in the United Arab Emirates. Marine Turtle Newsletter 2012;133:16-7.

20. Al-Mohanna S, George P. Status of sea turtles in Kuwait. Marine conservation forum-EWS-WWF, 11-14 September’06 2006.

21. Rees AF, Al Hafez A, Lloyd JR, Papathansopoulou N, Godley BJ. Green Turtles, Chelonia mydas, in Kuwait: Nesting and Movements. Chelonian Conserv Biol. 2013;12(1):157-+. PubMed PMID: WOS:000322330100017.

22. Orth RJ, Carruthers TJB, Dennison WC, Duarte CM, Fourqurean JW, Heck KL, et al. A global crisis for seagrass ecosystems. Bioscience. 2006;56(12):987-96. PubMed PMID: ISI:000242795000008.

23. Waycott M, Duarte CM, Carruthers TJB, Orth RJ, Dennison WC, Olyarnik S, et al. Accelerating loss of seagrasses across the globe threatens coastal ecosystems. Proc Natl Acad Sci U S A. 2009;106(30):12377-81. PubMed PMID: WOS:000268440200033.

24. Burt JA, Al-Khalifa K, Khalaf E, AlShuwaikh B, Abdulwahab A. The continuing decline of coral reefs in Bahrain. Mar Pollut Bull. 2013;72(2):357-63. doi: 10.1016/j.marpolbul.2012.08.022. PubMed PMID: WOS:000323021100008.

25. Wilson S, Fatemi SMR, Shokri MR, Claereboudt M. Status of coral reefs of the Persian/Arabian Gulf and Arabian Sea region. In: Wilkinson CR, editor. Status of coral reefs of the world: 2002: GCRMN Report. Australian Institute of Marine Science, Townsville; 2002. p. 53-62.

26. Erftemeijer PLA, Shuail DA. Seagrass habitats in the Arabian Gulf: distribution, tolerance thresholds and threats. Aquatic Ecosystem Health & Management. 2012;15:73-83. doi: 10.1080/14634988.2012.668479. PubMed PMID: WOS:000304834700013.

27. Al-Abdulrazzak D. In the wake of the dhow: historical changes in the marine ecology and fisheries of the Persian Gulf: University of British Columbia, Vancouver, Canada 2015.

28. Erftemeijer PLA, Robin Lewis Iii RR. Environmental impacts of dredging on seagrasses: A review. Mar Pollut Bull. 2006;52(12):1553-72.

29. Baldwin RM, Collins M, Van Waerebeek K, Minton G. The Indo-Pacific humpback dolphin of the Arabian region: a status review. Aquatic Mammals. 2004;30(1):111-24.
