# Supplementary material for: Climate change impacts on marine biodiversity, fisheries and society in the Arabian Gulf
Source: PLoS One. 2018 May 2;13(5):e0194537. doi: 10.1371/journal.pone.0194537 (PMC5931652; doi:10.1371/journal.pone.0194537)
Supplement: S2 Appendix — (DOCX) [file pone.0194537.s007.docx]

### S2 Appendix

#### **Fishery catch reconstructions**

Catch data used in all analyses consisted of “reconstructed” fisheries catch data for each country in the Gulf region [1-3]. These reconstructions are part of the global, country-by-country research effort conducted by the *Sea Around Us* [4], and principally add comprehensive, but conservative catch estimates for all unreported fisheries components to the official landings statistics reported by FAO on behalf of countries [e.g., 5, 6-9].

#### **Reconstruction methodology**

Country-level catch reconstructions are as independent from each other as possible (to avoid systematic biasing), but follow the general and well-established reconstruction principles by starting in 1950, covering all fisheries sectors that exist in a country, and including at least minimal estimates of discards for major fisheries [4]. As all reconstructions cover individual Exclusive Economic Zones (EEZ), including the entire EEZ or several separate EEZ components differentiated by ecosystem or political considerations [10, 11], it was possible, for Gulf countries with access to waters other than the Gulf (e.g. UAE, Iran), to include catches derived from the Gulf only in our analyses (S3 Table and see below). Reconstructions provide both the reported catches as well as best estimates of unreported catches, all segregated by industrial (large-scale, commercial), artisanal (small-scale, commercial), recreational and subsistence (both small-scale, non-commercial) sectors. Reconstructions also estimate the volume of discards from major fisheries in each country (fish caught, but discarded at sea) as part of a global discard analysis [12], which for Gulf countries ranged from very little (e.g., 0.6% of total catches in the UAE [3, 10]), to rather substantial (e.g., 80% of total catches in Kuwait [3, 13]). Every country’s reconstruction details and data sources are described thoroughly in dedicated technical reports (e.g., Al-Abdulrazzak [14]) freely available at www.seaarounduus.org, and also in scientific articles [e.g., 1].

We improved upon the spatial resolution of reconstructed catches by allocating EEZ-level reconstructed data to the global 0.5^o^ latitude x 0.5^o^ degree longitude grid system of the *Sea Around Us* (i.e., 180,000 maritime ½ degree cells globally) using a rule-based spatial allocation approach [4]. This method assigns EEZ-level catches to half-degree cells based on two major constraints: 1) the biological probability distribution for each taxon in the catch data, as derived for all taxa, via the best available habitat and biogeographic information, as provided via FishBase [15] and SeaLifeBase [16]; and 2) the accessibility of foreign-flagged fleets to the EEZs of each country, as provided via explicit (e.g., negotiated fishing access agreements) or implicit (e.g., observed or traditionally recognized or tolerated) access [4]. Prior to the allocation procedure, we pre-assigned domestic small-scale fisheries catches in every country to the Inshore Fishing Area (IFA) within the EEZ, defined as the waters within 50 km from shore or waters up to 200 m depth, whichever comes first [4, 17]. These reconstruction-derived restrictions to EEZ and domestic IFA waters prevent domestic catches of one country showing up in the EEZs of the wrong countries [4], or small-scale fisheries catches being assigned unrealistically far offshore. The end result of the spatial allocation process of all reconstructed catch data is that catches are as comprehensively as possible assigned to small spatial units (½ degree cells) that are relatively meaningful in both ecological terms and political fishing access reality. We derived EEZ boundaries or EEZ-equivalent claims based either on existing EEZ claims by a given country as known to us, or based on the basic principles underlying the United Nation Convention on the Law of the Sea (UNCLOS) as related to EEZs (i.e., 200 nautical miles or midline). The maritime limits and EEZ boundaries used here are not to be considered as an authority on the delimitation of international maritime boundaries, and cannot be taken as politically or legally representative. For time-periods pre-dating EEZ declarations, we assumed free access to these waters, but treat them as EEZ-equivalent. Note finally, that territorial waters (i.e., to 3, 6 or 12 nautical miles) are here included in the EEZs.

#### **Results**

As presented in Al-Abdulrazzak and Pauly [14] and Al-Abdulrazzak et al. [3], total catches from the Arabian Gulf increased from 1950 to 2000 from 200,000 t∙year^-1^ to around 600,000 t∙year^-1^, then declined to about 400,000 t in 2005 (Fig 1). Gulf countries have primarily reported their commercial (i.e., artisanal and industrial) catches and have substantially misreported their recreational, subsistence, and illegal fishing sectors. As with most countries in the world, discards are also not included in official reported statistics. Results suggest that all countries in the Gulf under-report their catches, with the exception of the UAE, which partially over-reported theirs, especially in earlier decades [10]. Regionally, officially reported catches potentially underestimate capture fisheries catches by a factor of two between 1950 and 2010, and that discards, mainly from shrimp trawlers, correspond to 18% of total landed catch. A summary of the catch reconstruction findings, by relevant country for this study, is presented below.

| A | 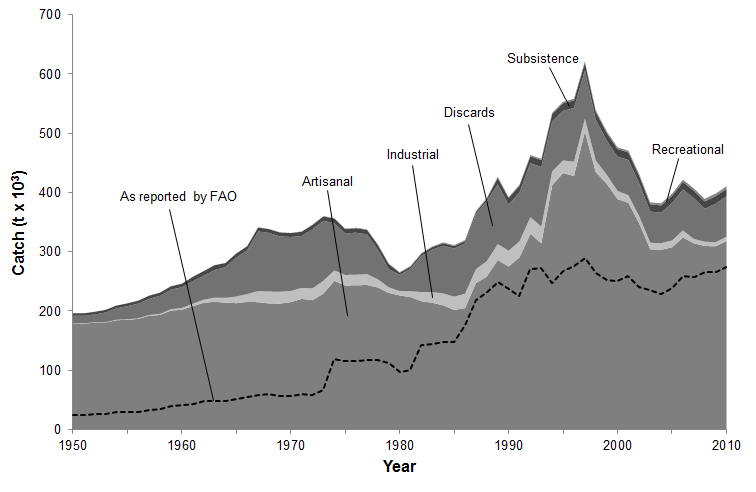 |
| --- | --- |
| B | 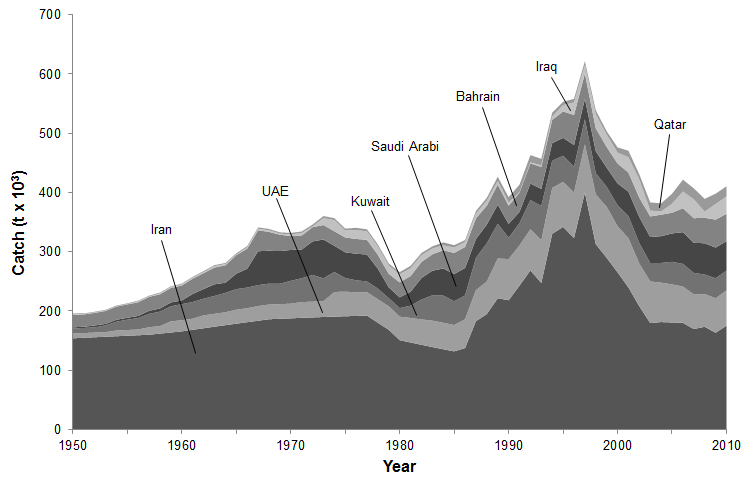 |
| C | 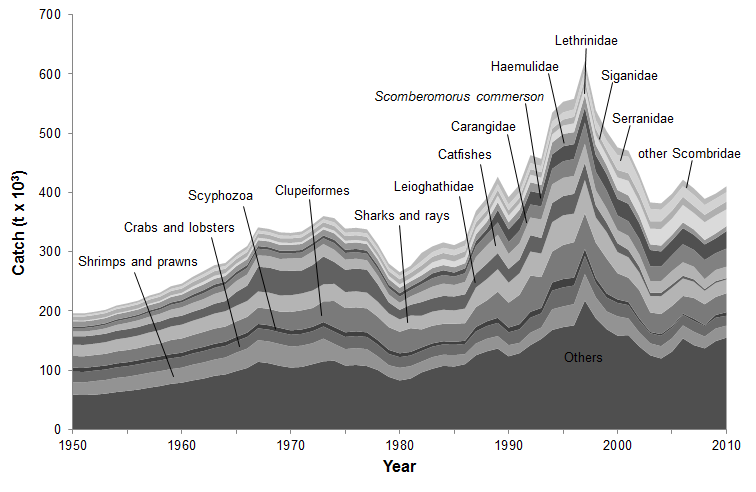 |
| Fig 1 Total reconstructed catches from the Arabian Gulf region from 1950 to 2010. Reconstructed catches are presented by fishing sector (A), by countries (B) and by major exploited taxa. | |

### Bahrain

Bahrain is the smallest of the Gulf States and the only island country in the region. Bahrain has a rich maritime history that includes fishing and pearling. The fisheries consist of an important artisanal sector, whose catches dramatically increased in the 2000s, in contrast to industrial catches that appear to plummet (Fig 2). The reconstructed catch of Bahrain, based on Al-Abdulrazzak [18], increased from 6,200 t in 1950 to 48,000 t in 1996, then declined and increased again to similar levels in the late 2000s. Reconstructed total catch corresponds to about 3.5 times landings reported by FAO on behalf of Bahrain. Rabbitfish (Family Siganidae), swimming crab (Portunidae), herring, shad, sardine and menhaden (Clupeidae), and fourlined terapon (*Pelates quadrilineatus*) were the major taxa in the catch, which also included a multitude of other species (Fig 2). There are growing concerns over Bahrain’s fisheries, including their continued use of illegal driftnets. Although catches appear to be increasing, it is likely that the decline of traditionally targeted taxa is masked by previously discarded species being retained for consumption by the increasing immigrant community in Bahrain.

**
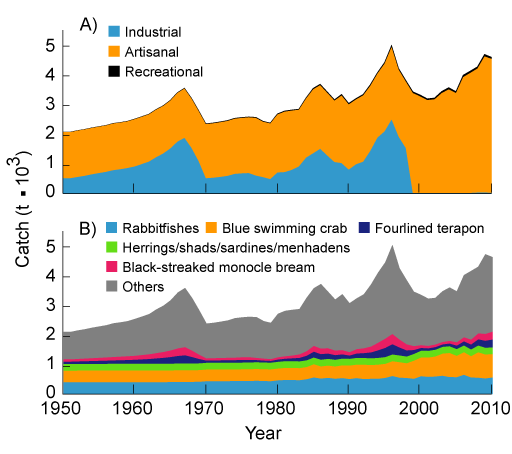
**

Fig 2 Domestic and foreign catches taken in the EEZ of Bahrain. (A) by sector; (B) by taxon.

### Iraq

Iraq has the smallest EEZ of the Gulf countries, at the mouth of the Shatt al-Arab River, formed by the confluence of the Euphrates and Tigris rivers about 200 km upstream. Consequently, Iraq’s marine fisheries are less important than its freshwater fisheries, not considered here [see 19]. Catches were predominately domestic and artisanal in nature, although the subsistence sector does exist and was reconstructed by Al-Abdulrazzak and Pauly [20], based on admittedly fragmentary evidence (Fig 3A). Domestic catches were in the order of 1,000-3,000 t·year^-1^ from 1950 to the early 1970s, then fluctuated between 10,000 and 30,000 t·year^-1^, as peace and war alternated in the Shatt-al-Arab region. Overall, the reconstructed catches are 1.6 times those reported by FAO on behalf of Iraq, and are dominated by previously unreported catches of hilsa shad (*Tenualosa ilisha*), an anadromous fish. The rest of the catch is likely to resemble that of neighbouring Kuwait, whose taxonomic composition was used to disaggregate the non-hilsa marine catch of Iraq (Fig 3B), and thus assumed to consist of groups such as mullets (Family Mugilidae), croakers (Family Sciaenidae) and groupers (*Epinephelus* spp.).

**
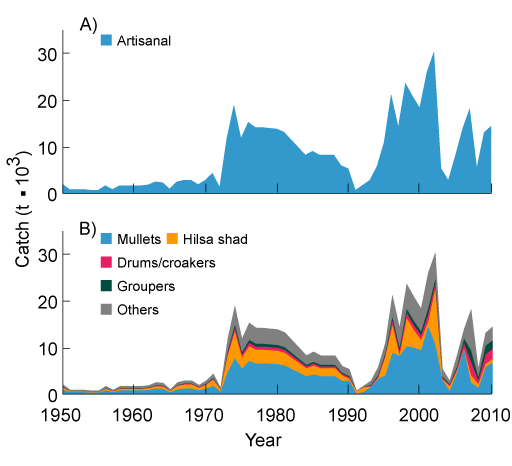
**

Fig 3 Domestic and foreign catches taken in the EEZ of Iraq. (A) by sector; (B) by taxon.

### Iran (Gulf only)

Iran is the country with the longest coastline in the Arabian Gulf. This catch reconstruction, adapted from Roshan Moniri *et al.* [21], concerns only Iran’s fisheries in the Gulf and covers domestic industrial, artisanal (incl. weirs; [1]), subsistence, recreational, and discards) (Fig 4A). Although the majority of catches within the EEZ are domestic, foreign catches by China, South Korea and some of Iran’s neighbours were also documented starting in the 1980s (Fig 4B). The reconstructed domestic catch averaged 157,000 t·year^-1^ in the 1950s, slowly increased to 192,000 t before declining during the Iran-Iraq war (1980-1988), rapidly recovered and peaked at 400,000 t in 1997. It declined to around 170,000 t∙year^-1^ in the late 2000s. These values correspond to a reconstructed catch 2.7 times the data reported by FAO (and adjusted for Iranian catches from outside of the Gulf), largely due to substantial under-reporting of artisanal catches. Catches were dominated by ponyfish (*Leiognathus* spp.), green tiger prawn (*Penaeus semisulcatus*) and blue swimming crab (*Portunus segnis*), but also included a tremendous variety of other fish species (Fig 4C). Overall, Iran’s nominal management of fisheries is hampered by lack of key data, and the suppression of domestic and foreign illegal fishing.


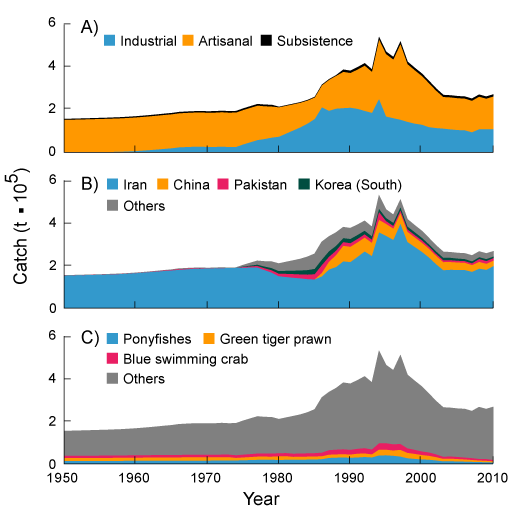


Fig 4 Domestic and foreign catches taken in the EEZ of Iran (Gulf). A) by sector; B) by fishing country; C) by taxon.

### Kuwait

Kuwait is located in the northwest of the Arabian Gulf. Substantial artisanal and industrial fishing occurs in Kuwait’s EEZ, in addition to a large subsistence and recreational component (although not visible in Fig 5A). Kuwait’s catches have grown substantially over the past 60 years [22], and were reconstructed by Al-Abdulrazzak [13]. The result is a total domestic catch estimate of about 8,700 t·year^-1^ for the early 1950s, increasing to a first peak of over 40,000 t·year^-1^ in the early 1970s, followed by decline in the late 1970s, due to the Iran-Iraq war. The second peak, at over 60,000 t, occurred in 1988, and was followed by a slow decline in total catches stabilizing at under 40,000 t·year^-1^ through the 2000s. Overall, this corresponded to a catch 6.4 times that reported by FAO on behalf of Kuwait, mainly due to discards from the trawl fishery being 10 times greater than finfish landings. Foreign fishing, notably by Iraq, only becomes visible in the 2000s (Fig 5B). Fig 5C shows a few of the major taxa caught in Kuwait: giant catfish (*Netuma thalassina*) whose catch is entirely discarded; sharks and rays (Elasmobranchii); natantian decapods (shrimps); and guitarfish (Rhinobatidae), also discarded. One of the first key management actions for Kuwait would be to find a way to utilize the vast by-catch of Kuwait’s trawl fisheries that is currently discarded.

**
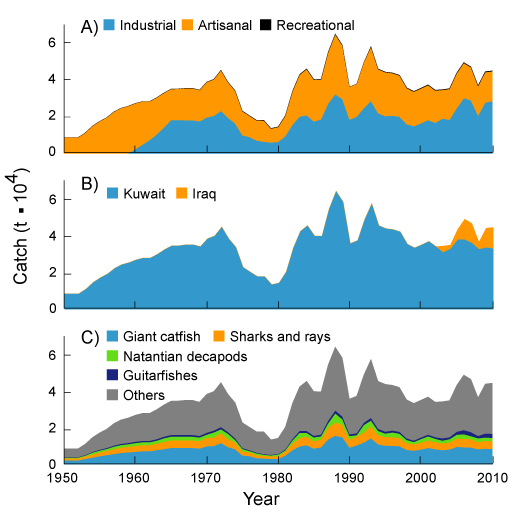
**

Fig 5 Domestic and foreign catches taken in the EEZ of Kuwait. (A) by sector; (B) by fishing country; and (C) by taxon.

### Qatar

Qatar is a small country located on a peninsula abutting Saudi Arabia. Qatar’s catches have increased sharply over the past decade due to increased fishing effort, driven by the growing demand from a rapidly expanding population. Catch reconstruction by Al-Abdulrazzak [23] suggests that domestic landings in the 1950s were about 1,000 t·year^-1^, increased to nearly 20,000 t·year^-1^ in the 2000s, and were 38% higher than reported by the FAO on behalf of Qatar. While catches in the earlier years were dominated by industrial vessels (Fig 6A), mainly of foreign origin prior to EEZ declaration in 1974, more recently, domestic artisanal fleets predominate (Fig 6B). One of several reasons for the discrepancy between domestic reported and reconstructed catches is the omission of discards from Qatar’s bottom trawl fishery. Between 1970 and 1993, the 3 bottom trawlers operated by the Qatari National Fishing Company (QNFC) discarded the equivalent of 30% of reported catches. The main taxa caught by Qatar are emperors (Family Lethrinidae), groupers (Serranidae), jacks (Carangidae), rabbitfish (Siganidae) and grunts (Haemulidae; Fig 6C). The reconstruction also highlighted the extent of illegal domestic fishing. For example, 14 tidal weirs (‘*hadrah*’), a practice that has been banned since 1994, were detected by Al-Abdulrazzak and Pauly [1] along the Qatari coast on current Google Earth images.

**
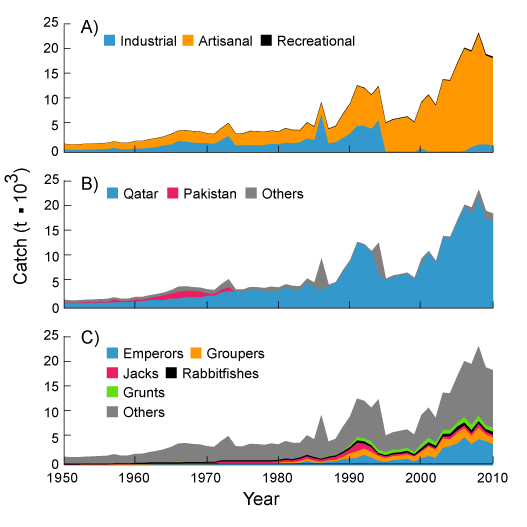
**

Fig 6 Domestic and foreign catches taken in the EEZ of Qatar. (A) by sector; (B) by fishing country; and (C) by taxon.

### Saudi Arabia (Gulf only)

Saudi Arabia has a longer coastline in the Red Sea than in the Gulf, but its Gulf fish catch is still considerable. The bulk of Saudi fisheries in the Gulf are artisanal (Fig 7A); their motorization started in the early 1960s and was completed in the late 1980s. Domestic catches by Saudi Arabia in the Gulf were reconstructed by Tesfamichael and Pauly [24] primarily based on data from the Regional Commission for Fisheries (RECOFI). Landings were found to increase from about 2,000 t·year^-1^·in the early 1950s to 50,000 t·year^-1^ in the late 2000s, with an intermediate phase, from the early 1960s to the early 1990s. High trawl catches and especially discards, contributed substantially to overall catches from 1950 to 2010. While the reconstructed Saudi catch as a whole (i.e., including catches in the Red Sea) is 2.1 times higher than FAO reports on behalf of Saudi Arabia, estimates are 2.4 times higher for catches in the Gulf only. The domestic fishery landed the majority of the catch (Fig 7B). Due to the nature of artisanal fisheries and Saudi’s Gulf waters, which are generally shallow with sandy and muddy bottoms covered by seagrass beds, the catch (Fig 7C) consists mainly of a multitude of demersal fish and shrimp, both impacted by oil pollution, notably in 1991 [25].

**
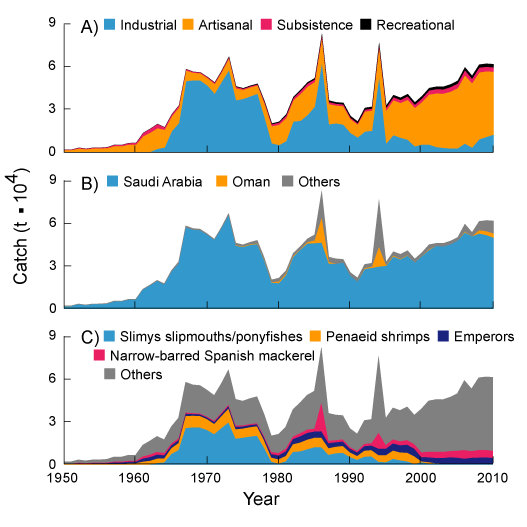
**

Fig 7 Domestic and foreign catches taken in the EEZ of Saudi Arabia. (A) by sector; (B) by fishing country; and (C) by taxon.

### United Arab Emirates (Gulf only)

The United Arab Emirates (UAE) has coasts on the Arabian Gulf and in the Gulf of Oman. Catch reconstructions presented here, and based on Al-Abdulrazzak [10], only address landings for the UAE’s Gulf coast. Domestic fisheries are small-scale in nature (Fig 8A), with little foreign or industrial fishing (Fig 8B), and occur mostly in Abu Dhabi, which comprises over 60% of the UAE’s Gulf EEZ. Due to its reliance on a market-sampling program that did not differentiate between locally caught and imported seafood, the UAE systematically over-reported its catches [26]. Al-Abdulrazzak [10], who considered this, estimated the UAE’s catch, adjusted for Gulf EEZ waters only, as 8,000·t·year^-1^ in the early 1950s, increasing until reaching a peak at 80,000 t in 1999, then declining to 50,000 t·year^-1^ in the late 2000s. Overall, the figures reported by FAO on behalf of the entire UAE *over*-estimated reconstructed catches by 47%, despite the latter also accounting for subsistence and recreational catches, missed entirely by the market-sampling program. The major taxa caught in the UAE (Fig 8C) are mackerel and tuna (Family Scombridae), herring (Clupeidae), jacks (Carangidae), emperors (Lethrinidae), narrow-barred Spanish mackerel (*Scomberomorus commerson*) and groupers (Serranidae). Improving the UAE’s catch reporting system appears essential if its fisheries are to be managed for sustainability.


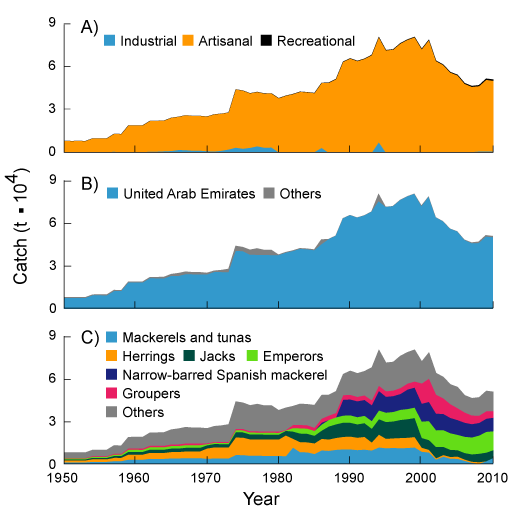


Fig 8 Domestic and foreign catches taken in the EEZ of United Arab Emirates. A) by sector; (B) by fishing country; and (C) by taxon.

### References

1. Al-Abdulrazzak D, Pauly D. Managing fisheries from space: Google Earth improves estimates of distant fish catches. ICES Journal of Marine Science. 2014;71(3):450-4. doi: <https://doi.org/10.1093/icesjms/fst178>.

2. Al-Abdulrazzak D, Pauly D. Ground-truthing the ground-truth: reply to Garibaldi et al.'s comment on “Managing fisheries from space: Google Earth improves estimates of distant fish catches”. ICES Journal of Marine Science. 2014;71(7):1927-31. doi: 10.1093/icesjms/fsu103.

3. Al-Abdulrazzak D, Zeller D, Belhabib D, Tesfamichael D, Pauly D. Total marine fisheries catches in the Persian/Arabian Gulf from 1950 to 2010. Regional Studies in Marine Science. 2015;2:28-34. doi: <http://dx.doi.org/10.1016/j.rsma.2015.08.003>.

4. Zeller D, Palomares MLD, Tavakolie A, Ang M, Belhabib D, Cheung WWL, et al. Still catching attention: Sea Around Us reconstructed global catch data, their spatial expression and public accessibility. Mar Policy. 2016;70:145-52. doi: 10.1016/j.marpol.2016.04.046. PubMed PMID: WOS:000379371500017.

5. Khalfallah M, Zylich K, Zeller D, Pauly D. Reconstruction of marine fisheries catches for Oman (1950-2014). Front Mar Sci. 2016;3(152):doi: 10.3389/fmars.2016.00152.

6. Smith NS, Zeller D. Unreported catch and tourist demand on local fisheries of small island states: the case of The Bahamas 1950-2010. Fishery Bulletin. 2016;114:117-31.

7. Piroddi C, Gristina M, Zylich K, Greer K, Ulman A, Zeller D, et al. Reconstruction of Italy’s marine fisheries removals and fishing capacity, 1950-2010. Fisheries Research. 2015;172:137-47.

8. Zeller D, Harper S, Zylich K, Pauly D. Synthesis of under-reported small-scale fisheries catch in Pacific-island waters. Coral Reefs. 2015;34(1):25-39.

9. Coll M, Carreras M, Cornax MJ, Massutí E, Morote E, Pastor X, et al. Closer to reality: reconstructing total removals in mixed fisheries from Southern Europe. Fisheries Research. 2014;154:179-94.

10. Al-Abdulrazzak D. Estimating total fish extractions in the United Arab Emirates: 1950-2010. In: Al-Abdulrazzak D, Pauly D, editors. From dhows to trawlers: a recent history of fisheries in the Gulf countries, 1950 to 2010: Fisheries Centre Research Reports 21(2). University of British Columbia Fisheries Centre, Vancouver; 2013. p. 31-7.

11. Khalfallah M, Zeller D, Pauly D. Reconstruction of marine fisheries catches for Fujairah (UAE) (1950-2010). Fisheries Centre Working Paper #2015-57, University of British Columbia, Vancouver, 2015.

12. Zeller D, Cashion T, Palomares M, Pauly D. Global marine fisheries discards: A synthesis of reconstructed data. Fish and Fisheries. 2018;19(1):30-9. doi: 10.1111/faf.12233.

13. Al-Abdulrazzak D. Reconstructing Kuwait’s marine fishery catches: 1950-2010. In: Al-Abdulrazzak D, Pauly D, editors. From dhows to trawlers: a recent history of fisheries in the Gulf countries, 1950 to 2010: Fisheries Centre Research Reports 21(2). University of British Columbia Fisheries Centre, Vancouver; 2013.

14. Al-Abdulrazzak D, Pauly D, editors. From dhows to trawlers: a recent history of fisheries in the Gulf countries, 1950 to 2010: Fisheries Centre Research Reports 21(2). University of British Columbia Fisheries Centre, Vancouver 2013.

15. Fishbase [Internet]. 2016 [cited 20 March 2016]. Available from: [www.fishbase.org](http://www.fishbase.org).

16. SeaLifeBase [Internet]. 2016 [cited 20 March 2016]. Available from: [www.sealifebase.org](http://www.sealifebase.org).

17. Chuenpagdee R, Liguori L, Palomares MLD, Pauly D. Bottom-up, global estimates of small-scale marine fisheries catches. Fisheries Centre Research Reports 14(8), University of British Columbia, Vancouver: 2006.

18. Al-Abdulrazzak D. Missing sectors from Bahrain’s reported fisheries catches: 1950-2010. In: Al-Abdulrazzak D, Pauly D, editors. From dhows to trawlers: a recent history of fisheries in the Gulf countries, 1950 to 2010: Fisheries Centre Research Reports 21(2). University of British Columbia Fisheries Centre, Vancouver; 2013. p. 1-6.

19. Jawad LA. Fishing gear and methods of the lower Mesopotamian Plain with reference to fishing management. Marina Mesopotamica. 2006;1(1):1-37.

20. Al-Abdulrazzak D, Pauly D. Reconstructing Iraq’s fisheries: 1950-2010. In: Al-Abdulrazzak D, Pauly D, editors. From dhows to trawlers: a recent history of fisheries in the Gulf countries, 1950 to 2010: Fisheries Centre Research Reports 21(2). University of British Columbia Fisheries Centre, Vancouver; 2013. p. 17-22.

21. Roshan Moniri N, Zeller D, Al-Abdulrazzak D, Zylich K, Belhabib D. Fisheries catch reconstruction for Iran, 1950-2010. In: Al-Abdulrazzak D, Pauly D, editors. From dhows to trawlers: a recent history of fisheries in the Gulf countries, 1950 to 2010. Fisheries Centre Research Reports 21(2) 2013. p. 7-16.

22. Al-Sabbagh T, Dashti J. Post-invasion status of Kuwait’s fin-fish and shrimp fisheries (1991-1992). World Journal of Fish and Marine Sciences 2009;1(2):94-6.

23. Al-Abdulrazzak D. Total fishery extractions for Qatar: 1950-2010. In: Al-Abdulrazzak D, Pauly D, editors. From dhows to trawlers: a recent history of fisheries in the Gulf countries, 1950 to 2010: Fisheries Centre Research Reports 21(2). University of British Columbia Fisheries Centre, Vancouver; 2013. p. 31-7.

24. Tesfamichael D, Pauly D. Catch reconstruction of the fisheries of Saudi Arabia in the Gulf, 1950-2010. From dhows to trawlers: a recent history of fisheries in the Gulf countries, 1950 to 2010. Fisheries Centre Research Reports 21(2): Fisheries Centre, University of British Columbia, Vancouver; 2013. p. 39-52.

25. Mathews CP, Kedidi S, Fita NI, Alyahya A, Alrasheed K. Preliminary Assessment of the Effects of the 1991 Gulf-War on Saudi-Arabian Prawn Stocks. Mar Pollut Bull. 1993;27:251-71. PubMed PMID: WOS:A1993MQ72200031.

26. Morgan G. Country review: United Arab Emirates. In: de Young C, editor. Review of the state of world marine capture fisheries management: Indian Ocean: FAO Fisheries Technical Paper 488. FAO, Rome 2004. p. 327-35.
