# Supplementary material for: Climate change impacts on marine biodiversity, fisheries and society in the Arabian Gulf
Source: PLoS One. 2018 May 2;13(5):e0194537. doi: 10.1371/journal.pone.0194537 (PMC5931652; doi:10.1371/journal.pone.0194537)
Supplement: S3 Appendix — (DOCX) [file pone.0194537.s008.docx]

#### S3 Appendix

#### Vulnerability indicators: Exposure, Sensitivity and Adaptive Capacity

#### Exposure

Maximum catches (average of the top ten years from 1950 to 2010) of each exploited marine species for each Exclusive Economic Zone within the Gulf was determined based on results from the Gulf catch reconstruction [see Appendix S2 and 1]. We assume that maximum catch is a proxy of maximum sustainable yield (MSY) [2, 3], and that the carrying capacity (K) of the environment for each population is proportional to the suitability of their habitat. Assuming logistic growth, the equilibrium MSY is proportional to carrying capacity.

We calculated the projected changes in average habitat suitability for each population between 2010 (current; average of 2000-210) and 2090 (future; average of 2090-2100). The estimated changes in habitat suitability (ΔHabSuit) were then used to project future MSY where MSY_2090_ = ΔHabSuit x MSY_2010_. We assumed that fisheries management does not adapt to climate change; thus reference points of biomass and exploitation rate at MSY remain constant over time for each population under climate change.

The model generated the current (2010) and the future (2090) catch potential of each marine species for each country’s EEZ) under the GFDL RCP 8.5 scenario. We then projected the future catch by applying the percentage change in the catch potential to the current reconstructed catch amount (2010) in each EEZ (Table 1). The final percentage change in catch of each EEZ can then be estimated.

Table 1. Percentage change in catch potential from each country’s EEZ in 2090 under the RCP 8.5 scenario. Results are presented for each spatial distribution model, for the average of the three models and the standard deviation (SD) around the average.

| **Percentage change in catch potential (%)** | | | | | |
| --- | --- | --- | --- | --- | --- |
|  | **Spatial distribution models** | | |  |  |
| **Country** | **NPPEN** | **ENFA** | **BIOCLIM** | **Average** | **SD** |
| Bahrain | 1.29 | 1.27 | -28.83 | -8.76 | 17.39 |
| Iran | 1.11 | -2.23 | -22.71 | -7.94 | 12.90 |
| Iraq | - | 10.87 | - | 10.87 | 0 |
| Kuwait | 17.50 | 1.29 | 1.53 | 6.77 | 9.29 |
| Oman | -44.08 | -27.55 | -51.41 | -41.01 | 12.22 |
| Qatar | -33.44 | -15.22 | -48.17 | -32.28 | 16.51 |
| Saudi Arabia | -10.43 | -6.14 | -45.06 | -20.54 | 21.34 |
| United Arab Emirates | -63.09 | -28.32 | -44.49 | -45.30 | 17.40 |

To address the uncertainty in projecting habitat suitability, we used a range of species distribution models in our analysis including BIOCLIM, NPPEN, and ENFA [4]. The final percentage change in catch within each EEZ is the average value of these three species distribution models (Fig 1). The final change in catch values were then normalized on a scale from 0 to 1 based on the average minimum and maximum change in catch values from the 3 species distribution models. The normalized values were then subtracted from 1 (i.e., greater changes in catch represent higher levels of exposure) (Table 2).


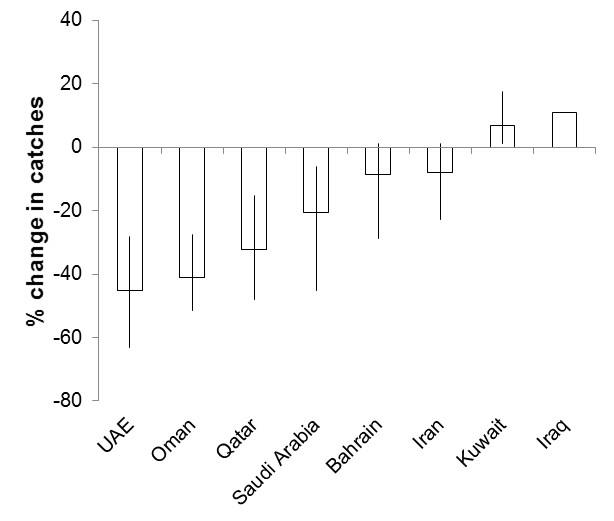


Figure 1. Change in catch potential in the Economic Exclusive Zones (EEZs) of the Gulf in 2090. Results are presented for RCP 8.5 as an average of the three niche models used in this study, NPPEN, ENFA and BIOCLIM. The error bars denote inter-model range.

Table 2. Exposure index for each country in the Gulf

| **Country** | **Exposure** | **Rankings** |
| --- | --- | --- |
| Bahrain | 0.40 | **5** |
| Iran | 0.39 | **6** |
| Iraq | 0.10 | **8** |
| Kuwait | 0.16 | **7** |
| Oman | 0.90 | **2** |
| Qatar | 0.76 | **3** |
| Saudi Arabia | 0.58 | **4** |
| United Arab Emirates | 0.96 | **1** |

#### Sensitivity

In general, *sensitivity* is defined as the degree to which biophysical, social and economic conditions are likely to be directly or indirectly affected by exogenous stresses or hazards (i.e., climate change) [5]. In the context of climate change and fisheries, we adopted the definition of Allison *et al.* [6] in which sensitivity is described as the importance of fisheries to national economies and food security. We therefore derived a composite indicator comprised of the contribution of fisheries to *employment*; the importance of *fish as the source of nutrition*; *economic dependence* of the society *on fisheries*; and more broadly *the importance of marine ecosystem services* in minimising risks and threats from climate change (Table 3). The framework assumes that countries with higher catch and higher dependence on fisheries for economies and food are more likely to be affected by changes to local marine resources under climate change. It is important to note that Oman, Iran and Saudi Arabia have EEZs in, and thus obtain catches from, seas other than the Gulf (i.e., Indian Ocean, Red Sea and the Caspian Sea). As such, all variables, where applicable and for relevant countries, were sub-divided by the proportion of total catches derived from the Gulf only.

Table 3. Sensitivity index and the standardized values of its composite indicators*

|  | **Employment** | | **Nutritional dependence** | | **Economic dependence** | | | | **Coastal dependence** | |  |
| --- | --- | --- | --- | --- | --- | --- | --- | --- | --- | --- | --- |
| **Country** | **Employment** | **Fishers (%)** | **Fish protein (%)** | **Malnourished children**  **(%)** | **Landed values (%)** | **Export values (%)** | **Catch** | **Poverty rate (%)** | **Population in coastal area <5m (%)** | **Coastal land area at <5m (%)** | **Sensitivity index (ranking)** |
| Bahrain | 0.46 | 1 | - | 0.82 | 1.00 | 1.00 | 0.30 | 0.00 | 1.00 | 1.00 | 0.73 **(1)** |
| Iran | 1.00 | 0.30 | 0.50 | 0.29 | 0.09 | 0.88 | 1.00 | 0.65 | 0.06 | 0.03 | 0.48 **(2)** |
| Iraq |  |  | 0.44 | 0.98 | 0.08 | 0.01 | 0.08 | 1.00 | 0.08 | 0.09 | 0.34 **(4)** |
| Kuwait | 0.21 | 0.00 | 0.52 | 0.00 | 0.04 | 0.00 | 0.30 | - | 0.33 | 0.22 | 0.18 **(6)** |
| Oman | 0.00 | 0.03 | 0.00 | 1.00 | 0.00 | 0.06 | 0.00 | - | 0.07 | 0.02 | 0.13 **(7)** |
| Qatar | 0.05 | 0.49 | - | 0.32 | 0.04 | 0.10 | 0.07 | - | 0.34 | 0.34 | 0.22 **(5)** |
| Saudi Arabia | 0.05 | 0.03 | - | 0.41 | 0.00 | 0.08 | 0.14 | 0.14 | 0.00 | 0.00 | 0.09 **(8)** |
| United Arab Emirates | 0.15 | 0.36 | 1.00 | - | 0.05 | 0.67 | 0.30 | 0.71 | 0.10 | 0.11 | 0.38 **(3)** |

*see section below for a detailed description of all variables

Each of the above indicators was derived from a series of variables that are further discussed below. The unweighted average of these variables was taken with the resulting value as the composite index of sensitivity. The indices of sensitivity for each country were then normalized and rescaled from 0 to 1, with higher values representing higher sensitivity to climate change (Table 3).

##### Employment

The contribution of fisheries to national employment is represented by two parameters: the *number of fishers in the fisheries sector* and the *number of people involved in fisheries relative to other economic sectors*. Total direct employment in marine fisheries quantifies the number of people involved in marine fishing, including those reported in national statistics and fishers in the small-scale (artisanal and subsistence) sector whose numbers are typically unreported or under-reported [7] (Table 4).

The number of people involved in fisheries relative to other economic sectors is represented by the proportion of the economically active population (%) in the fishery sector. This indicator captures the extent to which people rely on fishing as a main source of livelihood (Table 5). The higher the proportion of people involved in fisheries, the more sensitive the population is to a downturn in fisheries in the future.

Table 4. Marine fisheries employment estimated for 2003. Data from [7]

| **Country** | **Reported employment** | **Number of people in small-scale fisheries (estimate)** | **Total direct employment** | **Total employment in Gulf** |
| --- | --- | --- | --- | --- |
| Bahrain | 6,200 | 57,000 | 63,000 | 63,000 |
| Iran | 140,000 | 42,000 | 180,000 | 135,236 |
| Iraq | - | - | - | - |
| Kuwait | 1,400 | 28,000 | 30,000 | 30,000 |
| Oman | 35,000 | 3,100 | 38,000 | 1,556 |
| Qatar | 5,400 | 2,400 | 7,900 | 7,900 |
| Saudi Arabia | 6,300 | 11,000 | 17,000 | 8,552 |
| United Arab Emirates | 20,000 | 2,500 | 22,000 | 22,000 |

Table 5. Proportion of economically active population (%) in the fishery sector. Data from LABORSTA [8]

|  | | **Employment in fishing out of total economic activities (%)** | |
| --- | --- | --- | --- |
| **Country** | **Year** | **In all areas** | **In Gulf** |
| Bahrain | 2001 | 0.76 | 0.76 |
| Iran | 2008 | 0.38 | 0.29 |
| Iraq | - | - | - |
| Kuwait | 2005 | 0.08 | 0.08 |
| Oman | 2000 | 2.57 | 0.11 |
| Qatar | 2004 | 0.42 | 0.42 |
| Saudi Arabia | 2008 | 0.21 | 0.11 |
| United Arab Emirates | 2008 | 0.33 | 0.33 |

As a note, this indicator does not differentiate between inland and marine fisheries. Thus, in Iraq, which has a very short coastline and substantial riverine fisheries, it likely over-represents the labour force involved in marine fisheries. On the other hand, this indicator does not account for informal participation in fisheries by children and women, whose contributions are substantial in many parts of the world [9, 10], as well as part-time fishers (i.e., subsistence fishing).

##### Nutritional dependence

The nutritional dependence of a country on fisheries is represented by each country’s *dependence on fish as a source of protein*, and the *percentage of malnourished children*. The indicator of fish protein as percentage of all animal protein reflects the contribution of fish to a country’s food security. We assume that a higher percentage of fish protein in people’s diet equates with higher reliance on fish for food security. A country with high reliance on fish for food security would be more sensitive to changes in food fish supply. The percentage of fish out of all animal protein consumed for each Gulf country was obtained from FAO statistics [11] (Table 6).

Table 6. Fish protein as proportion (%) of all animal protein. Data from FAOSTAT [11]

|  | **Fish out of animal proteins (%) in 2011** | |
| --- | --- | --- |
| **Country** | **Overall** | **In Gulf** |
| Bahrain | - | - |
| Iran | 9.87 | 7.42 |
| Iraq | 6.56 | 6.56 |
| Kuwait | 7.77 | 7.77 |
| Oman | 8.20 | 0.34 |
| Qatar | - | - |
| Saudi Arabia | - | - |
| United Arab Emirates | 14.59 | 14.59 |

Bahrain, Qatar and Saudi Arabia have no data available on the FAO website. Among those countries with data, the UAE has the highest dependence on fish as their animal protein source (14.6%), whereas Iraq has the lowest dependence (6.6%).

As a note, fish protein as percentage of all animal protein does not account for fish substitutability, that is, the degree to which people are willing and able to substitute fish with another source of protein. High fish substitutability could thus reduce sensitivity.

The percentage of malnourished children is represented by the percentage of children under the age of five who were underweight. These data were obtained from the World Health Organization [12] and cover different years within the period of 1995 to 2014 for the assessed countries (Table 7).

Table 7. Rate of child malnutrition. Data from WHO [12]

| **Country** | **Year** | **Children under 5 who are underweight  (%, both sexes)** |
| --- | --- | --- |
| Bahrain | 1995 | 7.6 |
| Iran | 2004 | 4.6 |
| Iraq | 2011 | 8.5 |
| Kuwait | 2014 | 3 |
| Oman | 2009 | 8.6 |
| Qatar | 1995 | 4.8 |
| Saudi Arabia | 2004-2005 | 5.3 |
| United Arab Emirates | - | - |

These data are used as an indicator of whether nutrition provided by local foods, including from fisheries, is sufficient to support the health of people in a country. The growth of children is widely used as an indicator of the nutritional status of a society and is also considered as an important public-health indicator for monitoring well-being in populations [12]. We did not find any data on the percentage of malnourished children for the UAE. Among all of the Gulf countries, Oman has the highest percentage of malnourished children (8.6%) and Kuwait the lowest (3%) (Table 7).

##### Economic dependence

The dependence of a country’s economy on the fisheries sector was estimated for each country from several variables, including: landed values as a proportion of total Gross Domestic Product (GDP); fisheries export value as a proportion of total export value; total fisheries landings; and poverty rate.

Landed values are calculated as the product of ex-vessel price of each marine taxa [13, 14] and total catch by a country. Therefore, the *proportion of landed values to total GDP* can be used to ascertain a country’s dependence on its fisheries sector as a source of revenue. Bahrain had the highest proportion of landed values to total GDP (1%), whereas Saudi Arabia had the lowest (0.065%) (Table 8).

Table 8. ****Gulf countries’ dependence on the fishery sector as a source of revenue****

|  |  | **Average annual landed value  (US$ million)^2^** | | **Proportion of landed values to total GDP (%)** | |
| --- | --- | --- | --- | --- | --- |
| **Country** | **Average GDP (US$ billion)^1^** | **Overall** | **In Gulf** | **Overall** | **In Gulf** |
| Bahrain | 23 | 238 | 238 | 1.04 | 1.04 |
| Iran^3^ | 372 | 631 | 474 | 0.17 | 0.13 |
| Iraq | 107 | 118 | 118 | 0.11 | 0.11 |
| Kuwait | 117 | 82 | 82 | 0.07 | 0.07 |
| Oman^3^ | 49 | 417 | 17 | 0.84 | 0.03 |
| Qatar | 96 | 67 | 67 | 0.07 | 0.07 |
| Saudi Arabia^3^ | 454 | 296 | 149 | 0.07 | 0.03 |
| United Arab Emirates | 267 | 213 | 213 | 0.08 | 0.08 |

^1^ Gross Domestic Product (GDP) was obtained from The World Bank [15] and represents the average from 2006 to 2010.

^2^ Landed value is the ex-vessel price data from Sea Around Us price database [13, 14] multiplied by the total catch from the Sea Around Us database ([www.seaaroundus.org](http://www.seaaroundus.org)) [16]. Data shown are average annual landed values from 2006 to 2010.

Dependence of a country’s economy on its fisheries sector can also be estimated from *fisheries’ export value as a percentage of total export value* (Table 9). Countries that rely heavily on the fishery sector to generate national income through exports are especially sensitive to fisheries decline. Note that this indicator likely does not capture the value of marine products such as shark fins that are often traded on the black market or transacted at sea.

Table 9. Fisheries export value (as proportion (%) of total export value. Data from FAO [11, 17] and the UN [18].

|  |  | **Fisheries exports as % of total trade exports (USD)** | |
| --- | --- | --- | --- |
| **Country** | **Year** | **In all areas** | **In Gulf** |
| Bahrain | 2003 | 0.17 | 0.17 |
| Iran | 2003 | 0.20 | 0.15 |
| Iraq | 2003 | 0.001 | 0.001 |
| Kuwait | - | - | - |
| Oman | 2013 | 0.26 | 0.01 |
| Qatar | 2001 | 0.02 | 0.02 |
| Saudi Arabia | 2003 | 0.03 | 0.01 |
| United Arab Emirates | 2002 | 0.12 | 0.12 |

*Total fisheries landings* is the total quantity of fish catch taken from a country’s EEZ, including fish legally and illegally caught by subsistence, commercial and recreational fishers, and including fish discards (Table 10).

Table 10. Total fisheries catch by each Gulf country (average for 2006 to 2010). Data from Pauly and Zeller [16]

|  | **Annual catch (tonnes), average from 2006 – 2010** | |
| --- | --- | --- |
| **Country** | **Overall** | **In Gulf** |
| Bahrain | 38,600 | 38,600 |
| Iran | 162,120 | 121,803 |
| Iraq | 12,869 | 12,869 |
| Kuwait | 38,692 | 38,692 |
| Oman | 90,089 | 3,688 |
| Qatar | 12,072 | 12,072 |
| Saudi Arabia | 39,502 | 19,873 |
| United Arab Emirates | 39,508 | 39,508 |

There are many working definitions of ‘poverty’ with considerable debate on how to best define and measure the term (e.g., % of the population living below PPP$1.25 a day; working poor at PPP$2 a day (% of total employment)). We chose here to measure poverty as the percentage of a country’s population living below the national poverty line - the poverty line deemed appropriate for a country by its authorities. National estimates are typically based on population-weighted subgroup estimates from household surveys. While there are some caveats surrounding this indicator, notably that poverty line standards vary considerably among countries, it was the only statistic for which values were available. Even then, they were only available from a varied number of sources [19-22] and no data were available for Kuwait, Oman and Qatar (Table 11).

Table 11. Poverty headcount ratio below national poverty line

| **Country** | **Year** | **Population below national poverty line (%)** | **Sources** |
| --- | --- | --- | --- |
| Bahrain | 2002 - 2012 | 11 | El-Khoury [21] |
| Iran | 2007 | 18.7 | CIA [20] |
| Iraq | 2006 | 22.9 | El-Khoury [21] |
| Kuwait | - | - | - |
| Oman | - | - | - |
| Qatar | - | - | - |
| Saudi Arabia | 2013 | 12.7 | Al Arabiya [19] |
| United Arab Emirates | 2003 | 19.5 | NationMaster [22] |

We assumed that the higher the poverty rate (regardless of how national poverty lines were defined at individual country level), the more sensitive a country would be to climate-related impacts. For those countries for which data could be obtained, Iraq registered the highest percentage of population below national poverty line (22.9%) and Bahrain the lowest (11%).

##### Marine ecosystem services

The physical well-being of coastal communities is crucial for fisheries to thrive and contribute to national economies and food security. Here, we used two metrics, *land area at low elevation* and *population density at low elevation*, to gauge the sensitivity of coastal communities to climate change.

Table 12. Country’s dependence on marine ecosystem for coastal protection. Data from World Bank [15]

|  | **Land area where elevation is below 5 meters^1^** | **Population living in areas where elevation is below 5 meters** |
| --- | --- | --- |
| **Country** | **% of land area** | **% of total population** |
| Bahrain | 39 | 66.6 |
| Iran | 1.6 | 5 |
| Iraq | 4 | 6.5 |
| Kuwait | 8.9 | 22.8 |
| Oman | 1.1 | 5.5 |
| Qatar | 13.4 | 23.1 |
| Saudi Arabia | 0.5 | 1 |
| United Arab Emirates | 4.6 | 7.3 |

^1^ Refers to land adjacent to the coast and does not include inland locations not subject to inundation

Climate change is expected to cause sea level rise and to increase the frequency and severity of storms that can badly damage fisheries infrastructure (boats, ports, houses). Coastal land areas that are at low elevation are especially at risk of such hazards. Impacts will be exacerbated if high population densities live in such low-lying areas. We thus assumed that the larger the coastal land area a country has at low elevation (defined as <5 m), and the higher the population density living in these coastal low-lying areas, the higher that country’s sensitivity to climate change (Table 12).

#### Adaptive capacity

Adaptive capacity in the context used here, describes the ability of a system associated with a given planning area to effectively accommodate changes in climate with minimum disruption or minimum additional costs. We defined adaptive capacity through a composite indicator consisting of four human development indices *health, education, fisheries management, governance, size of the economy* and *economic diversity/complexity,* themselves composed of different sets of individual variables. The underlying assumption is that countries with higher levels of economic and human development have a greater capacity to adapt to the risks posed by climate change. The four human development indices are normalized between zero and 1 and standardized. The adaptive capacity index is computed by averaging these six standardized human development variables for each country and then subtracting this value from 1. This was done as we assumed countries with greater adaptive capacity to be less vulnerable to climate change impacts (Table 13).

Table 13. Adaptive capacity index and the standardized values of its composite indicators

|  | **Health** | **Education** | | **Governance**^1^ | | | | | | **Fisheries management** | **Size of economy** | **Employment alternatives** |  |  |
| --- | --- | --- | --- | --- | --- | --- | --- | --- | --- | --- | --- | --- | --- | --- |
| **Country** | **Life Expectancy** | **Adult literacy rate** | **Enrolment ratio (tertiary)** | **PS** | **GE** | **RQ** | **RL** | **VA** | **CC** | **MPA** | **GDP** | **Economic diversity** | **Average of all indexes** | **Adaptive capacity Index (ranking)** |
| Bahrain | 0.79 | 0.88 | 0.44 | 0.44 | 0.66 | 0.88 | 0.77 | 0.41 | 0.64 | 0.35 | 0.00 |  | 0.57 | 0.43 **(5)** |
| Iran | 0.63 | 0.30 | 0.95 | 0.45 | 0.28 | 0.00 | 0.14 | 0.19 | 0.30 | 0.07 | 0.55 | 0.00 | 0.32 | 0.68 **(2)** |
| Iraq | 0.00 | 0.00 | 0.04 | 0.00 | 0.00 | 0.09 | 0.00 | 0.51 | 0.00 | 0.00 | 0.27 |  | 0.08 | 0.92 **(1)** |
| Kuwait | 0.56 | 0.93 | 0.33 | 0.75 | 0.38 | 0.55 | 0.60 | 1.00 | 0.42 | 0.01 | 0.18 | 0.65 | 0.53 | 0.47 **(4)** |
| Oman | 0.82 | 0.45 | 0.32 | 0.90 | 0.54 | 0.88 | 0.83 | 0.64 | 0.62 | 0.00 | 0.07 | 0.26 | 0.53 | 0.47 **(3)** |
| Qatar | 1.00 | 1.00 | 0.00 | 1.00 | 0.81 | 0.83 | 1.00 | 0.71 | 0.95 | 0.04 | 0.25 | 0.23 | 0.65 | 0.35 **(7)** |
| Saudi Arabia | 0.53 | 0.87 | 1.00 | 0.64 | 0.52 | 0.60 | 0.69 | 0.00 | 0.56 | 0.12 | 1.00 | 1.00 | 0.63 | 0.37 **(6)** |
| United Arab Emirates | 0.86 | 0.62 | - | 0.95 | 1.00 | 1.00 | 0.88 | 0.64 | 1.00 | 1.00 | 0.51 | 0.95 | 0.86 | 0.14 **(8)** |

^1^ PS – Government stability; GE – Government effectiveness; RQ – Regulatory quality; RL – Rule of law; VA – Voice and accountability; CC – Control of corruption

##### Health

Countries with a majority of people in good health are assumed to be able to allocate more resources to mitigate and adapt to climate change impacts. We used *life expectancy at birth*, defined as the number of years a newborn infant could expect to live if prevailing patterns of age-specific mortality rates at the time of birth stay the same throughout the infant’s life (UNDP 2015), as an indicator of health. Life expectancy at birth summarizes the mortality pattern that prevails across all age groups in a given year – children and adolescents, adults and the elderly. Estimates were derived for both male and females and based on data made available by the World Bank [15] and UNDP [23]. Among Gulf countries, Qatar had the highest life expectancy (78.2 years) while Iraq ranked lowest (69.4 years) in 2013 (Table 14).

Table 14. Health and education indicators

|  | **Health** | **Education** | |
| --- | --- | --- | --- |
| **Country** | **Life expectancy at birth (2013)**^1^ | **Adult literacy rate  (% ages 15 and older)**^2^ | **Enrolment ratio (tertiary)**^2^ |
| Bahrain | 76.55 | 94.6 | 33.5 |
| Iran | 75.13 | 84.3 | 55.2 |
| Iraq | 69.47 | 79 | 16 |
| Kuwait | 74.46 | 95.5 | 28.5 |
| Oman | 76.84 | 86.9 | 28.1 |
| Qatar | 78.42 | 96.7 | 14.3 |
| Saudi Arabia | 74.18 | 94.4 | 57.5 |
| United Arab Emirates | 77.20 | 90 | - |

^1^ Life expectancy at birth data was obtained from the World Bank [15] and UNDP [23]

^2^ Adult literacy rate and enrolment ratio were obtained from UNDP [23]

Given the lack of complete and reliable mortality data, especially for low income countries and particularly among adults and the elderly, life expectancy estimates are often derived using models (based on data from other populations) [24]{WHO, 2016 #4733}. While the method is standardised, this may lead to minor differences compared with official life tables prepared by Member States. Another variable often used as an indicator of health is the “health-adjusted life expectancy (HALE) at birth”, defined as the average number of years that an individual can expect to live in a healthy state. It is a summary measure that combines mortality and morbidity experience into a single summary measure of population health. It includes the burden of disease and injury in the population, risk factors, and the performance of public health efforts [25]. This parameter also suffers from a clear lack of reliable data on mortality and morbidity estimates, as well as a lack of comparability of self-reported data from health interviews and the measurement of health-state preferences for such self-reporting. However, it is interesting to note that the trends observed are the same as when using life expectancy at birth.

##### Education

The educational indicator was estimated using two variables: adult literacy rate and tertiary level enrolment ratio. *Adult literacy rate* is defined as the percentage of the population aged 15 and above who can, with understanding, read and write a short, simple statement on their everyday life. Generally, ‘literacy’ also encompasses ‘numeracy’, the ability to make simple arithmetic calculations. This indicator is calculated by dividing the number of literates aged 15 years and over by the corresponding age group population and multiplying the result by 100 [15]. The *total enrolment in tertiary education* (ISCED 5 to 8), regardless of age, is expressed as a percentage of the total population of the five-year age group following on from secondary school leaving [15]. Arguably, secondary enrolment ratio or a composite of primary, secondary and tertiary enrolment ratio might be more appropriate, but data for those segments is not always available.

The general number of people with education and the proportion of these individuals with higher education are assumed to lend a country greater and more effective adaptive capacity to climate change (Table 14). To face climate change effectively, basic education in literacy and numeracy as well as fast and efficient continued learning are critical. We felt that these two variables complemented each other to give an indicator that would measure a core set of capabilities promoting adaptive capacity successfully.

##### Governance

Governance is understood, broadly, as encompassing institutions (both state and non-state) and their functioning, as well as the processes underpinning people’s decision-making (e.g., collective action, power relations, networks, and individual incentives). More specifically, it is defined as consisting of the traditions and institutions by which authority in a country is exercised; including the process by which governments are selected, monitored and replaced; the capacity of the government to effectively formulate and implement sound policies; and the respect of citizens and the state for the institutions that govern economic and social interactions among them [15]. Governance is prevalent throughout the discourse on climate change adaptation, and, along with institutions and institutional mechanisms, is seen as a critical determinant of adaptation [26-28]. We based our framework to measure governance on the World Bank’s Worldwide Governance Indicators project (www.govindicators.org/). The project reports aggregate indicators for six dimensions of governance [29] considered as useful measures for broad cross-country comparisons:

1. *Voice and Accountability*: captures perceptions of the extent to which a country's citizens are able to participate in selecting their government, as well as freedom of expression, freedom of association, and a free media (Table 15).
2. *Political Stability and Absence of Violence/Terrorism*: measures perceptions of the likelihood of political instability and/or politically-motivated violence, including terrorism (Table 16).
3. *Government Effectiveness*: captures perceptions of the quality of public services, the quality of the civil service and the degree of its independence from political pressures, the quality of policy formulation and implementation, and the credibility of the government's commitment to such policies (Table 17).
4. *Regulatory Quality*: captures perceptions of the ability of the government to formulate and implement sound policies and regulations that permit and promote private sector development (Table 18).
5. *Rule of Law*: captures perceptions of the extent to which agents have confidence in and abide by the rules of society, and in particular the quality of contract enforcement, property rights, the police, and the courts, as well as the likelihood of crime and violence (Table 19).
6. *Control of Corruption*: captures perceptions of the extent to which public power is exercised for private gain, including both petty and grand forms of corruption, as well as "capture" of the state by elites and private interests (Table 20).

Table 15. Voice and accountability [29]. SE= Standard Error

|  | **2014** | | | | | |
| --- | --- | --- | --- | --- | --- | --- |
| **Country** | **Estimate** | **SE** | **Number of data sources** | **Rank** | **Lower** | **Upper** |
| Bahrain | -1.32 | 0.14 | 7 | 11.33 | 5.39 | 17.65 |
| Iran | -1.57 | 0.13 | 9 | 4.93 | 3.43 | 9.80 |
| Iraq | -1.21 | 0.14 | 9 | 13.79 | 7.84 | 22.06 |
| Kuwait | -0.65 | 0.14 | 8 | 29.06 | 24.51 | 34.31 |
| Oman | -1.05 | 0.14 | 7 | 19.70 | 12.25 | 26.47 |
| Qatar | -0.98 | 0.14 | 10 | 22.17 | 14.22 | 26.96 |
| Saudi Arabia | -1.78 | 0.14 | 9 | 3.45 | 1.96 | 5.39 |
| United Arab Emirates | -1.06 | 0.13 | 9 | 19.21 | 12.25 | 25.00 |

Table 16. Political Stability and Absence of Violence/Terrorism [29].
SE= Standard Error

|  | **2014** | | | | | |
| --- | --- | --- | --- | --- | --- | --- |
| **Country** | **Estimate** | **SE** | **Number of data sources** | **Rank** | **Lower** | **Upper** |
| Bahrain | -0.94 | 0.21 | 7 | 14.56 | 8.70 | 25.60 |
| Iran | -0.91 | 0.20 | 8 | 16.99 | 9.66 | 26.09 |
| Iraq | -2.47 | 0.22 | 6 | 2.43 | 0.00 | 5.31 |
| Kuwait | 0.14 | 0.21 | 7 | 52.43 | 37.68 | 62.32 |
| Oman | 0.66 | 0.21 | 7 | 67.96 | 57.97 | 83.57 |
| Qatar | 1.00 | 0.20 | 8 | 83.01 | 68.60 | 97.10 |
| Saudi Arabia | -0.24 | 0.21 | 7 | 35.44 | 25.60 | 51.21 |
| United Arab Emirates | 0.81 | 0.20 | 9 | 75.73 | 62.80 | 90.34 |

Table 17. Government effectiveness [29]. SE= Standard Error

|  | **2014** | | | | | |
| --- | --- | --- | --- | --- | --- | --- |
| **Country** | **Estimate** | **SE** | **Number of data sources** | **Rank** | **Lower** | **Upper** |
| Bahrain | 0.59 | 0.23 | 7 | 72.60 | 61.72 | 77.99 |
| Iran | -0.41 | 0.23 | 7 | 37.98 | 21.05 | 53.11 |
| Iraq | -1.13 | 0.22 | 8 | 13.94 | 5.74 | 22.01 |
| Kuwait | -0.15 | 0.23 | 7 | 47.60 | 31.58 | 62.20 |
| Oman | 0.29 | 0.23 | 7 | 63.94 | 49.76 | 73.21 |
| Qatar | 0.99 | 0.22 | 8 | 78.37 | 72.73 | 87.56 |
| Saudi Arabia | 0.23 | 0.23 | 7 | 62.02 | 46.41 | 72.73 |
| United Arab Emirates | 1.48 | 0.22 | 8 | 90.38 | 82.30 | 97.61 |

Table 18. Regulatory quality [29]. SE= Standard Error

|  | **2014** | | | | | |
| --- | --- | --- | --- | --- | --- | --- |
| **Country** | **Estimate** | **SE** | **Number of data sources** | **Rank** | **Lower** | **Upper** |
| Bahrain | 0.70 | 0.23 | 7 | 74.04 | 62.68 | 81.82 |
| Iran | -1.46 | 0.22 | 8 | 4.81 | 2.87 | 12.44 |
| Iraq | -1.25 | 0.21 | 7 | 9.13 | 4.78 | 19.62 |
| Kuwait | -0.13 | 0.23 | 7 | 48.56 | 32.54 | 61.72 |
| Oman | 0.69 | 0.23 | 7 | 73.56 | 62.68 | 81.82 |
| Qatar | 0.57 | 0.22 | 8 | 70.67 | 59.33 | 79.43 |
| Saudi Arabia | -0.01 | 0.23 | 7 | 53.37 | 38.28 | 65.55 |
| United Arab Emirates | 0.98 | 0.21 | 9 | 80.29 | 71.77 | 89.47 |

Table 19. Rule of law [29]. SE= Standard Error

|  | **2014** | | | | | |
| --- | --- | --- | --- | --- | --- | --- |
| **Country** | **Estimate** | **SE** | **Number of data sources** | **Rank** | **Lower** | **Upper** |
| Bahrain | 0.45 | 0.18 | 9 | 68.27 | 63.64 | 76.56 |
| Iran | -1.03 | 0.17 | 10 | 13.94 | 7.66 | 24.40 |
| Iraq | -1.36 | 0.18 | 9 | 5.77 | 1.91 | 13.40 |
| Kuwait | 0.05 | 0.18 | 9 | 60.10 | 47.37 | 67.46 |
| Oman | 0.58 | 0.18 | 8 | 73.08 | 65.55 | 77.99 |
| Qatar | 0.99 | 0.17 | 10 | 81.25 | 76.08 | 86.12 |
| Saudi Arabia | 0.27 | 0.18 | 9 | 65.38 | 56.94 | 72.25 |
| United Arab Emirates | 0.71 | 0.16 | 11 | 76.44 | 67.94 | 80.38 |

Table 20. Control of corruption [29]. SE= Standard Error

|  | **2014** | | | | | |
| --- | --- | --- | --- | --- | --- | --- |
| **Country** | **Estimate** | **SE** | **Number of data sources** | **Rank** | **Lower** | **Upper** |
| Bahrain | 0.30 | 0.18 | 7 | 64.42 | 57.42 | 70.33 |
| Iran | -0.57 | 0.16 | 8 | 34.62 | 21.05 | 47.85 |
| Iraq | -1.34 | 0.18 | 9 | 5.77 | 0.96 | 13.88 |
| Kuwait | -0.26 | 0.18 | 6 | 50.00 | 35.41 | 57.89 |
| Oman | 0.25 | 0.18 | 6 | 62.98 | 56.46 | 70.33 |
| Qatar | 1.09 | 0.16 | 8 | 82.69 | 76.08 | 89.95 |
| Saudi Arabia | 0.10 | 0.18 | 7 | 59.62 | 51.67 | 66.99 |
| United Arab Emirates | 1.23 | 0.15 | 8 | 84.13 | 79.90 | 90.43 |

These indicators combine the views of a large number of enterprise, citizen and expert survey respondents in industrial and developing countries. They are based on over 30 individual data sources produced by a variety of survey institutes, think tanks, non-governmental organizations, international organizations, and private sector firms [15].

We assumed that countries with high governance scores would be more responsive and flexible to adapt and therefore have a greater capacity to anticipate, and act on, changes due to climate stresses as well as implement the measures required. Governance can be a pivotal and conditioning factor in the resilience and adaptive capacity of aquatic social-ecological systems facing climate change. The importance of governance was underlined in the findings of Hughes *et al.* [30] with regard to the role of social organization in the adaptive capacity of reef fishing communities, and of Keskitalo and Kulyasova [31, p.60], who showed that for small-scale fishers, “…adaptive capacity beyond the immediate economic adaptations available to local actors is, to a considerable extent, politically determined within larger governance networks.” It also emerged as an overarching and conditioning factor in the analysis of six regional studies of climate change vulnerability in fisheries because of its influence on adaptation in terms of efficiency and equity [32]. Therefore, while a simple additive overall indicator was developed here, in line with a number of studies assessing the vulnerability of fisheries worldwide, future studies may wish to develop an indicator lending governance more weight. Also, operational legislative and regulatory frameworks are aspects of governance that can be influential in structuring effective adaptation. Thus, variables that representatively encompass and characterize this aspect may warrant integration in future climate-change vulnerability and adaptation assessments.

##### Fisheries management

Fisheries management is represented here by the presence and extent of *Marine Protected Areas* (MPA). Specifically this variable is included as the percentage of area that is protected for a given country’s EEZ (within the Gulf) (Table 21). While MPAs are designated for a broad variety of reasons, not necessarily related to fisheries management, it is the only indicator for which standardized information was available for all countries in the region. Irrespective of the specific objectives of declared MPAs, this indicator infers a country’s commitment to invest in their (future) natural capital. The assumption here is that the larger the MPA area, the more resources a country will invest/have invested in its management, and thus the higher the adaptive capacity. Note however that MPA coverage does not reflect the effectiveness of MPAs, often linked to a country’s capacity at enforcement. A high MPA coverage may be misleading if the MPAs are ‘paper parks’.

Table 21. Area included within a protected area as proportion of total area of EEZ*.* Data from IUCN and UN-WCMC [33]

| **Country** | **Marine area (km^2^)** | **Marine protected area (km^2^)** | **% of protected marine area** |
| --- | --- | --- | --- |
| Bahrain^1^ | 7,632 | 141 | 1.85 |
| Iran | 160,936 | 1,439 | 0.89 |
| Iraq | 627 | 0 | 0 |
| Kuwait | 11,901 | 11 | 0.09 |
| Oman | 539,039 | 0 | 0 |
| Qatar | 31,981 | 172 | 0.54 |
| Saudi Arabia^2^ | 219,853 | 2,100 | 0.96 |
| United Arab Emirates | 53,143 | 10,008 | 18.83 |

^1^ For most countries, total marine protected area was calculated using IUCN & UN WCMC estimated marine portion of a designated protected area. For a number of reasons, these at times differ from reported areas. For Bahrain unfortunately, only reported marine areas were available, and used here [33].

^2^Similarly to Bahrain, no IUCN & UN WCMC marine estimate was available. Moreover, here only the total reported area information was available. We therefore assumed, in the absence of available boundary information that would permit independent estimates, that all of the MPA is marine.

Individual fishing quotas (IFQs) also known as "*individual transferable quotas*" (ITQs) are a means for governments to regulate fishing activities. Studies conducted in the US claim that ITQ managed fisheries exhibit no long-term ecological advantages when compared to other modern fishery management schemes [34]. However, the general assumption is that catch share fisheries are less likely to fail when compared to open-access fisheries, in other words that ITQs can help prevent stock collapses and restore declining fisheries [35]. While we sought to include the proportion of stocks regulated by an ITQ system for a given country as a variable for the fisheries management indicator, we could not find any evidence for catch shares being implemented in any country in the region. The only information we did come across is a study conducted in 2010 looking at the possibility of introducing ITQs to manage fisheries in Oman [36].

##### Size of the economy

Countries with stronger economies (i.e., higher GDP) are assumed to be able to adapt better to climate change impacts. GDP is the sum of gross value added by all resident producers in the economy plus any product taxes and minus any subsidies not included in the value of the products. We obtained the GDP data from the World Bank [15] and the data are in current U.S. dollars. The dollar figures for GDP available on the World Bank website are converted from domestic currencies using single year official exchange rates. We used the most current year of data (i.e., 2004) from the website. Among these countries, Saudi Arabia had the highest GDP (US$746 billion), whereas Bahrain had the lowest (US$ 34 billion) (Table 22).

Table 22. Total Gross Domestic Product (USD) in 2014 [15] and economic diversity in terms of economic complexity rankings in 2013 [37]

| **Country** | **Total GDP in 2014 (US$ billion)** | **Economic Complexity Rankings (2013)** |
| --- | --- | --- |
| Bahrain | 33.85 | - |
| Iran | 425.33 | -0.73894 |
| Iraq | 223.51 | N/A |
| Kuwait | 163.61 | -0.0118 |
| Oman | 81.80 | -0.44662 |
| Qatar | 210.11 | -0.48073 |
| Saudi Arabia | 746.25 | 0.375092 |
| United Arab Emirates | 399.45 | 0.3147 |

##### Employment alternatives

The provision of alternative employment opportunities, measured here through *economic diversity* (or complexity), allows a society to better adapt to the public impacts caused by climate change. *Economic complexity* represents the mix of knowledge that is required to produce the range of products that a country makes. It is an indicator of the amount of knowledge embedded within an economy, as measured by the diversity and ubiquity of the products a country makes [38]. A country’s productive diversity refers to the number of distinct products it makes - countries with more embedded knowledge are capable of producing a more diverse range of products. Ubiquity refers to the number of countries that make a product – the more knowledge that is required to produce a product, the less ubiquitous is that product. We infer that countries with high economic complexity have a workforce with a diverse knowledge base and skillset that is better able to adapt to changes in the economy, such as a downturn in the fishing sector (Table 22). National export statistics were used to calculate economic complexity, thus only goods (not services) that a country exports were considered. Note that this indicator does not necessarily reflect the adaptive capacity of fisheries workers. Poor fishers in particular are often excluded from participating in the formal economy due to socio-economic marginalisation.

Knowledge availability, generally, is recognized as an important determinant of adaptation, which is why we sought to include it as an indicator contributing to the overall adaptive capacity score. Unfortunately, no standardized data were found to populate the variable chosen “*Budget in marine research and marine resource protection programme*”.

#### References

1. Al-Abdulrazzak D, Pauly D, editors. From dhows to trawlers: a recent history of fisheries in the Gulf countries, 1950 to 2010: Fisheries Centre Research Reports 21(2). University of British Columbia Fisheries Centre, Vancouver 2013.

2. Cheung WWL, Close C, Lam V, Watson R, Pauly D. Application of macroecological theory to predict effects of climate change on global fisheries potential. Mar Ecol Prog Ser. 2008;365:187-97. doi: 10.3354/meps07414. PubMed PMID: WOS:000259379600017.

3. Cheung WWL, Jones MC, Reygondeau G, Stock CA, Lam VWY, Frolicher TL. Structural uncertainty in projecting global fisheries catches under climate change. Ecol Modell. 2016;325:57-66. doi: 10.1016/j.ecolmodel.2015.12.018. PubMed PMID: WOS:000371944400006.

4. Jones MC, Cheung WWL. Multi-model ensemble projections of climate change effects on global marine biodiversity. Ices Journal of Marine Science. 2015;72(3):741-52. doi: 10.1093/icesjms/fsu172. PubMed PMID: WOS:000351837500002.

5. Intergovernmental Panel on Climate Change (IPCC), editor. Climate change 2001: impacts, adaptation and vulnerability. Contribution of Working Group II to the Third Assessment Report of the Intergovernmental Panel of Climate Change: Cambridge, UK, Cambridge University Press; 2001.

6. Allison EH, Perry AL, Badjeck MC, Adger WN, Brown K, Conway D, et al. Vulnerability of national economies to the impacts of climate change on fisheries. Fish and Fisheries. 2009;10(2):173-96. doi: 10.1111/j.1467-2979.2008.00310.x. PubMed PMID: WOS:000266024600005.

7. Teh LCL, Sumaila UR. Contribution of marine fisheries to worldwide employment. Fish and Fisheries. 2013;14(1):77-88. doi: 10.1111/j.1467-2979.2011.00450.x.

8. LABORSTA. LABORSTA database, International Labour Office Department of Statistics [Internet]. Downloaded from United Nations Statistics Division. 2016 [cited 18 May 2016]. Available from: <http://data.un.org/Data.aspx?d=LABORSTA&f=tableCode%3a2B>.

9. Harper S, Zeller D, Hauzer M, Pauly D, Sumaila UR. Women and fisheries: Contribution to food security and local economies. Mar Policy. 2013;39:56-63. doi: <https://doi.org/10.1016/j.marpol.2012.10.018>.

10. WorldFish, editor Global Symposium on Women in Fisheries: Sixth Asian Fisheries Forum2002; 29 November 2001, Kaohsiung, Taiwan.

11. FAOSTAT. FAO Statistical Databases 2015 [cited 10 May 2016]. Available from: <http://faostat3.fao.org/browse/FB/FBS/E>.

12. WHO. Global Health Observatory data repository World Health Organization; 2015 [cited 5 March 2016]. Available from: <http://apps.who.int/gho/data/node.main.1098?lang=en>

13. Sumaila UR, Marsden AD, Watson R, Pauly D. A global ex-vessel fish price database: construction and applications. Journal of Bioeconomics. 2007;9(1):39-51.

14. Swartz W, Sumaila R, Watson R. Global Ex-vessel Fish Price Database Revisited: A New Approach for Estimating 'Missing' Prices. Environ Resour Econ. 2013;56(4):467-80. doi: 10.1007/s10640-012-9611-1. PubMed PMID: WOS:000327855800002.

15. The Worldbank Group. World Bank Open Data 2016 [8 February 2016]. Available from: <http://data.worldbank.org/>

16. Pauly D, Zeller D. Catch reconstructions reveal that global marine fisheries catches are higher than reported and declining. Nature Communications. 2016;7. doi: ARTN 10244

10.1038/ncomms10244. PubMed PMID: WOS:000369018800007.

17. FishStat F. FAO 2011-2016. Fisheries and aquaculture software. FishStatJ - software for fishery statistical time series. [Internet]. FAO Fisheries and Aquaculture Department. 2016 [cited Accessed 18 May 2016]. Available from: <http://www.fao.org/fishery/statistics/software/fishstatj/en>.

18. UN Trade Statistics – United Nations. International Trade Statistics Yearbook. United Nations, New York, USA, 2015.

19. Al Arabiya. Saudi Arabia has tenth lowest poverty rate worldwide, says World Bank 2013 [cited 2016]. Available from: <http://english.alarabiya.net/en/business/economy/2013/11/03/Kingdom-has-tenth-lowest-poverty-rate-worldwide-says-World-Bank.html>.

20. CIA. The World FactBook 2016 2016 [cited 2016]. Available from: <https://www.cia.gov/library/publications/the-world-factbook/fields/2046.html>.

21. El-Khoury G. Selected socio-economic indicators in Arab countries. Contemporary Arab Affairs. 2013;6(4):14. doi: doi: 10.1080/17550912.2013.843741.

22. NationMaster. Population below poverty line: countries compared 2016 [cited 4 April 2016]. Available from: <http://www.nationmaster.com/country-info/stats/Economy/Population-below-poverty-line>.

23. UNDP. Human Development Report. Work for Human Development. United Nations Development Program, New York., 2015.

24. WHO. Indicator and measurement registry: World Health Organization; 2016 [16 March 2016]. Available from: <http://apps.who.int/gho/indicatorregistry/App_Main/browse_indicators.aspx>.

25. Mathers CD, Murray CJL, Salomon J. Methods for Measuring Healthy Life Expectancy. In: Murray CJL, Evans D, editors. Health systems performance assessment: debates, methods and empiricism: World Health Organization. Geneva, Switzerland.; 2003.

26. Adger WN, Dessai S, Goulden M, Hulme M, Lorenzoni I, Nelson DR, et al. Are there social limits to adaptation to climate change? Climatic Change. 2009;93(3-4):335-54. doi: 10.1007/s10584-008-9520-z. PubMed PMID: WOS:000264477100004.

27. Biesbroek GR, Termeer CJAM, Klostermann JEM, Kabat P. Analytical lenses on barriers in the governance of climate change adaptation. Mitigation and Adaptation Strategies for Global Change. 2014;19(7):1011-32. doi: 10.1007/s11027-013-9457-z. PubMed PMID: WOS:000342180000006.

28. Engle NL, Lemos MC. Unpacking governance: Building adaptive capacity to climate change of river basins in Brazil. Global Environ Chang. 2010;20(1):4-13. doi: 10.1016/j.gloenvcha.2009.07.001. PubMed PMID: WOS:000274672500002.

29. Kaufman D, Kraay A, Mastruzzi M. The Worldwide Governance Indicators: Methodology and Analytical Issues. World Bank Policy Research Working Paper No. 5430. The WorldBank, Washington D.C., USA, 2010.

30. Hughes S, Yau A, Max L, Petrovic N, Davenport F, Marshall M, et al. A framework to assess national level vulnerability from the perspective of food security: The case of coral reef fisheries. Environ Sci Policy. 2012;23:95-108. doi: 10.1016/j.envsci.2012.07.012. PubMed PMID: WOS:000310110700010.

31. Keskitalo ECH, Kulyasova AA. The role of governance in community adaptation to climate change. Polar Res. 2009;28(1):60-70. doi: 10.1111/j.1751-8369.2009.00097.x. PubMed PMID: WOS:000264242100005.

32. Brugère C. Climate change vulnerability in fisheries and aquaculture: a synthesis of six regional studies. FAO Fisheries Circular No. 1104. Rome, FAO: 2015.

33. IUCN and UN-WCMC. The World Database on Protected Areas (WDPA) [Internet]. Cambridge, UK: UN-WCMC. 2016 [cited 15 January 2016]. Available from: [www.protectedplanet.net](http://www.protectedplanet.net).

34. Essington TE. Catch Shares Improve Consistency, not Health, of Fisheries. Lenfest Ocean Program, 2009.

35. Costello C, Gaines SD, Lynham J. Can catch shares prevent fisheries collapse? Science. 2008;321(5896):1678-81. doi: 10.1126/science.1159478. PubMed PMID: WOS:000259300400036.

36. Kotagama H, Zekri S, Boughanmi H, Al-Jufaili SM. Feasibility of Individual Tradable Quotas (ITQ) to Manage Oman’s Fishery: Socio-Economic Analysis. . Final Report on Research Project. Economic and Institutional Aspects of Kingfish Management IG AGR/ECON/05/03. Sultan Qaboos University: 2010.

37. MIT. Economic complexity rankings 2016 [cited 5 March 2016]. Available from: <http://atlas.media.mit.edu/en/rankings/country/>.

38. Hausmann R, Hidalgo CA, Bustos S, Coscia M, Chung S, Jimenez J, et al. The Atlas of Economic Complexity: Mapping Paths to Prosperity 2014 [3 March 2016]. Available from: <http://atlas.media.mit.edu/static/pdf/atlas/AtlasOfEconomicComplexity.pdf>.
